# Interplay of Noncovalent Interactions in Phase Separation Mediated by Tyrosine-Rich and Arginine-Rich Polypeptides

**DOI:** 10.1021/jacs.6c06972

**Published:** 2026-06-17

**Authors:** Ruiwen Xu, Rui Wang, Cindy Qiu, Jiani Niu, Desiree M. Bates, Nicholas L. Abbott, Samuel H. Gellman

**Affiliations:** † Department of Chemistry, 5228 University of Wisconsin-Madison, Madison, Wisconsin 53706, United States; ‡ Robert F. Smith School of Chemical and Biomolecular Engineering, 5922 Cornell University, New York, Ithaca 14853, United States

## Abstract

Liquid–liquid phase separation (LLPS) and other forms of condensed phase formation play important roles in many cellular processes. Elucidating the noncovalent interaction networks that underlie condensate formation is a fundamental challenge. Cation–π interactions between Arg and Tyr side chains have been proposed as a driving force for many forms of protein-mediated condensate formation. Efforts to probe this hypothesis with ribosomally generated proteins are constrained by limits on residue incorporation. To transcend these limits, we developed a two-component system comprising a long Arg-rich protein fragment, generated via heterologous expression, and a short anionic Tyr-rich peptide, generated via chemical synthesis. Phase separation occurred when these components were mixed at low concentrations (5 μM each). Global replacements of Tyr with noncanonical residues were conducted to interrogate side chain contributions to condensate formation. The results suggest that cation–π interactions are not essential for phase separation in our system, and that even when cation–π interactions contribute, their role may not be dominant. H-bond donor properties of the Tyr side chain hydroxyl appear to play a significant role, along with Coulombic forces, in driving condensate formation mediated by the anionic Tyr-rich peptide and the Arg-rich protein fragment.

## Graphical abstract

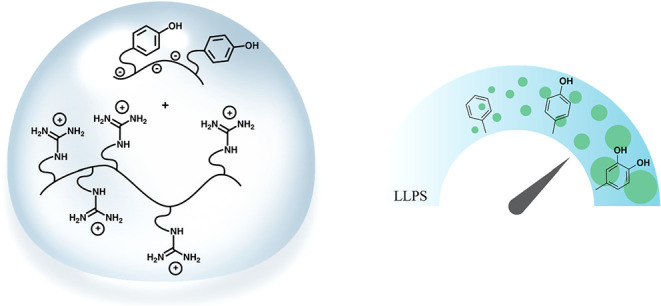

## Introduction

Spontaneous formation of condensed phases mediated by biopolymers is widespread within cells.^1–8^ These processes, which include liquid–liquid phase separation (LLPS), facilitate the formation of highly concentrated compartments in which macromolecular assemblies serve as scaffolds. These “membraneless organelles” can recruit or exclude specific molecules.^8–10^ Molecular sorting of specific proteins and/or nucleic acids appears to facilitate key physiological processes, such as ribosome assembly, RNA splicing or transcription.^1^ The functionality of fluid cellular condensates arises in part from the ease with which molecules can exchange between them and the surrounding medium.^2,3^ The absence of a defining lipid bilayer enables condensates to form and dissipate rapidly in response to diverse chemical or physical cues, allowing the cell to regulate its internal environment.^4^

Assembly of macromolecules in a manner that retains intra- and intermolecular dynamics requires a delicate balance of noncovalent forces. Formation of the macromolecular network that constitutes the condensate scaffold must be sufficiently favorable in terms of free energy to overcome the loss of translational entropy upon concentration, but interactions among the macromolecules should not be so strong as to cause formation of a gel or solid. Network formation can be stabilized by intrinsic intermolecular attractions, release of ordered water molecules and/or release of counterions. Some biomolecular condensation processes are mediated at least in part by interactions involving two or more polyvalent proteins that contain multiple modules with specific and complementary recognition properties.^1,8^ Many condensation phenomena, however, arise from interactions involving much smaller molecular fragments, such as individual amino acid residue side chains.^11–16^ The factors that drive this latter form of assembly remain a subject of active inquiry.^10,17^ The studies described here were intended to provide insight on these forces in a specific system.

Efforts to understand the noncovalent forces that mediate formation of intracellular condensates are important because this type of assembly has been associated with multiple diseases.^6,7,18^ Fundamental insights on the intermolecular interactions that drive condensate formation involving biopolymers and that influence the evolution of condensed liquid phases toward more ordered phases lay a foundation for efforts to modulate the underlying biomolecular associations for therapeutic purposes.

Proteins that mediate formation of RNA-rich condensates in cells have received considerable attention.^19^ FUS (Fused in Sarcoma) is regarded as representative of many proteins in this group.^18,20,21^ Condensates scaffolded by FUS facilitate key physiological functions of RNA including translation and splicing. Characteristics of FUS-family proteins include intrinsic disorder over much of the sequence and specific domains that are rich in either tyrosine or arginine.^18^ Associations involving the C-terminal Arg-rich domain and the N-terminal Tyr-rich domain of FUS appear to drive condensate formation by this protein; phase separation is maintained even if these domains are not covalently linked. Cation–π interactions between guanidinium groups in Arg side chains and aromatic rings in Tyr side chains have been proposed to make a major contribution to condensate formation mediated by FUS-family proteins;^20,21^ additional possible contributions of Arg and Tyr side chains have been more recently suggested.^15,22^

The contributions of individual side chains to the behavior of a ribosomally produced protein can be probed by replacing one proteinogenic α-amino acid residue with another. Experimental approaches involving nonproteinogenic residues are more challenging. Genetic code expansion via amber-suppression methods allows biosynthesis of proteins in which a single native side chain at a specific site is substituted with a nonproteinogenic side chain.^23,24^ However, use of nonproteinogenic residues to explore condensate formation would be most illuminating if substitution could be implemented at multiple sites, which requires chemical synthesis. Several reports have described phase separation mediated by a synthetic peptide alone, but very high concentrations (≥10 mM) are typically required.^25,26^ We sought a system that would display phase separation at low μM concentrations, as is typical of FUS and related proteins.^20,21^ Increasing polymer chain length generally enhances phase separation propensity.^27^

The studies described here focus on condensate formation resulting from combination of two components, an Arg-rich fragment of FUS, generated via heterologous expression, and a designed Tyr-rich peptide prepared by chemical synthesis. The length of the FUS fragment (154 residues) was intended to promote phase separation at low concentrations. Solid-phase synthesis of the shorter component enabled global replacement of Tyr with nonproteinogenic residues. Global replacement appears to be critical for phase separation studies: in FUS itself, global Tyr→Phe replacement in the N-terminal domain substantially reduces phase separation propensity,^20^ but partial Tyr→Phe replacement does not.^21^ Some amino acids with noncanonical side chains similar to those of proteinogenic amino acids can be ribosomally incorporated without genetic code expansion.^28^ However, the degree of incorporation can be variable within the mixture of polypeptides that is produced.^29^

Our experimental design was inspired by studies of cation–π interactions involving a specific side chain in discrete ligand-protein complexes.^30–38^ Ribosomal synthesis of proteins in which a single native aromatic side chain at a specific site was substituted with a noncanonical side chain allowed incisive evaluation of these cation–π interactions. For example, Tyr, which bears an electron-rich aromatic ring, was replaced with a residue bearing an electron-poor aromatic ring, such as 3,4,5-trifluorophenylalanine (F_3_Phe). An authentic cation–π interaction will be impaired or abolished upon Tyr→F_3_Phe modification, but interactions arising from other noncovalent factors will persist after this modification.^34–38^

In the studies described below, the synthetic peptide component bears a net negative charge, and Coulombic interactions between the peptide and the Arg-rich polypeptide (including counterion release upon polyanion-polycation association) contribute to condensate formation. This system therefore does not directly model the network of forces responsible for condensate formation by FUS alone, since this protein contains very few anionic side chains. However, our results may be relevant to FUS-mediated condensate formation in cells, which involves association of the cationic protein with anionic RNA.

## Results

### Experimental Design

A fragment comprising the C-terminal 154 residues of FUS (i.e., residues 372-526) was selected as the Arg-rich component; this polypeptide is designated “FUS-C” below (Figure 1). We hypothesized that the length of this fragment would enable phase separation upon combination with a short complementary peptide at low μM concentrations. FUS-C contains 24 Arg residues and is expected to have a net charge of approximately +13 near neutral pH. The 60 Gly residues in FUS-C should render this polypeptide largely disordered. In FUS itself, residues 422-453 constitute a zinc finger domain. For expression in *Escherichia coli*, the N-terminus of FUS-C was fused to maltose-binding protein (MBP) via a short linker that contained a tobacco etch virus (TEV) protease site (Figure 1A). This fusion strategy is commonly employed for heterologous expression of intrinsically disordered proteins because the MBP module promotes solubility and facilitates isolation and purification.^39,40^ FUS-C appeared to be fully soluble after TEV cleavage of 5 μM fusion protein at room temperature in 50 mM Tris, pH 7.5, containing 150 mM NaCl and 0.5 mM tris­(2-carboxyethyl)­phosphine (TCEP). The phosphine was included to prevent disulfide formation between Cys residues. Because no effort was made to remove zinc during purification of FUS-C, we assume that the zinc finger domain in our samples contained a bound zinc ion. We used TCEP as the reducing agent because this molecule is not expected to function as a zinc chelator.

**Figure 1:**
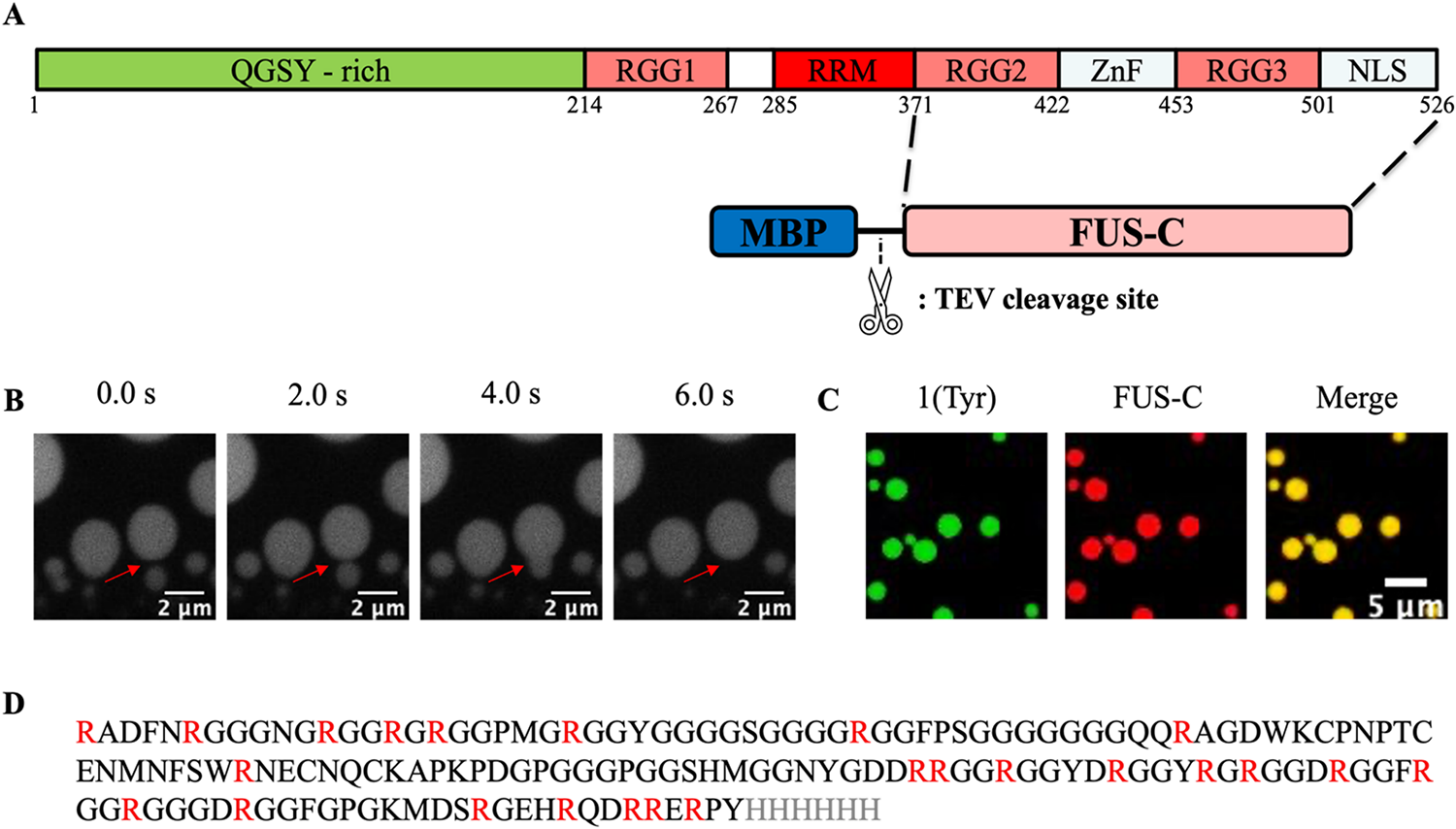
(A) Domain structure of full-length FUS (residues 1–526). The Arg-rich fragment used in this work is based on residues 372–526 (FUS-C). This fragment was expressed in a construct that included maltose-binding protein (MBP), with a TEV protease cleavage site in-between. (B) Time series of droplets formed by 50 μM **1­(Tyr)** (identified in Figure 2) and 5 μM FUS-C in 50 mM Tris, pH 7.5, containing 150 mM NaCl and 0.5 mM TCEP. The red arrows highlight droplet merger. Scale bar = 2 μm. (C) Colocalization of **1­(Tyr)** and FUS-C. The sample of **1­(Tyr)** included 1 mol % FITC-labeled **1­(Tyr)**, which was detected via fluorescence imaging at 488 nm, false colored green. The sample of FUS-C included 1 mol % TAMRA-labeled FUS-C, detected via fluorescence imaging at 561 nm, false colored red. The merged image is shown on the right. Scale bar = 5 μm. (D) Sequence of the FUS-C fragment. Arginine (R) residues are highlighted in red. The His_6_ tag is shown in gray. The sequence of full-length FUS is provided in the Supporting Information.

Preliminary studies with a peptide derived from the N-terminal Tyr-rich domain of FUS (corresponding to residues 33-66, a segment that includes seven Tyr residues and two acidic residues) did not lead to phase separation upon mixing with FUS-C. Peptides based on this portion of FUS tend to have low solubility because there are very few residues with charged side chains. We therefore designed 28-mer peptide **1­(Tyr)** as a partner for FUS-C (Figure 2A). In addition to 7 evenly spaced Tyr residues, **1­(Tyr)** contains 15 Gly residues, to ensure conformational disorder (Figure S6), and 6 Glu residues, to confer a net negative charge in the pH 7.5 buffer.

**Figure 2:**
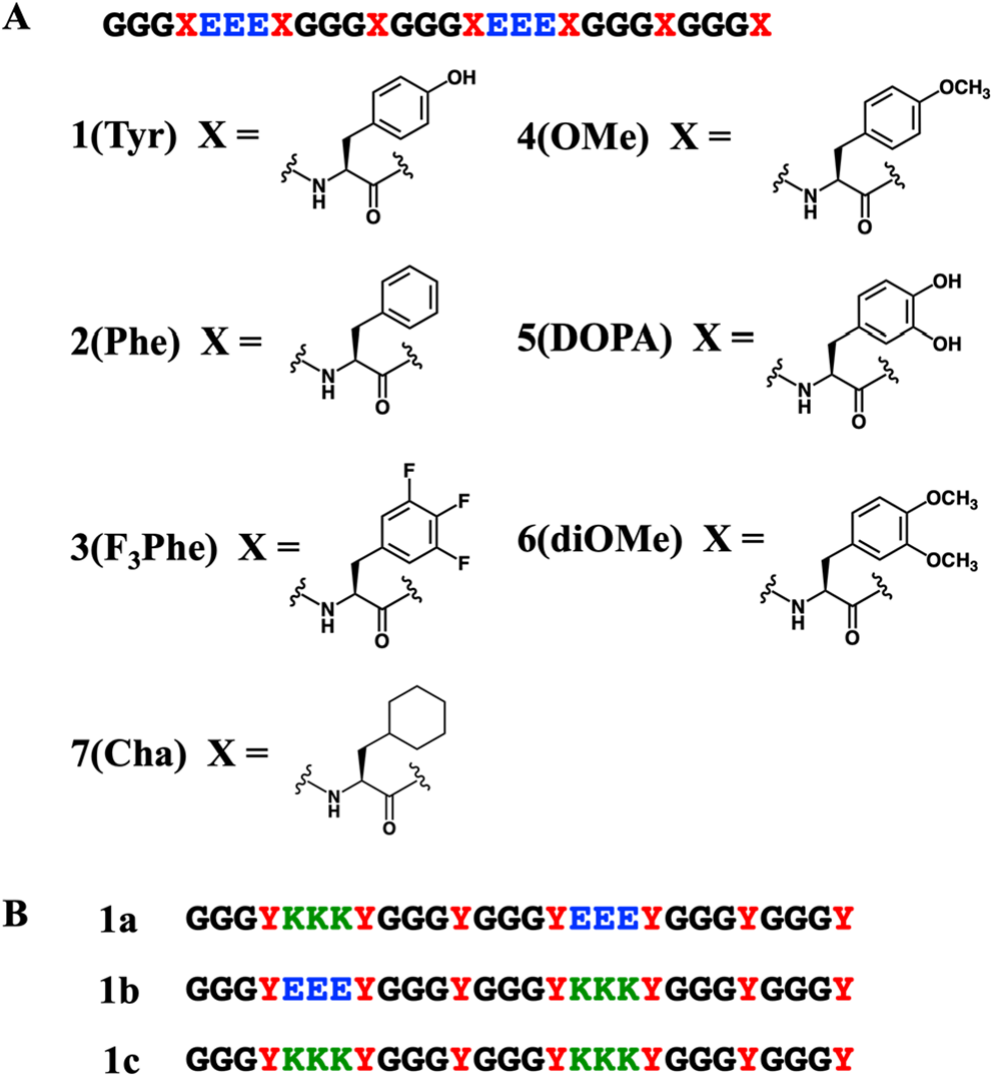
(A) Sequences of designed peptides with different residues at X sites. (B) Derivatives of **1­(Tyr)** with different charge states.

Combining 5 μM FUS-C and 50 μM **1­(Tyr)** at room temperature led to phase separation as detected via confocal fluorescence microscopy; the sample of **1­(Tyr)** contained 1 mol % of the peptide bearing an N-terminal fluorescein unit, to enable visualization of the droplets (Figure 1B). Condensed phase droplets were observed to fuse on a short time scale, which provided qualitative evidence of a liquid condensed phase. Fluorescence microscopy studies conducted with samples in which each component contained 1 mol % of a fluorescently labeled derivative, **1­(Tyr)** bearing an N-terminal fluorescein unit or FUS-C with an extra Cys inserted at the C-terminus for tetramethylrhodamine (TAMRA) attachment, established that both components were present in the condensates (Figure 1C).

To ask whether charge complementarity is necessary for condensate formation by FUS-C and **1­(Tyr)**, we examined analogues of **1­(Tyr)** in which three or all six of the Glu residues were replaced by Lys (Figure 2B). These peptides should have no net charge at pH 7.5 (**1a**–**b**) or a positive charge (**1c)**, in contrast to the expected negative charge of **1­(Tyr)**. Combining 50 μM **1a**, **1b** or **1c** with 5 μM FUS-C did not lead to phase separation. Thus, the net negative charge of **1­(Tyr)** is necessary for coassembly with cationic FUS-C to form the condensed phase, which means that the assembly is a complex coacervate.^14^ Charge complementarity should result in direct Coulombic attractions between **1­(Tyr)** and FUS-C as well as energetically favorable counterion release.^41–43^ This feature of our system represents a difference relative to condensate formation by pure FUS, which has very few acidic (anionic) residues.

To determine whether the negative charge of **1­(Tyr)** was sufficient for phase separation in the presence of FUS-C, we evaluated the analogue of **1­(Tyr)** in which all seven Tyr residues were replaced by Ala. No phase separation was observed when 5 μM FUS-C was mixed with 50 μM Ala analogue. The difference between the Tyr-rich and Ala-rich peptides shows that Coulombic attraction with FUS-C is not sufficient for phase separation under the conditions we employed. Thus, the Tyr side chains appear to play a crucial role in promoting condensate formation by the **1­(Tyr)**+ FUS-C pair, as is also true for condensate formation by FUS itself.^20^

To establish a point of comparison with studies of phase separation involving full-length FUS,^20,21^ we examined peptide **2­(Phe)**, which results from global Tyr→Phe replacement. Solutions containing 5 μM FUS-C and varying concentrations of either peptide in 50 mM Tris, pH 7.5, containing 150 mM NaCl and 0.5 mM TCEP, were evaluated. In an initial survey, samples containing 5, 10, 20, or 50 μM **1­(Tyr)** or **2­(Phe)**, including 1 mol % of the fluorescein-labeled derivative, were examined by confocal fluorescence microscopy (Figure 3A). For solutions containing **1­(Tyr)** and FUS-C, a few condensates were detected with 5 μM peptide, and condensates were abundant at 20 μM. In contrast, condensates were observed only at 50 μM **2­(Phe)**, the highest peptide concentration examined.

**Figure 3:**
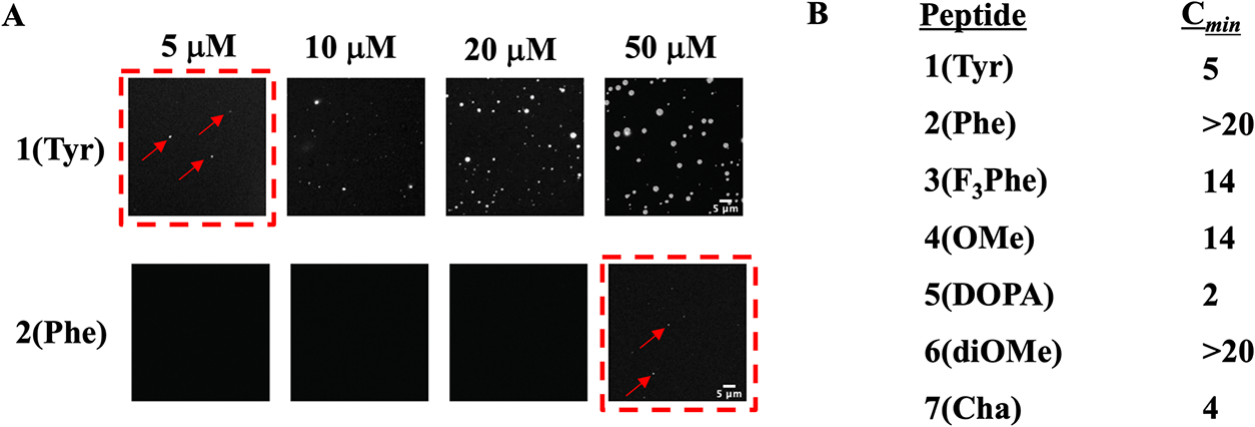
(A) Representative confocal images of droplets formed at different concentrations (μM) of **1­(Tyr)** or **2­(Phe)** with 5 μM FUS-C in 50 mM aqueous Tris buffer, pH 7.5, containing 150 mM NaCl and 0.5 mM TCEP. Scale bar = 5 μm. (B) The minimum peptide concentration (*C*_min_; μM) for each peptide at which droplets were visible by fluorescence microscopy when mixed with 5 μM FUS-C in 50 mM aqueous Tris buffer, pH 7.5, containing 150 mM NaCl and 0.5 mM TCEP. Data used to determine *C*_min_ values are shown in Figure S5. In each case, the peptide sample contained 1 mol % FITC-labeled peptide.

The dramatic difference between mixtures containing FUS-C and either **1­(Tyr)** or **2­(Phe)** that is evident in Figure 3A shows that the identity of the aromatic side chain plays a critical role in the molecular assembly required for condensed phase formation. These observations motivated experiments involving the set of 28-mers shown in Figure 2A to gain greater clarity on the role of the aromatic side chains in mediating phase separation in concert with FUS-C.

### Effect of Side Chain Identity on Phase Separation Propensity

For each of the peptides in Figure 2A, the propensity for phase separation upon mixing with 5 μM FUS-C was assessed in two ways. First, we identified the minimum peptide concentration (*C*_min_) at which droplets were visible by fluorescence microscopy 30 min after mixing (each peptide sample contained 1 mol % of the fluorescein-labeled derivative). The *C*_min_ values shown in Figure 3B are based on four independent measurements. We found that the precise concentration at which condensates appeared varied slightly among these measurements; the values shown are the lowest peptide concentration at which condensates were observed in at least three of the measurements. Representative micrographs are shown in Figure S5. These observations suggest that there is an inherent limit to the precision of such measurements, perhaps because of sensitivity to small variations in sample preparation.

Our second approach to evaluating phase separation propensity involved measuring total droplet area within a 3000 μm^2^ field of view (Figure 4). This analysis was carried out for samples containing 5, 10, 20, or 50 μM peptide along with 5 μM FUS-C. The area measurements were conducted with four independently prepared samples for each peptide + FUS-C pairing, with two independent measurements per sample. In each case, the microscopy images were obtained 30 min after the two components were mixed. In some cases, a very small droplet area was recorded at a concentration below *C*_min_ (e.g., for **3­(F**_**3**_ **Phe)** or **4­(OMe)**); these small droplet area values reflected the minor sample-to-sample variations in *C*_min_ noted above.

**Figure 4:**
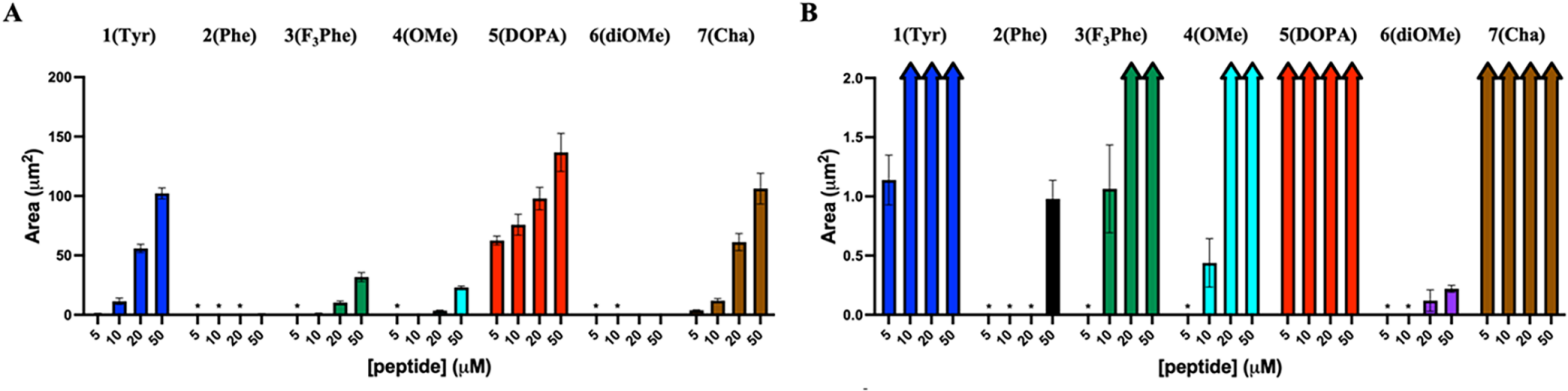
Area measurements of condensates observed in a 3000 μm^2^ field of view by confocal fluorescence microscopy. Phase separation conditions: 5 μM FUS-C was treated with the indicated peptide at different concentrations (each peptide sample contained 1 mol % FITC-labeled peptide) at room temperature in 50 mM aqueous Tris buffer, pH 7.5, containing 150 mM NaCl and 0.5 mM TCEP. Measurements made 30 min after mixing. *N* = 4 independent replicates (two measurements per replicate). Error bar, SEM. * indicates no droplets observed. (A) Data plotted with a vertical axis of 0 to 200 μm^2^. (B) The same data plotted with a vertical axis of 0 to 2 μm^2^; this scale highlights differences among samples with very few condensates. Most conditions result in condensate area measurements that exceed the vertical scale, as indicated by arrows at the top of the bars.

For **1­(Tyr)**, 5 μM was the lowest concentration at which droplets were reliably detected via microscopy under our conditions (Figure 3B), while the minimum concentration for **2­(Phe)** was >20 μM. Comparison of **1­(Tyr)** and **2­(Phe)** via droplet area at the four peptide concentrations reinforced the conclusion that these two peptides manifest very different propensities for phase separation upon mixing with FUS-C (Figure 4). Collectively, these results reinforced the conclusion that a net negative charge is not sufficient to enable the avid phase separation observed when **1­(Tyr)** is combined with FUS-C. The substantial decline in phase separation propensity resulting from global Tyr-to-Phe replacement in our system is consistent with findings of Wang et al., who reported that global replacement of Tyr with Phe in the N-terminal domain of FUS led to a decrease in phase separation propensity.^20^

The cation–π interaction arises from attraction between the positive charge and negative electrostatic potential of the aromatic ring (charge-quadrupole interaction).^30,31,44^ Dougherty et al. have estimated this attraction in the absence of solvent based on quantum mechanical calculations that map out the electrostatic potential of the aromatic unit.^37^ These calculations predict that phenol and benzene, representing the side chains of Tyr and Phe, respectively, are energetically similar as participants in cation–π interactions in the absence of solvent. However, subsequent calculations have suggested that phenol would form stronger cation–π interactions than benzene if the hydroxyl of phenol donated an H-bond to a water molecule.^45^ These computational results raise the possibility that in our system and in studies of full-length FUS, the decline in phase separation propensity resulting from global Tyr→Phe replacements results from replacing H-bond-augmented cation–π attractions involving the Tyr side chains with weaker, nonaugmented cation–π interactions of Phe side chains. However, we note that Tyr198 of the GABA_C_ receptor forms a cation–π interaction with the agonist γ-amino butyric acid (GABA), and Tyr198→Phe replacement causes only a small decline (∼4-fold) in GABA potency, which is consistent with the computational results in the absence of solvent.^37^ Subsequent affinity measurements with trimethyllysine-containing peptide ligands revealed only small changes (<2-fold change in K_D_) upon Tyr→Phe replacement in reader proteins.^38,46^ Collectively, these precedents are consistent with the expectation that the Tyr and Phe side chains form cation–π interactions of similar stability.

1,2,3-Trifluorobenzene, corresponding to the side chain of F_3_Phe, is predicted to be a much weaker participant in cation–π interactions relative to benzene or phenol in the absence of solvent.^37^ We therefore examined peptide **3­(F**_**3**_ **Phe)**, the analogue of **1­(Tyr)** in which all seven Tyr residues were replaced by F_3_Phe (Figure 2A). *C*_min_ for **3­(F**_**3**_ **Phe)** was 14 μM (Figure 3B), which indicates a higher propensity for phase separation with FUS-C for this peptide relative to **2­(Phe)** (*C*_min_ > 20 μM). Comparison of condensate area for **3­(F**_**3**_ **Phe)** vs **2­(Phe)** (Figure 4) supports the conclusion that **3­(F**_**3**_ **Phe)** has the higher propensity for phase separation in combination with FUS-C. In light of the gas phase calculations indicating that fluorinated benzene derivatives form weaker cation–π interactions than does benzene itself,^37^ it seems unlikely that cation–π interactions involving the aromatic side chains of **3­(F**_**3**_ **Phe)** drive phase separation in this case.

Further studies were conducted by combining Arg-rich FUS-C with **4­(OMe)**, **5­(DOPA)** and **6­(diOMe)**, the analogues of **1­(Tyr)** in which all seven Tyr residues were replaced by 4-methoxyphenylalanine, 3,4-dihydroxyphenylalanine (DOPA) or 3,4-dimethoxyphenylalanine, respectively. These peptides showed substantial differences in their abilities to support condensate formation with FUS-C. Eliminating the side chain H-bond donor, by replacing **1­(Tyr)** with **4­(OMe)**, caused the minimum concentration for condensate formation to rise from 5 to 14 μM (Figure 3B). At each peptide concentration used for condensate area measurements, the value was lower in solutions containing **4­(OMe)** + FUS-C relative to solutions containing **1­(Tyr)** + FUS-C. Previously reported quantum mechanical calculations suggest that there is little difference between the electrostatic potentials of the side chains of Tyr and 4-methoxyphenylalanine (i.e., phenol and anisole) in the absence of solvent.^37^ Moreover, replacing Tyr198 of the GABA_C_ receptor with 4-methoxyphenylalanine had little effect on GABA potency.^37^ Therefore, the decline in phase separation propensity with FUS-C manifested by **4­(OMe)** relative to **1­(Tyr)** seems inconsistent with the hypothesis that condensate formation in these systems is driven by attractions between cationic Arg side chains and aromatic side chain π systems. However, effects of H-bonding with water on the cation–π interactions of anisole have not previously been assessed computationally, and this factor, which is addressed below, might affect interpretation of the **1­(Tyr)** vs **4­(OMe)** difference.

Replacing **1­(Tyr)** with **5­(DOPA)** led to a substantial increase in phase separation propensity in combination with FUS-C. The minimum concentration for condensate formation declined to 2 μM for **5­(DOPA)** from 5 μM for **1­(Tyr)** (Figure 3B). This effect was evident also in condensate area measurements for solutions containing 5 μM peptide: the area value for **5­(DOPA)** was much higher than that for **1­(Tyr)** (Figure 4). These observations are consistent with the hypothesis that the H-bond donor properties of the aromatic side chain play a critical role in determining phase separation propensity upon peptide combination with FUS-C, and that the additional H-bond donor capacity of the DOPA side chain relative to the Tyr side chain underlies the increased phase separation propensity of **5­(DOPA)** relative to **1­(Tyr)**. Replacing **5­(DOPA)** with **6­(diOMe)** led to a dramatic decline in phase separation propensity: *C*_min_ was >20 μM for **6­(diOMe)**, and condensate area for **5­(DOPA)** was much larger than for **6­(diOMe)** at all concentrations.

Taken together, the observations for peptides **1­(Tyr)**, **2­(Phe)**, **4­(OMe)**, **5­(DOPA)** and **6­(diOMe)** are consistent with the hypothesis that aromatic ring substituents that serve as H-bond donors promote phase separation when the peptide is combined with FUS-C. However, given the interplay between cation–π interaction of phenol and water H-bonding to the phenol hydroxyl suggested by a previous computational study,^45^ isolating the contributions from these two factors is challenging. The analogue of **1­(Tyr)** containing global Tyr→Ser replacement did not cause detectable phase separation when combined at 50 μM with 5 μM FUS-C (Figure S7), which suggests that side chain H-bond donation alone is not sufficient for condensate formation in our system. However, the comparison between Ser and Tyr side chains as H-bond donors is imperfect, because the Tyr hydroxyl has a lower p*K*_a_ than the Ser hydroxyl, and the former is presumably a better H-bond donor.^47,48^

Observations with **3­(F**_**3**_ **Phe)** show that H-bond donors on the aromatic side chains are not required for promotion of phase separation upon mixing with FUS-C. To ask whether side chain hydrophobicity could promote phase separation for at least some peptide + FUS-C pairings, we compared reverse-phase high-pressure liquid chromatography (RP-HPLC) elution profiles for short peptides containing Tyr or one of the replacement residues (Figure 5). This comparison included cyclohexylalanine (Cha), which has a very hydrophobic and nonaromatic side chain. Tetrapeptides with the sequence Gly-Gly-Gly-Xxx were evaluated, where Xxx was the variable position. RP-HPLC elution profiles have commonly been used to compare hydrophobicity among peptides.^49–52^ The stationary phase for our comparison, silica modified with C18 alkyl groups, is highly hydrophobic, and the mobile phase, a gradient of water–acetonitrile mixtures, is hydrophilic. With a consistent elution protocol, longer retention should directly correlate with greater hydrophobicity.^49–52^

**Figure 5:**
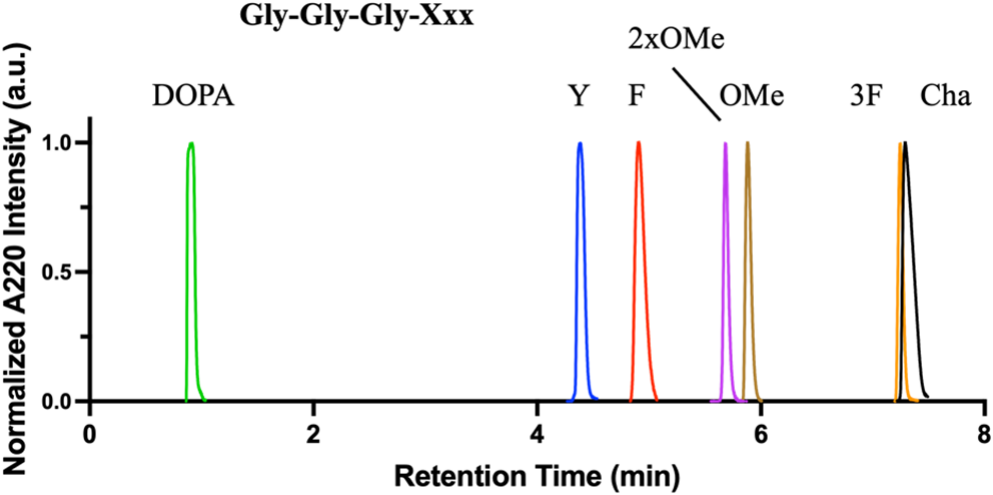
Reverse-phase high pressure liquid chromatography comparison of Gly-Gly-Gly-Xxx tetramers, where Xxx is one of the seven residues shown in Figure 2A. Elution gradient: 0%B 1 min, then 0–60%B 15 min; solvent A = 0.1% TFA in Milli-Q water; solvent B = 0.1% TFA in ACN.

The tetramer GGG-DOPA was the least strongly retained, which is consistent with the expectation that the pair of hydroxyl groups on the aromatic ring should render DOPA very hydrophilic. GGG-Tyr had the second-shortest elution time, and GGG-Phe was slightly more strongly retained, i.e., more hydrophobic. Both GGG-MeOPhe and GGG-DiMeOPhe were more hydrophobic (longer elution times) relative to the tetrapeptides bearing side chains with hydroxyl groups or GGG-Phe. The RP-HPLC comparison suggested that the side chain of F_3_Phe is similar to the side chain of Cha in terms of hydrophobicity.

The RP-HPLC results motivated us to evaluate **7­(Cha)** for the ability to promote phase separation in combination with FUS-C. This peptide displayed a strong propensity to support condensation formation. *C*_min_ was 4 μM for **7­(Cha)** (Figure 3B), comparable to *C*_min_ for **1­(Tyr)**. Condensate area at 5 μM peptide was larger for **7­(Cha)** relative to **1­(Tyr)** but comparable at higher peptide concentrations (Figure 4). The Cha side chain is obviously incapable of participating in cation–π interactions or donating H-bonds. Thus, the observations with **7­(Cha)** are consistent with the hypothesis that phase separation resulting from peptide + FUS-C mixing can be promoted by side chain hydrophobicity in our system. Based on the hydrophobicity of the F_3_Phe side chain implied by RP-HPLC results (Figure 5), we conclude that phase separation observed upon mixing **3­(F**_**3**_ **Phe)** with FUS-C is promoted by the hydrophobicity of the aromatic side chains rather than by cation–π interactions.

Phase separation data for peptide + FUS-C pairings in Figures 3 and 4 suggest that the overall phase separation propensity order among the 28-mer peptides is **5­(DOPA)** > **7­(Cha)** > **1­(Tyr)** > **3­(F**_**3**_ **Phe)** ∼ **4­(OMe)** > **2­(Phe)** ∼ **6­(diOMe)**. This order is quite different from the RP-HPLC elution order among the GGG-Xxx tetramers [Xxx = DOPA, Tyr, Phe, diOMe, OMe, F_3_Phe, Cha]. We interpret these data, collectively, to suggest that at least two different noncovalent interaction profiles can promote the attractions among peptide and FUS-C molecules necessary to create the network that scaffolds a condensed phase. One noncovalent interaction profile features H-bond donation by side chain groups, such as the hydroxyl groups of Tyr or DOPA, as a key factor. These data do not distinguish among the possibilities that the proposed H-bond donation enhances cation–π interactions, or that the H-bonds form between components of the condensate scaffold, or that both mechanisms are at work. In the other profile, hydrophobic interactions of side chains are a key factor.

Despite the similarity in side chain hydrophobicity for Cha and F_3_Phe suggested by the HPLC analysis, **7­(Cha)** has a higher phase separation propensity upon mixing with FUS-C relative to **3­(F**_**3**_ **Phe)**. The explanation for this difference is not clear. The higher phase separation propensity observed for **7­(Cha)** relative to **3­(F**_**3**_ **Phe)** provides further evidence against the significance of cation–π interactions in promoting condensation formation in our system, because the side chain of F_3_Phe can form weak cation–π interactions, while no cation–π interaction is possible with the side chain of Cha. Since neither Cha nor F_3_Phe is found in proteins, the hydrophobicity-based noncovalent interaction profile is probably not relevant to phase separation mediated by FUS-family proteins.

### Fluorescence Recovery after Photobleaching (FRAP) with Select Condensed Phases

FRAP studies^53^ were conducted to probe the physical properties of condensed phases formed by peptide + FUS-C pairs involving the three peptides with the largest phase separation propensities. Peptides **1­(Tyr)** and **5­(DOPA)** represented the H-bond-promoted mode of assembly, and **7­(Cha)** represented the hydrophobically promoted mode of assembly. For the FRAP studies, phase separation was induced by combining 5 μM FUS-C with 50 μM peptide in 50 mM Tris, pH 7.5, with 150 mM NaCl and 0.5 mM TCEP at room temperature. Samples containing 1 mol % fluorescein-labeled peptide were used to monitor peptide dynamics, and samples containing 1 mol % TAMRA-labeled FUS-C were used to monitor the dynamics of this protein fragment. In each case, the condensates were relatively small (diameter ≤ 1.5–2.5 μm), and the laser irradiation therefore bleached most of the droplet, even though these measurements focused on the largest droplets (Figure 6A). Fluorescence recovery in these cases resulted from peptide or FUS-C diffusion within the droplet and/or from peptide or FUS-C diffusion into the droplet from the surrounding dilute phase.

**Figure 6:**
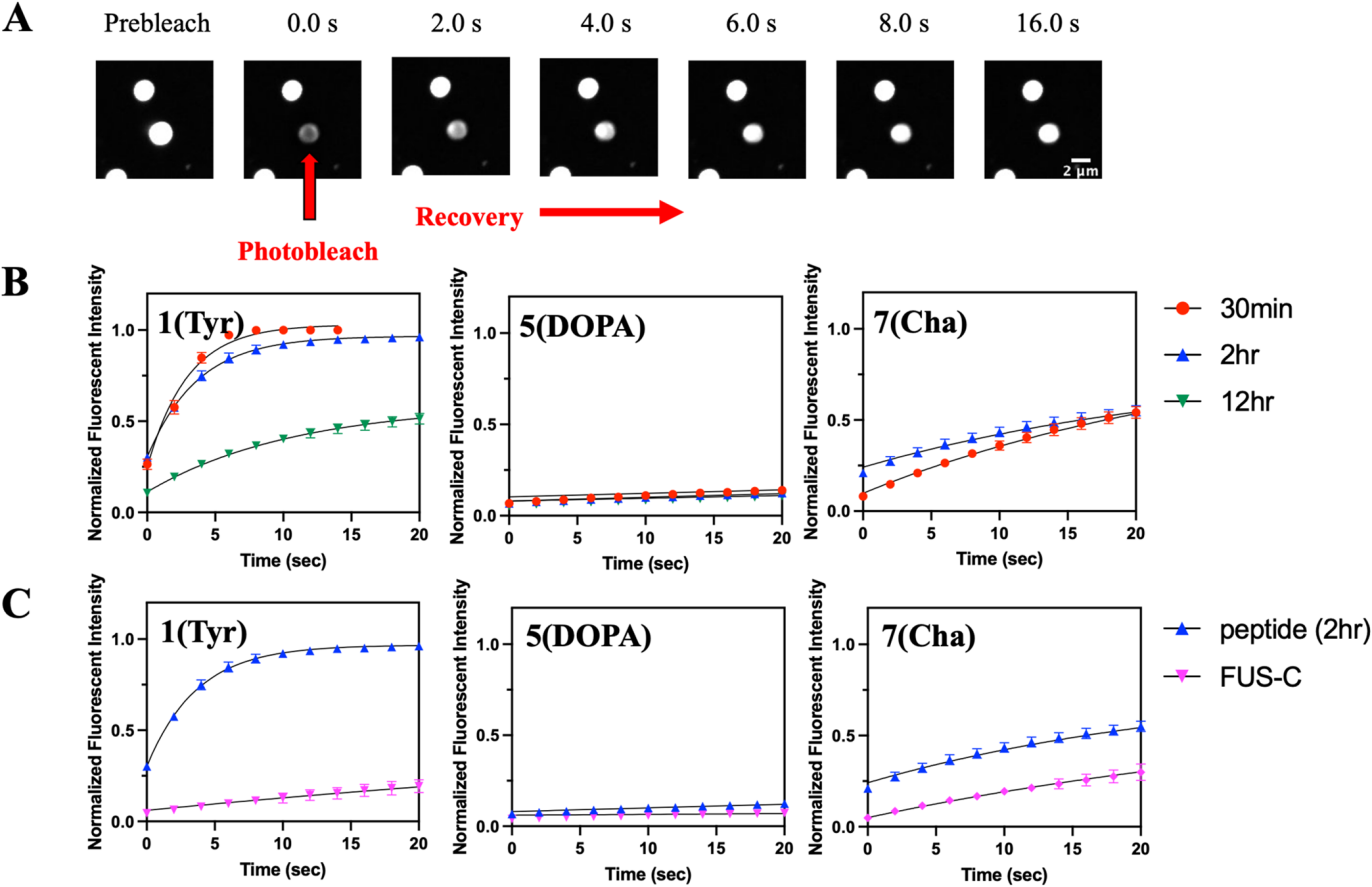
(A) Representative micrographs for a FRAP experiment on condensates formed by 50 μM **1­(Tyr)** (1 mol % FITC-labeled) and 5 μM FUS-C. At 0.0 s, a region of interest within the droplet is photobleached (dark). Scale bar = 2 μm. (B) FRAP comparison of 1**(Tyr)**, **5­(DOPA)** and **7­(Cha)** with 1 mol % FITC-labeled corresponding peptide at different time points: 30 min (red), 2 h (blue) and 12 h (green). (C) FRAP comparison between each of the three peptides (1 mol % FITC-labeled) and FUS-C (1 mol % TAMRA-labeled) at 2 h time points: peptide (blue) and FUS-C (magenta). The blue data for each peptide in parts (B) and (C) are the same.

For the three peptide + FUS-C pairs examined by FRAP, UV–vis absorption data indicated that the dilute phases contained substantial but different peptide concentrations (Figure 7): ∼1 μM **1­(Tyr)**, ∼13 μM **5­(DOPA)** or ∼4 μM for **7­(Cha)**. Thus, in each case, most of the peptide was located in the condensed phase. However, each dilute phase contained sufficient residual peptide to allow exchange with peptide in a condensate bleached by irradiation. We cannot explain the differences in dilute phase concentrations among the three peptides. Efforts to determine the concentration of FUS-C in the dilute phase were unsuccessful, which suggests that nearly all FUS-C was drawn into the condensed phases under our experimental conditions.

**Figure 7:**
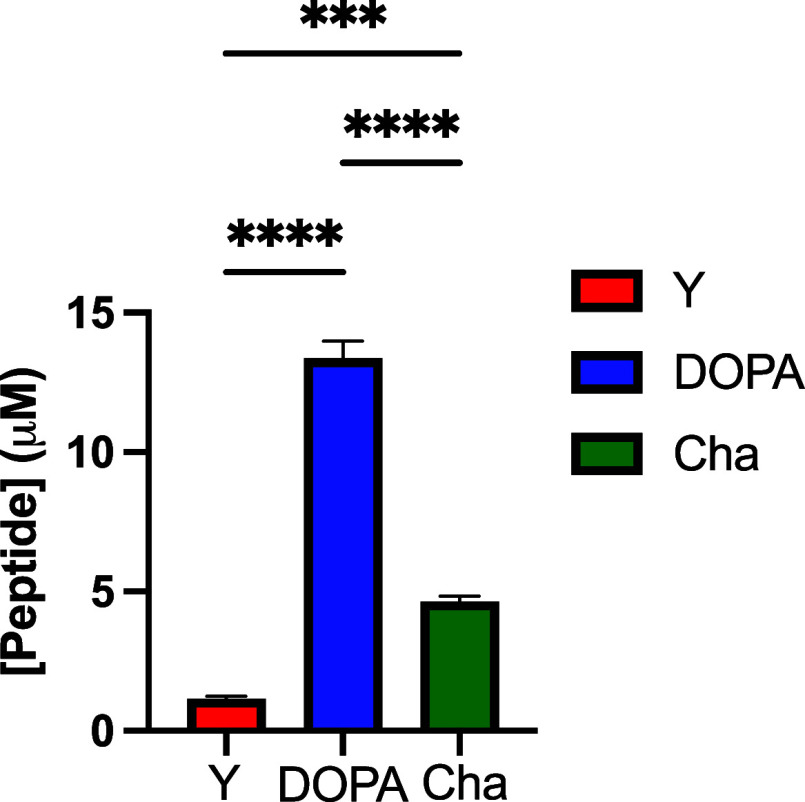
Concentration of peptide in the dilute phase after phase separation induced by mixing 5 μM FUS-C with 50 μM of corresponding peptide (10 mol % labeled): **1­(Tyr)**, **5 (DOPA)** or **7­(Cha)** in 50 mM Tris, pH 7.5, 150 mM NaCl, 0.5 mM TCEP. Samples were incubated at room temperature for 30 min. The coacervate sample was then centrifuged, and the supernatant was collected. The peptide concentration in the supernatant was determined by UV-spectroscopy. *N* = 4 independent replicates. Error bar, SEM. P values (*P* < 0.001 (***), *P* < 0.0001 (****)) were calculated by one way ANOVA.

In the condensed phase formed with **1­(Tyr)**, the peptide was very mobile (Figure 6B). At 0.5 h after mixing with FUS-C, condensates formed with **1­(Tyr)** fully regained the original fluorescence level with half-life ∼2 s after photobleaching; very similar behavior was observed 2 h after mixing. By 12 h after mixing, however, only ∼50% of the original fluorescence level was recovered, and the half-life for recovery was somewhat longer, ∼8 s. The diminished recovery maximum at 12 h suggests that there is a slow process by which peptide molecules become immobilized within the condensed phase and can no longer exchange with peptide molecules in the surrounding dilute phase, perhaps corresponding to gelation. Similar “aging” processes have been reported for condensed phases formed by FUS or FUS fragments.^54–56^ Since the concentration of **1­(Tyr)** in the dilute phase was lower than for **5­(DOPA)** or **7­(Cha)**, the rapid and complete fluorescence recovery observed over the first 2 h for **1­(Tyr)** suggests that the time scale and extent of fluorescence recovery for all three peptides was not limited by the amount of peptide in the dilute phase.

The FUS-C component appeared to be less dynamic than the peptide component in the condensed phase formed with **1­(Tyr)**. FRAP data collected at 2 h after mixing suggested a half-life of ∼100 s for recovery of the FUS-C component. The predicted extent of fluorescence recovery was ∼50%. These values did not change substantially at 12 h after mixing. Since the concentration of FUS-C in the dilute phase was apparently very low, the FRAP data may result from limitations in the amount of external FUS-C available to exchange with FUS-C bleached within the condensate. Alternatively, half of the FUS-C content in the condensates may be immobilized rapidly upon phase separation. The FRAP data could indicate that the slow process leading to immobilization of **1­(Tyr)** does not operate on FUS-C, or that this slow immobilization is not detected because of the rapid immobilization of a substantial fraction of the FUS-C.

FRAP measurements involving condensates formed with **5­(DOPA)** indicated substantially reduced mobility of the peptide and FUS-C components, relative to the condensed phase formed by mixing **1­(Tyr)** and FUS-C. For both **5­(DOPA)** and FUS-C, fluorescence recovery was very slow, whether the measurement was made at 0.5, 2 or 12 h after mixing. The extent of recovery could not be accurately gauged from the FRAP data. These observations suggest that the condensed phase formed by **5­(DOPA)** + FUS-C may be a gel rather than a liquid. Treatment of this condensed phase with 6 M guanidine HCl led to complete dissolution, which indicates that condensate formation with **5­(DOPA)** does not involve extensive oxidative covalent cross-linking of DOPA side chains (Figure S3).^57^ Condensates formed by **1­(Tyr)** + FUS-C and by **7­(Cha)** + FUS-C were also fully disrupted by 6 M guanidine HCl.

FRAP measurements with the condensate resulting from mixing peptide **7­(Cha)** and FUS-C revealed that the peptide component was dynamic at 0.5 and 2 h after mixing. The estimated half-life for fluorescence recovery was 15–20 s at both time points. The FUS-C component seemed to have a level of mobility similar to that of peptide **7­(Cha)**. FRAP data collected for FUS-C at 2 h after mixing suggested a half-life in the range 10–50 s for recovery. The predicted recovery was ∼50%. Measurement at 12 h, however, was impossible because the condensed phase droplets formed by **7­(Cha)** + FUS-C could no longer be observed via microscopy. The images suggested that after 12 h the droplets had merged and formed a thin, continuous layer across the surface of the microscope slide (Figure S4). This type of “wetting” was not observed for condensed phases formed when either of the hydroxyl-bearing peptides **1­(Tyr)** or **5­(DOPA)** was combined with FUS-C.

The surfaces of the glass slides used for these studies had been pretreated with bovine serum albumin (BSA) to minimize nonspecific adsorption. Our observation that the condensed phase formed by **7­(Cha)** + FUS-C slowly wets this surface is consistent with a recent report that another condensate formed with a hydrophobic protein pair wets BSA-treated glass surfaces.^58^

Condensate area comparisons (Figure 4) suggest that both **5­(DOPA)** and **7­(Cha)** have a higher propensity for coassembly with FUS-C relative to any of the other 28-mer peptides we evaluated. For both **5­(DOPA)** and **7­(Cha)**, condensed phase assemblies were very abundant at 5 μM peptide, while droplets were sparse for **1­(Tyr)** at 5 μM, and no droplets were observed for the other four peptides at this concentration. FRAP comparisons, however, indicate that the condensed phase promoted by H-bonding (**5­(DOPA)**) has material properties that differ from those of the condensed phase promoted by hydrophobicity (**7­(Cha)**). These differences support our general conclusion that two different modes of assembly underlie phase separation among the peptide + FUS-C pairs we have examined, one mode featuring H-bond donation by aromatic side chains and the other featuring hydrophobic interactions of side chains. Only the former mode could be relevant to phase separation mediated by FUS-family proteins.

### Computational Studies

We undertook quantum mechanical calculations to compare cation–π interactions of aromatic molecules representing the side chains in **1­(Tyr), 4­(OMe)**, and **6­(diOMe)**, i.e., phenol, anisole and 1,2-dimethoxybenzene. Our goal was to understand how interactions with water molecules might influence these cation–π interactions.

Sastry et al. conducted quantum mechanical calculations to evaluate the effect of H-bond donation by phenol to water on the strength of a cation–π interaction between a lithium cation and the aromatic ring of phenol.^45^ These studies involved the MP2 method in conjunction with aug-cc-pVDZ basis sets. The calculations were conducted with isolated molecules and ions (no solvation). The authors concluded that H-bond donation from phenol to water enhanced the cation–π interaction by 2–3 kcal/mol. Benzene would presumably not be susceptible to an H-bond-mediated augmentation of cation–π interaction stability. Therefore, these computational results raise the possibility that, in aqueous solution, a Tyr side chain forms stronger cation–π interactions than a Phe side chain, even though phenol and benzene are predicted to form cation–π interactions of comparable stability in isolation.^37^

Our approach took advantage of increases in availability of computational resources over the past two decades. All calculations were performed with Gaussian software using Møller–Plesset Perturbation Theory with single and double excitations (MP2). A correlation-consistent triple-ζ basis set, aug-cc-pVTZ, was employed. Geometry optimizations were performed on all structures. Harmonic vibrational frequency calculations confirmed all structures to be minima on the potential energy surface. In addition, we used a polarizable continuum model (PCM) with the coefficient of 78.3553, which corresponds to the dielectric constant of water, to model aqueous solution in order to enhance the relevance of these calculations to our experimental observations relative to calculations previously conducted on isolated species.^45^

The complexes evaluated computationally, structures A-G, are shown in Figure 8. In each case, we calculated the dissociation energy for the lithium cation from the aromatic π system. Gibbs free energies were obtained from the harmonic vibrational frequency calculations. Computed Gibbs free energies are presumably more appropriate than electronic energies for comparison with experimental outcomes, because electronic energies are based on molecules frozen in place, at 0 K, in a perfect vacuum. In contrast, Gibbs free energies include a thermal correction assuming the temperature of 298.1 K and account for entropy and zero-point vibrational energy, which provides a better basis for comparison with experimental outcomes. With the PCM for aqueous solution, the binding energies of the Li cation in structures A (0.31 kcal/mol), D (0.30 kcal/mol) and F (0.38 kcal/mol) were very similar (Table S2).

**Figure 8:**
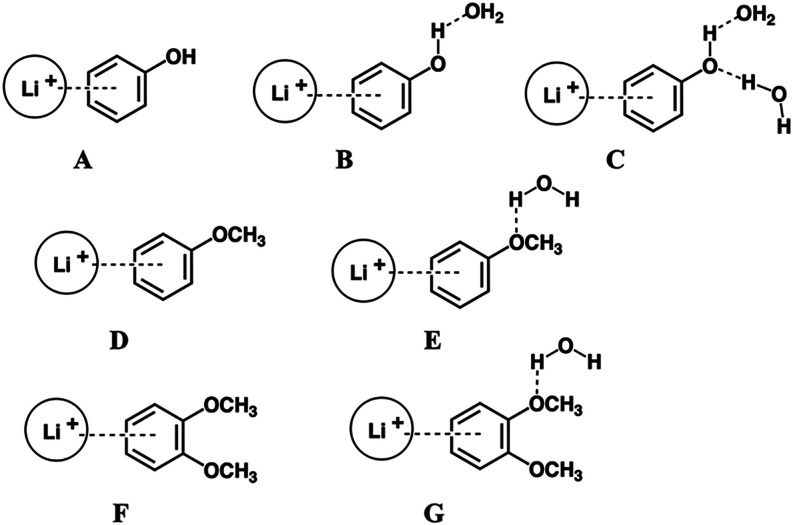
(A) Cation–π complexes between a lithium cation and selected aromatic molecules that represent the aromatic side chains in **1­(Tyr)**, **4­(OMe)** or **6­(diOMe)**. Complexes (B), (C), (E), and (G) include water molecules that form H-bonds with aromatic ring substituents. The stabilities of these complexes in a polarizable continuum model for aqueous solution were assessed via quantum mechanical calculations, as described in the text.

A PCM mimics the behavior of bulk solvent water, but this approach would not capture the covalent character of a hydrogen bond to an aromatic ring substituent. This effect was captured by the inclusion of explicit water molecules, as contemplated by Sastry et al.^45^ In order to account for specific H-bonds to aromatic ring substituents, we considered complexes B, C, E, and G, which include explicit water molecules in various configurations.

Complex A features a cation–π interaction between a lithium cation and phenol, while complex B features this cation–π interaction along with H-bond donation from the phenol hydroxyl to a water molecule. The computed difference in cation dissociation energies was very small, 0.15 kcal/mol in terms of ΔΔ*G* (Table 1). Although this small difference suggested a slightly stronger cation–π interaction in complex B, the ΔΔ*G* value was not significant given accuracy limits at the MP2 computational level.^59^ Thus, our computational comparison of complexes A and B suggested that within the PCM the inclusion of an explicit water molecule to act as a hydrogen bond acceptor does not substantially alter the binding affinity of the Li cation.

**Table 1:** Differences in Dissociation Energies (ΔΔ*E* or ΔΔ*G*) between Each Pair^a^

**structures**	**electronic energy** (kcal/mol)	**Gibbs free energy**(kcal/mol)
AB	0.08	–0.15
BC	0.06	0.24
DE	–0.12	–0.40
FG	–0.03	–0.21

a Calculated by subtracting the energy of the former structure from the energy of the latter.

In complex C, the hydroxyl of phenol donates an H-bond to one water molecule and accepts an H-bond from another water molecule. The difference in cation dissociation energies between complexes B and C was very small, 0.24 kcal/mol. This small difference suggested a slightly weaker cation–π interaction when the phenol oxygen serves as an H-bond acceptor, but this energy value is not significant in light of the accuracy limits of MP2 calculations.^59^ Therefore, this comparison strengthened the conclusion that the H-bonding status of the Tyr side chain hydroxyl group should have very little impact on a cation–π interaction involving this side chain.

Complex D has a cation–π interaction involving anisole, which represents the aromatic side chain in **4­(OMe)**. In complex E, this cation–π interaction is accompanied by donation of an H-bond from a water molecule to the oxygen of anisole. The difference in lithium ion dissociation energies for D vs E was 0.40 kcal/mol and again suggests that the inclusion of explicit water molecules does not significantly affect the results of the calculations. Thus, the effect of solvation, including H-bonding status of the methoxy group, on cation–π interactions of the 4-methoxyphenylalanine side chains in **4­(OMe)** is well described with the PCM and no explicit water molecule. A similar conclusion was reached with regard to the aromatic side chains in **6­(diOMe)** based on comparison of cation dissociation energies for complexes F and G.

Computational study of isolated cation–π interactions involving benzene, phenol or anisole suggested that all three should form cation–π interactions of similar strength.^37^ Our computational results support this conclusion. In aqueous solution as modeled by the PCM, the lithium-aromatic complexes A, D, and F exhibited very similar binding energies. Inclusion of explicit water molecules, as in complexes B, C, E, and G, did not cause a large divergence among binding energies. These computational results are consistent with the conclusion that differences in phase separation propensity among **1­(Tyr)**, **2­(Phe)**, **4­(OMe)** and **6­(diOMe)** when mixed with FUS-C are not primarily determined by differences in the cation–π interaction strengths of the aromatic side chains in these peptides.

## Discussion

Adoption of discrete tertiary structure by polypeptide chains and assembly of folded protein molecules into discrete quaternary structures are processes controlled by complex networks of noncovalent forces, involving contacts between atoms within the protein(s), protein–solvent interactions and protein-counterion interactions. The dynamic interactions among molecules of FUS or related proteins that lead to condensed phase formation presumably also involve a complex network of noncovalent forces. In such systems, it can be challenging to isolate the contributions of specific interactions because of cooperative effects and other complexities.^60–62^ Nevertheless, two studies published in 2018 concluded that cation–π interactions are essential for phase separation mediated by FUS and related proteins.^20,21^ Nearly every paper since 2018 that mentions biopolymer-mediated condensate formation of any type has cited cation–π interactions as a driving force.

The experiments described here were motivated by the goal of interrogating the cation–π interaction hypothesis in the context of condensate formation by Arg-rich and Tyr-rich components. Our approach was inspired by studies of Dougherty et al., Waters et al. and other groups in which specific interactions between a cation and an aromatic side chain were evaluated by modifying the side chain in ways that are not conventionally available via ribosomal synthesis.^30–38^ However, we acknowledge that interpretation of our results is constrained by the uncertainties inherent in studies that are not limited to a single, specific interaction within a folded protein.

Evolution has harnessed cation–π interactions to stabilize complexes between proteins and positively charged ligands, as established by experimental studies conducted over the past three decades.^30–38,63^ Close contacts between cationic and aromatic units in amino acid side chains observed in protein crystal structures suggest that cation–π interactions can stabilize native tertiary structure.^44,63^ Gallivan and Dougherty analyzed 593 protein crystal structures and concluded that on average there was one cation–π interaction for every 77 residues.^44^ This frequency is probably lower than the frequency of H-bonded contacts or nonpolar side chain contacts within tertiary structures.^62,64,65^ Thus, this analysis^44^ raises the possibility that although cation–π interactions play a significant role in higher-order protein structure, these interactions may not be a major driving force for protein folding. In contrast, both the hydrophobic effect and intramolecular H-bonding are believed to play key roles in determining specific protein tertiary structures,^62,64–67^ although these roles have been debated.^62^ (Tryptophan residues appear to be especially avid participants in cation–π interactions within proteins,^68–71^ although Trp residues are generally rare, and they are not prevalent in FUS family proteins.) This context, along with more recent studies of FUS family proteins,^20,21^ led us to wonder whether the cation–π interaction might be uniquely suited to formation of the dynamic intermolecular assemblies necessary to stabilize condensed liquid phases.^72^

Our studies involved a phase-separating system comprised of two components, a long Arg-rich protein fragment generated via heterologous expression and a short Tyr-rich peptide synthesized chemically [**1­(Tyr)**]. This strategy freed us from the compositional constraints imposed by ribosomal synthesis for the Tyr-rich component. However, the noncovalent interaction network that controls condensate formation induced by mixing FUS-C and **1­(Tyr)** differs from the noncovalent interaction network that controls condensate formation mediated by full-length FUS itself. Charge complementarity between FUS-C (a polycation) and the peptide (a polyanion) was necessary to achieve phase separation at low μM concentrations. **1­(Tyr)** contains 6 Glu among the 28 residues. In contrast, the entire Tyr-rich domain of FUS (residues 1-215) contains only five acidic residues. Our preliminary efforts to work with Tyr-rich fragments of <40 residues derived from the N-terminal domain of FUS were hampered by low peptide solubility, presumably a result of the lack of charged side chains. These difficulties led us to design **1­(Tyr)**.

It is possible that the charge complementarity required between FUS-C and anionic peptide **1­(Tyr)** leads to a condensed phase noncovalent network that is fundamentally different from the noncovalent network that underlies condensates formed by full-length FUS alone. However, two key observations suggest that qualitative conclusions drawn from our model system can be extrapolated to phase separation mediated by FUS family proteins. First, the analogue of **1­(Tyr)** with global Tyr → Ala replacement does not support phase separation with FUS-C under our conditions. This observation shows that Coulombic complementarity between FUS-C and the 28-mer peptide is not sufficient for condensate formation, and that the Tyr side chains are essential. Second, global Tyr → Phe replacement [**1­(Tyr)** → **2­(Phe)**] causes a profound decline in phase separation propensity, which parallels the impact of global Tyr → Phe replacement in FUS.^20^ Thus, in both systems, the nature of the aromatic side chain is critical.

Results obtained in our designed system are not consistent with the hypothesis that cation–π interactions are a major driver of phase separation induced by mixing FUS-C and **1­(Tyr)**. This conclusion is evident not only from the decline in phase separation propensity upon global Tyr → Phe replacement, but also from the significant decline in phase separation propensity upon replacing Tyr with O-methyl-tyrosine [**4­(OMe)**]. In the absence of solvent, ab initio calculations suggest that the methoxyphenyl side chain has a slightly larger cation-binding energy than does the phenol side chain in the absence of solvent.^37^ Our computational results, which attempt to account for solvent effects, suggest that the Tyr and O-methyl-tyrosine side chains should form cation–π interactions of comparable stability in aqueous solution.

Our observation that replacing Tyr with DOPA [**1­(Tyr)** → **5­(DOPA)**] substantially enhances phase separation propensity and results in a more ordered condensate suggests that H-bond donation by the Tyr and DOPA side chains plays a critical role in promoting phase separation in our system. This conclusion is consistent with analysis of Tyr → Phe mutations in folded proteins, which suggests that intramolecular H-bond donation by the Tyr hydroxyl can stabilize native tertiary structure.^64^ Support for the proposed role of side chain H-bond donation in our system is provided by the substantial decline in LLPS propensity observed for **4­(OMe)** relative to **1­(Tyr**) and for **6­(diOMe)** relative to **5­(DOPA)**. Each of these comparisons involves removal of H-bond donors, apparently without diminishing the ability of the aromatic ring to engage in cation–π interactions.

Our data indicate that placing one methoxy group on the side chain aromatic ring [**2­(Phe)** → **4­(OMe)**] causes an increase in phase separation propensity in combination with FUS-C, but placing a second methoxy group on the aromatic ring [**4­(OMe)** → **6­(diOMe)**] causes a decrease in phase separation with FUS-C (Figure 4). An explanation for this trend is not clear to us. HPLC data (Figure 5) suggest that the side chain of O-methyl-tyrosine is slightly more hydrophobic than the side chain of di-O-methyl-DOPA, but this difference seems too small to explain a substantial variation in phase separation propensity.

The ability of the Tyr side chain to serve as an H-bond donor has received relatively little prior attention in the context of polypeptide-mediated phase separation. The most relevant studies, to our knowledge, have emerged from work inspired by mussel adhesive proteins.^73^ Mussels anchor themselves to solid substrates via “foot proteins”, many of which contain abundant DOPA residues. These proteins undergo phase separation as they are being secreted; exposure to seawater triggers solidification of the condensed phase. Some model studies have suggested that cation–π interactions involving DOPA side chains and Lys side chains, which are also abundant in mussel foot proteins, contribute to the self-adhesion of these proteins.^74–78^

Recent studies of salt-triggered phase separation by a synthetic peptide rich in DOPA and Lys have raised the possibility that H-bonding rather than cation–π interactions drives condensate formation in this system.^79^ This peptide, designed based on a mussel foot protein, contained only DOPA, Lys and Gly and supported phase separation at high concentrations of peptide and salt. The analogous peptide with global DOPA → Tyr replacement did not undergo phase separation under the same conditions. Molecular dynamics simulations suggested that network formation by the DOPA-rich peptide was stabilized by intermolecular H-bonding between two DOPA side chains or between a DOPA side chain and the backbone of a Gly residue. Related studies on the mussel adhesive protein Pvfp-5β (83 residues) compared the version rich in Tyr with the analogue resulting from global Tyr → DOPA replacement.^80^ Phase separation occurred for solutions containing high concentrations of the DOPA version and salt; in contrast, the Tyr version formed solid aggregates under these conditions. Although Pvfp-5β contains eight Lys residues, a combination of ^13^C NMR measurements and quantum calculations led to the conclusion that cation–π interactions were not a driving force for assembly of either the Tyr or DOPA versions of this protein. Instead, the associations underlying condensate formation by the DOPA version were attributed to H-bonding and π-π interactions of DOPA side chains.

Testing the hypothesis that cation–π interactions promote phase separation observed for FUS-C in combination with **1­(Tyr)** led us to examine **3­(F**_**3**_ **Phe)**. We found that **3­(F**_**3**_ **Phe)** had a much greater condensate formation propensity relative to **2­(Phe)** (Figure 4), which is contrary to the trend that would be predicted if cation–π interactions were energetically important for phase separation in this system. Hydrophobicity assessment via RP-HPLC indicated that F_3_Phe is considerably more hydrophobic than Phe (Figure 5). This finding is consistent with an earlier report that pentafluorophenylalanine is more hydrophobic than phenylalanine.^81^ Enhancement of hydrophibicity as a result of H→F replacements is not unique to aromatic side chains: fluorine-bearing alkyl side chains are more hydrophobic than their natural alkyl counterparts.^82–84^

We have established an experimental system to explore the hypothesis that cation–π interactions play a major role in promoting phase separation mediated by Arg-rich and Tyr-rich polypeptides. This study was inspired by extensive analysis of phase separation mediated by FUS and related proteins, which contain Arg-rich and Tyr-rich domains.^20,21,30^ Use of different polypeptides to provide the cationic and aromatic side chains, with the latter component short enough to be accessible via chemical synthesis, has enabled us to build upon earlier experimental designs that identified energetic contributions of isolated cation–π attractions to protein–ligand interactions.^30–38^ Our results raise the possibility that cation–π interactions may not play a significant role in phase separation mediated by a designed 28-residue Tyr-rich peptide and an Arg-rich polypeptide corresponding to the last 154 residues of FUS. Instead, our data suggest that H-bond donation by the Tyr side chain hydroxyl group is an energetically important factor in the phase-separation process.

We speculate that the conclusions drawn from our studies are relevant to membraneless organelle formation within cells mediated by FUS and related proteins. However, it is possible that the requirement for anionic charge on the peptide in our system leads to a network of noncovalent forces in our condensates that is fundamentally different from the noncovalent network in a condensate formed by FUS or a related protein. Understanding the noncovalent interactions that stabilize the dynamic macromolecular assemblies within membraneless organelles will provide a foundation for efforts directed toward targeting these assemblies for biomedical applications.^6,7,18,54,55,85,86^

## Supplementary Material





## Notes

Materials (Section I), method descriptions (Section II), additional figures and tables (Section III), and characterization data for all synthesized peptides (Section IV) (PDF)
Output files and images for computational analysis (Section V) (ZIP)
