# Supplementary material for: Interplay of Noncovalent Interactions in Phase Separation Mediated by Tyrosine-Rich and Arginine-Rich Polypeptides
Source: J Am Chem Soc. 2026 Jun 17;148(25):26585–98. doi: 10.1021/jacs.6c06972 (PMC13339138; doi:10.1021/jacs.6c06972)
Supplement: Supplementary file 1 [file ja6c06972_si_001.pdf]

Supporting Information for

Interplay of Noncovalent Interactions in Phase Separation Mediated by Tyrosine-Rich and Arginine-Rich Polypeptides

Ruiwen Xu, [1] Rui Wang,[1] Cindy Qiu,[2] Jiani Niu,[1] Desiree M. Bates,[1,*] Nicholas L. Abbott,[2,*] Samuel H. Gellman[1,*]

[1]Department of Chemistry, University of Wisconsin-Madison, Madison, Wisconsin 53706, USA.

[2]Robert F. Smith School of Chemical and Biomolecular Engineering, Cornell University, New York 14853, USA

*Correspondence should be addressed to S.H.G. (gellman@chem.wisc.edu), N.L.A. (nla@cornell.edu) or D.M.B. (dmbates@chem.wisc.edu)

I. Materials and Instrumentations

1) Material Sources

Fmoc-protected amino acids, ethyl (hydroxyimino) cyanoacetate (Oxyma), N,N'-diisopropylcarbodiimide (DIC),and hexafluorophosphate azabenzotriazole tetramethyl uronium (HATU) were purchased from Chem-Impex international. Fluorescein isothiocyanate was purchased from Thermo Scientific. Diisopropylethylamine (DIEA), N,N-dimethylformamide (DMF), dichloromethane (DCM), piperidine, trifluoroacetic acid (TFA), triisopropylsilane (TIPS), thioanisole, acetonitrile, guanidinium chloride (GdmCl), sodium chloride (NaCl), Tris base, Dulbecco's PBS, RNA from torula yeast and BSA (7.5%) were purchased from Millipore Sigma. SPPS reaction vessel syringes and caps were purchased from Torviq. Rink Amide Resin (Low Loading) was purchased from CEM. Four-chamber glass bottom dishes were purchased from Cellvis.

2) Instrumentations and Instrumentation Acknowledgments

Microscopic data were obtained at the University of Wisconsin – Madison Optical Imaging Core. The purchase of Nikon AXR Confocal Microscope was funded by NIH 1S10O34394-01.

The purchase of the Bruker ultraflex™ III mass spectrometer equipped with a SmartBeam™ laser was partially funded by NIH NCRR award 1S10RR024601-1.

The purchase of JASCO J-1500 CD spectrometer was funded by NIH R01 GM061238.

The Waters UPC2-MS instrument was supported by NIH 1S10OD036302-01, and we thank the UW-Madison Department of Chemistry SynCat Center for assisting with its operation.

II. Methods

1) Peptide synthesis, purification, and characterization

All peptides were prepared via CEM MARS6 microwave-assisted solid phase peptide synthesis. 50 µmole Rink Amide ProTide resin (LL) was added to a Torviq solid-phase peptide synthesis vessel with a stir bar. Resin was swelled in 1:1 v/v DMF:DCM for 10 minutes before the synthesis. Fmoc amino acids (4 eq., 0.1 M) were activated with ethyl cyano(hydroxyimino)acetate (Oxyma, 8 eq.) and N,N'-diisopropylcarbodiimide (DIC, 4 eq.), and this solution was added to the reaction vessel. Regular coupling cycles were done at 70°C for 4 minutes. Double extended coupling at 70°C for 12 min was performed for all noncanonical amino acids (TyrOMe, DOPA, F_3Phe, diOMe, Cha). Then the resin was washed 5 times with DMF. The Fmoc protecting group was removed by reacting with 20% v/v piperidine in DMF at 80°C for 2 minutes. For peptides bearing a fluorescein unit, a Fmoc-protected β-alanine residue was added to the N-terminus. After the Fmoc group was removed, the resin-bound peptide was mixed with fluorescein isothiocyanate (2 eq.) and DIEA (8 eq.) in DMF for 6 hours at room temperature in dark.

After all residues had been added, the resin was mixed with the cleavage cocktail (*8 mL per 50 μmole*), which is 94 % (v/v) TFA, 2.5 % (v/v) H_2O, 2.5 % (v/v) thioanisole, and 1% (v/v) TIPS, at room temperature for 3 hours. The solution containing the crude cleaved peptide was drained from the reaction vessel into a 50-mL falcon tube. Excess TFA was removed under a stream of nitrogen, and about 35 mL of cold diethyl ether was added to precipitate the crude peptide. The crude peptide pellet was isolated by filtration, washed with cold diethyl ether twice, and then dried under nitrogen. The crude peptide was dissolved in DMSO (*2 mL per 50 μmole*) for HPLC purification. Dissolved peptide was filtered through a 0.22-micron Nylon filter and purified on an Agilent preparative HPLC system with a C18 CSH Prep column (5 μm, 19x250 mm, solvent A = H_2O + 10 mM NH_4OH, solvent B = acetonitrile + 10 mM NH_4OH, flow rate = 13 mL/min). Peptide masses were determined using MALDI-TOF-MS and ESI-MS, and purity was characterized by analytical UPLC (detection at 220 nm). HPLC fractions containing pure peptide were combined, and this solution was lyophilized (freeze-dried) for long-term storage. The lyophilized powder was dissolved in H_2O for all experiments.

2) General protocol for protein expression and purification

A plasmid containing TEV protease gene pDZ2087 was a gift from David Waugh (Addgene plasmid # 92414). TEV protease was expressed and purified as previous reported. Purified TEV protease was aliquoted and stored at -20 °C for future use.[1]

The DNA fragments encoding two constructs: the wildtype FUS C-terminus (372-526) and FUS C-terminus (372-526) with a Cys inserted between R524 and P525, with a TEV protease cleavage site at the N-terminus (sequence: ENLYFQG) and an additional 6 histidine residues at the C-terminus (sequence: HHHHHH), were ordered from Twist Bioscience, and cloned into AvaI and EcoRI sites of vector pMAL-c5x (this vector contains the sequence for maltose binding protein). The resulting plasmids were transformed into E. coli strain BL21(DE3), and cells were grown at 37 °C in 2XYT with 100 μg/L ampicillin. At OD600 = 0.4, overexpression was induced using 1 mM IPTG at 18 °C for 12 hr. After cells were harvested through the centrifugation, cells were resuspended and lysed in 50 mM Tris buffer pH 7.5, 1000 mM NaCl, 0.5 mM TCEP, 1 mM $MgCl_2$, 200 μg/mL lysozyme, 10 μg/mL DNase I, and cOmplete protease inhibitor using sonication. The protein was purified over a HisPrep FF column, and eluted with 50 mM Tris buffer pH 7.5, 1000 mM NaCl, 0.5 mM TCEP and 500 mM imidazole. To remove the excess imidazole, the protein solution was dialyzed against 10 volumes of buffer containing 50 mM Tris buffer pH 7.5, 150 mM NaCl, 0.5 mM TCEP. The protein was further purified using a Superdex 200 pg size exclusion column. The purified FUS C-terminus (372-526) with Cys inserted was further treated with 2 equivalents of maleimide-TAMRA and purified with another size-exclusion column to generate TMR-FUS.

3) Circular Dichroism (CD)

CD experiments were conducted at 25 °C on a JASCO J-1500 CD spectrometer. Peptide samples were diluted to 50 μM in PBS, pH 7.4, and then transferred to a 1-mm quartz cuvette. The CD spectrum was measured from 260 to 200 nm with 0.1 nm intervals at 4 second digital integration time (D.I.T) at 100 nm/min scanning speed. The peptide concentrations were carefully remeasured

for CD experiments, and the accurate values were used for molar ellipticity calculations. The data are presented as mean residue ellipticity in (deg•cm^2•M^{-1}) averaged for ten spectral scans.

4) Confocal Microscopy

Glass-bottom confocal dishes were passivated with 1% BSA in PBS and washed three times with MilliQ water. Peptide solutions were prepared by mixing 99 mol% unlabeled peptide and 1 mol% fluorophore-labeled peptide in 50 mM Tris buffer pH 7.5, 150 mM NaCl. The protein solution MBP-FUS-C was treated with 5 mol% TEV protease in 50 mM Tris buffer pH 7.5, 150 mM NaCl, 0.5 mM TCEP at room temperature for 6 hours. The cleavage product was then mixed with the peptide solution. Peptide and protein solutions were mixed (3 times pipetting) in the confocal dish, and the solution was incubated at room temperature. We found reproducibility of results depended on strict consistency in the mixing/pipetting protocol. Images were acquired on a Nikon AXR confocal microscope equipped with a 60x oil immersed objective at different timepoints. A 488-nm laser was used to excite the Fl-peptide samples; a 561-nm laser was used to excite the TMR-FUS samples. Images were processed in ImageJ.[2]

5) Fluorescence Recovery After Photobleaching (FRAP)

Peptide + FUS-C solutions containing 1 mol% labeled peptide or 1 mol% labeled protein were prepared as in section 4 above. The pre-and post-bleaching images were acquired on a Nikon AXR with the low laser power setting. A region of interest (ROI) with diameter = 1 μm was selected at the center of a relatively large droplet. Bleaching was performed with 100% 488-nm laser power for 8 seconds to reach >80% fluorescence reduction. The fluorescence recovery was monitored with the smallest time interval (2 sec).[3,4]

6) Determination of peptide concentration in the dilute phase

Phase separation samples were prepared by combining 50 μM peptide (10 mol% fluorescein-labeled) and 5 μM FUS-C in 50 mM Tris pH 7.5, 150 mM NaCl, 0.5 mM TCEP. The total volume (V_0) was 0.4 mL. These samples were incubated for 30 minutes and then subjected to centrifugation (13,000 g, 30 min) to force the condensed phase to the bottom of the tube. A small aliquot (~ 10 μL) of the supernatant (assumed to be pure dilute phase) was then carefully removed from the top of the sample. Absorbance at 488 nm, which arises from the fluorescein moiety, was recorded for this sample via UV-Vis spectroscopy. Based on an extinction coefficient of 75,000 M^{-1}cm,[5] the absorbance at 488 nm was used to calculate the concentration of the peptide in the dilute phase.

7) Measurement of droplet area

Phase separation samples were prepared by mixing appropriate amounts of peptide (1 mol% fluorescein-labeled) and 5 μM FUS-C in 50 mM Tris pH 7.5, 150 mM NaCl, 0.5 mM TCEP. The mixture was transferred to the confocal plate (1% BSA coated). The sample was allowed to stand

for 30 min to allow droplets to sediment. Images were taken afterwards. N = 4 independent replicates (two measurements per replicate). Images were processed using ImageJ.[2]

8) Computational Methods

All structures were optimized with MP2 in conjunction with aug-cc-pVTZ basis set. The stationary points were determined to be a minimum via harmonic vibrational frequency calculations. For all calculations, a Polarizable Continuum Model (PCM) was used, with a dielectric constant for water. The dissociation energy of removing the Li atom was calculated by electronic and Gibbs Free Energies. The dissociation energies of the difference species, with and without a water present, are reported ($\Delta\Delta E$). All calculations were performed using the Gaussian 16 program package and WebMO software.[6,7] Output files and images for computational analysis are included in the SI.

III. Supporting Figures and Tables

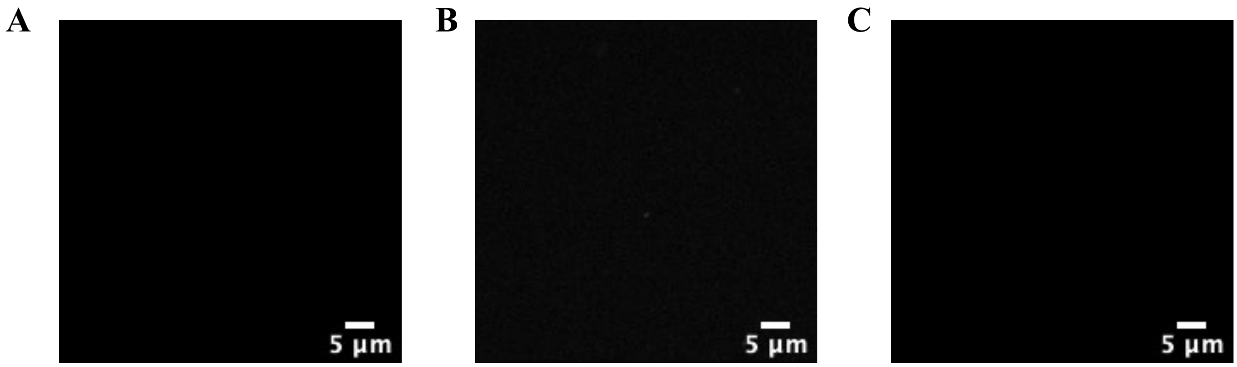

Figure S1. Representative confocal images of 50 µM **1a** (**A**), **1b** (**B**) or **1c** (**C**) when mixed with 5 µM FUS-C (1 mol% TAMRA-FUS-C). Scale bar = 5 µm. None of these three peptides induces phase separation under these conditions after 30 min of incubation. Buffer condition: 50 mM Tris, pH 7.5, 150 mM NaCl and 0.5 mM TCEP.

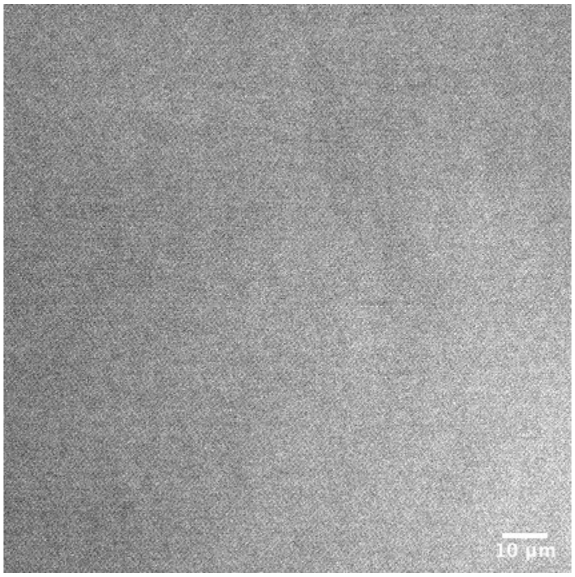

Figure S2. Representative DIC image of 5 µM maltose binding protein with 50 µM **1(Tyr)**. Scale bar = 10 µm. No coacervates or aggregates were observed after 30 min of incubation. Solutions used for phase separation studies contained FUS-C along with the maltose binding protein (which had been detached by TEV protease treatment). We conclude that maltose binding protein does not participate in the phase separation behavior observed for peptide + FUS-C pairings. Buffer condition: 50 mM Tris, pH 7.5, 150 mM NaCl and 0.5 mM TCEP.

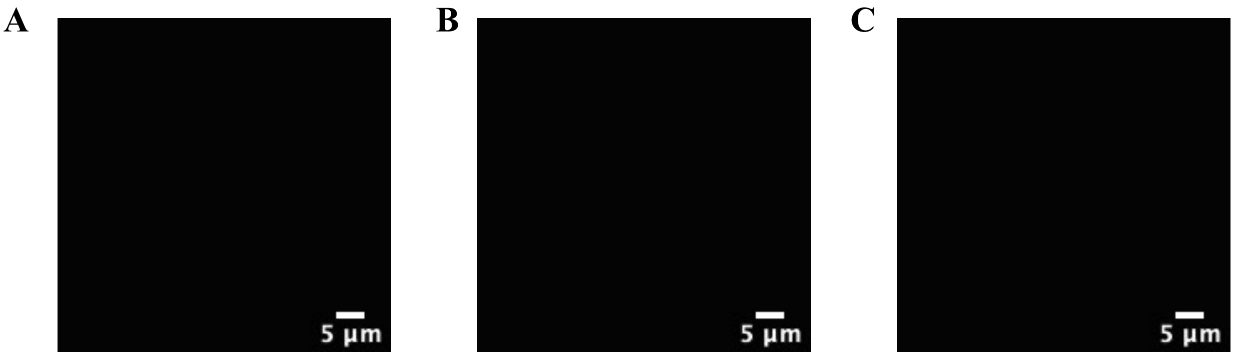

Figure S3. Representative confocal images after 6 M guanidine HCl treatment of droplets formed between 5 μM FUS-C and 50 μM **1(Tyr)** (1 mol% fluorescein-**1(Tyr)**, **A**), **5(DOPA)** (1 mol% fluorescein-**5(DOPA)**, **B**), or **7(Cha)** (1 mol% fluorescein-**7(Cha)**, **C**). Scale bar = 5 μm. No droplets were observed after the treatment with guanidine HCl following 30 min of incubation. For the sample containing **5(DOPA)**, this result indicates a lack of large-scale covalent crosslinking. Buffer condition: 50 mM Tris, pH 7.5, 150 mM NaCl and 0.5 mM TCEP.

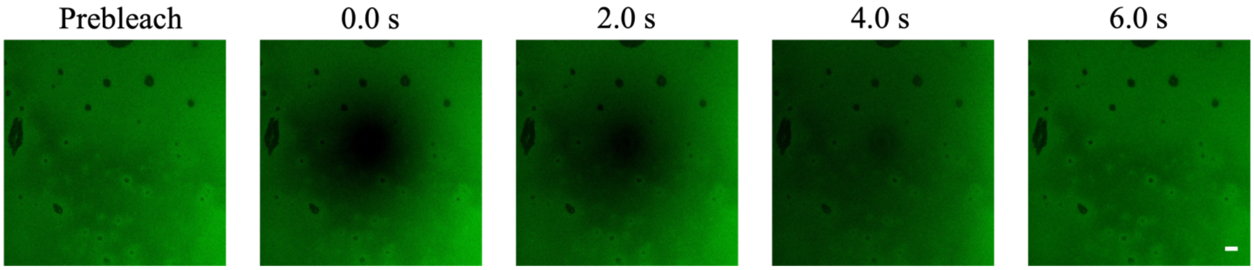

Figure S4. Representative confocal micrographs of coacervates formed with 5 μM FUS-C and 50 μM **7(Cha)** (1 mol% fluorescein-**7(Cha)**) at the 12-hour timepoint. No clear boundary for coacervate droplets was observed. Instead, the condensed phase appeared to have spread across the surface. These images show results of a FRAP experiment. Laser irradiation bleaches a region of the condensed phase covering the surface, and recovery of fluorescence is observed. Scale bar = 2 μm. Buffer condition: 50 mM Tris, pH 7.5, 150 mM NaCl and 0.5 mM TCEP.

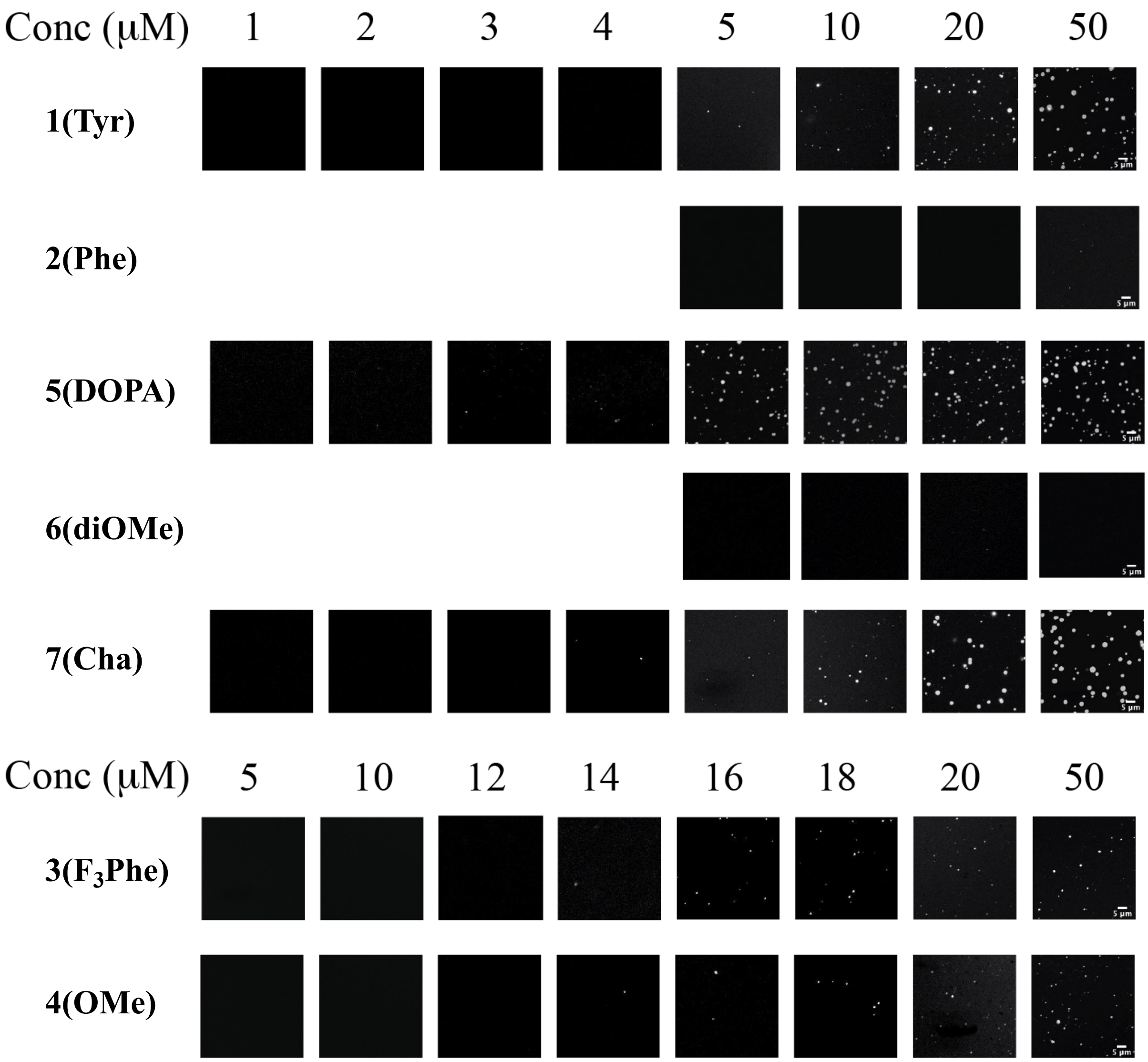

Figure S5. Representative confocal micrographs of coacervates at different concentrations of corresponding peptides with 5 µM FUS-C, 30 min after mixing. These data were used to determine the C_{min} values shown in Fig. 3B. Scale bar = 5 µm. Buffer condition: 50 mM Tris, pH 7.5, 150 mM NaCl and 0.5 mM TCEP.

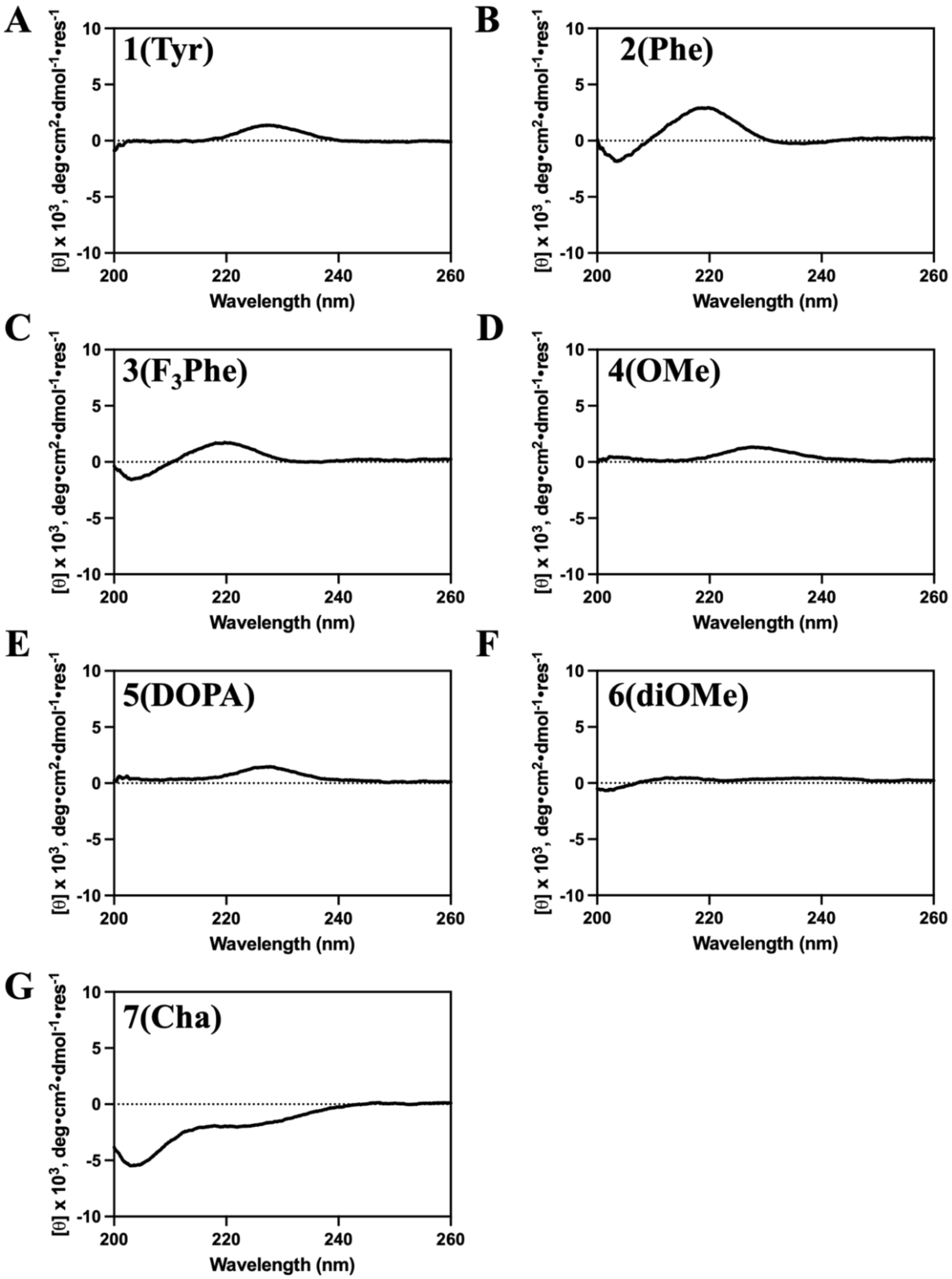

Figure S6. Far-UV CD for the seven peptides discussed in the main text, each at 50 μM in PBS, pH 7.4 at 25°C. The Tris buffer used for other experiments is not compatible with CD (very poor signal-to-noise ratio below 210 nm). Data points with high tension (HT) over 700 V have been excluded from the analysis. We conclude that each of the peptides in this study remains largely unfolded in a neutral pH buffer, as expected in light of the high glycine content (15 of 28 residues).

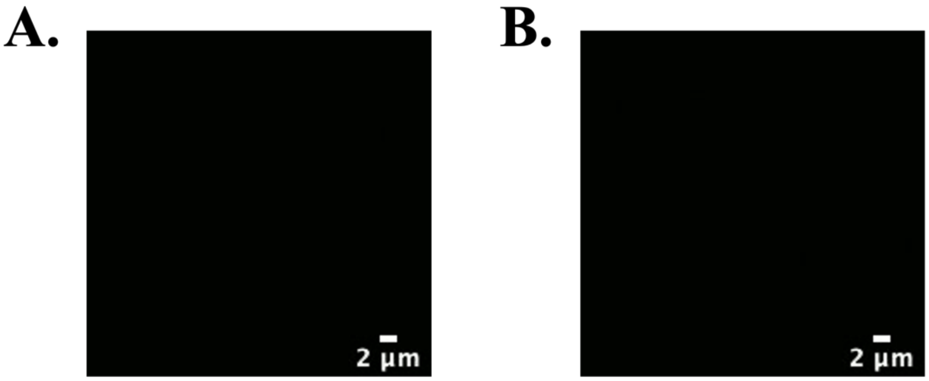

Figure S7. Representative confocal images of the mixture between 5 μM FUS-C and 50 μM **8(Ser)** (1 mol% fluorescein-**8(Ser)**, **A**), 50 μM **9(Ala)** (1 mol% fluorescein-**9(Ala)**, **B**). Scale bar = 2 μm. No droplets were observed 30 min after the mixture. Buffer condition: 50 mM Tris, pH 7.5, 150 mM NaCl and 0.5 mM TCEP.

Protein sequence: MBP-TEV-FUS(372-526)-H_6

With MBP Tag:
ATGAAAATCGAAGAAGGTAAACTGGTAATCTGGATTAACGGCGATAAAGGCTATAA
CGGTCTCGCTGAAGTCGGTAAGAAATTCGAGAAAGATACCGGAATTAAAGTCACCG
TTGAGCATCCGGATAAACTGGAAGAGAAATTCCCACAGGTTGCGGCAACTGGCGAT
GGCCCTGACATTATCTTCTGGGCACACGACCGCTTTGGTGGCTACGCTCAATCTGGC
CTGTTGGCTGAAATCACCCCGGACAAAGCGTTCCAGGACAAGCTGTATCCGTTTACC
TGGGATGCCGTACGTTACAACGGCAAGCTGATTGCTTACCCGATCGCTGTTGAAGCG
TTATCGCTGATTTATAACAAAGATCTGCTGCCGAACCCGCCAAAAACCTGGGAAGA
GATCCCGGCGCTGGATAAAGAACTGAAAGCGAAAGGTAAGAGCGCGCTGATGTTCA
ACCTGCAAGAACCGTACTTCACCTGGCCGCTGATTGCTGCTGACGGGGGTTATGCGT
TCAAGTATGAAAACGGCAAGTACGACATTAAAGACGTGGGCGTGGATAACGCTGGC
GCGAAAGCGGGTCTGACCTTCCTGGTTGACCTGATTAAAAACAAACACATGAATGC
AGACACCGATTACTCCATCGCAGAAGCTGCCTTTAATAAAGGCGAAACAGCGATGA
CCATCAACGGCCCGTGGGCATGGTCCAACATCGACACCAGCAAAGTGAATTATGGT
GTAACGGTACTGCCGACCTTCAAGGGTCAACCATCCAAACCGTTCGTTGGCGTGCTG
AGCGCAGGTATTAACGCCGCCAGTCCGAACAAAGAGCTGGCAAAAGAGTTCCTCGA
AAACTATCTGCTGACTGATGAAGGTCTGGAAGCGGTTAATAAAGACAAACCGCTGG
GTGCCGTAGCGCTGAAGTCTTACGAGGAAGAGTTGGTGAAAGATCCGCGTATTGCC
GCCACTATGGAAAACGCCCAGAAAGGTGAAATCATGCCGAACATCCCGCAGATGTC
CGCTTTCTGGTATGCCGTGCGTACTGCGGTGATCAACGCCGCCAGCGGTCGTCAGAC
TGTCGATGAAGCCCTGAAAGACGCGCAGACTAATTCGAGCTCGAACAACAACAACA
ATAACAATAACAACAACCTCGGGGAAAACTTATACTTTCAGGGGCGCGCGGACTTT
AACCGCGGGGGCGGAAATGGCCGTGGGGGTCGTGGCCGTGGGGGTCCTATGGGCCG
CGGAGGCTATGGCGGAGGTGGTTCGGGTGGAGGCGGTCGCGGGGGCTTTCCTTCGG
GAGGGGGCGGCGGTGGGGGACAGCAGCGCGCAGGCGATTGGAAATGTCCGAATCC
GACCTGCGAGAATATGAATTTTAGTTGGCGCAATGAATGCAATCAGTGTAAAGCCCC
GAAACCGGATGGTCCAGGCGGGGGTCCAGGTGGGAGCCACATGGGGGGAAATTATG
GAGATGATCGCCGCGGCGGTCGTGGGGGTTATGATCGTGGTGGTTATCGTGGGCGTG
GGGGAGATCGTGGGGGATTTCGTGGCGGGCGCGGTGGGGGTGATCGTGGCGGTTTT
GGGCCTGGGAAAATGGATTCGCGTGGCGAGCACCGCCAAGATCGCCGCGAGCGCCC
GTATCATCATCACCATCATCATTAAGAATTC

Translation:
MKIEEGKLVIWINGDKGYNGLAEVGKKFEKDTGIKVTVEHPDKLEEKFPQVAATGDGP
DIIFWAHDRFGGYAQSGLLAEITPDKAFQDKLYPFTWDAVRYNGKLIAYPIAVEALSLIY
NKDLLPNPPKTWEEIPALDKELKAKGKSALMFNLQEPYFTWPLIAADGGYAFKYENGK
YDIKDVGVDNAGAKAGLTFLVDLIKNKHMNADTDYSIAEAAFNKGETAMTINGPWAW
SNIDTSKVNYGVTVLPTFKGQPSKPFVGVLSAGINAASPNKELAKEFLENYLLTDEGLEA
VNKDKPLGAVALKSYEEELVKDPRIAATMENAQKGEIMPNIPQMSAFWYAVRTAVINA
ASGRQTVDEALKDAQTNSSSNNNNNNNNNNLGENLYFQGRADFNRGGGNGRGGRGR
GGPMGRGGYGGGGSGGGGRGGFPSGGGGGGGQQRAGDWKCPNPTCENMNFSWRNE
CNQCKAPKPDGPGGGPGGSHMGGNYGDDRRGGRGGYDRGGYRGRGGDRGGFRGGR
GGGDRGGFGPGKMDSRGEHRQDRRERPYHHHHHH

Protein sequence: FUS

MASNDYTQQATQSYGAYPTQPGQGYSQQSSQPYGQQSYSGYSQSTDTSGYGQSSYSSY
GQSQNTGYGTQSTPQGYGSTGGYGSSQSSQSSYGQQSSYPGYGQQPAPSSTSGSYGSSS
QSSSYGQPQSGSYSQQPSYGGQQQSYGQQQSYNPPQGYGQQNQYNSSSGGGGGGGG
GNYGQDQSSMSSGGGSGGGYGNQDQSGGGGSGGYGQQDRGGRGRGGSGGGGGGGG
GGYNRSSGGYEPRGRGGGRGGRGGMGGSDRGGFNKFGGPRDQGSRHDSEQDNSDNNT
IFVQGLGENVTIESVADYFKQIGIIKTNKKTGQPMINLYTDRETGKLKGEATVSFDDPPSA
KAAIDWFDGKEFSGNPIKVSFATRRADFNRGGGNGRGGRGRGGPMGRGGYGGGGSGG
GGRGGFPSGGGGGGGQQRAGDWKCPNPTCENMNFSWRNECNQCKAPKPDGPGGGPG
GSHMGGNYGDDRRGGRGGYDRGGYRGRGGDRGGFRGGRGGGDRGGFGPGKMDSRG
EHRQDRRERPY

Energies

Structures	Electronic Energy (hartrees)	Gibbs Free Energy (hartrees)
A	-314.3136844	-314.240635
A'	-306.8830147	-306.807934
B	-390.6595304	-390.567786
B'	-383.2287375	-383.135332
C	-467.0026154	-466.893015
C'	-459.5717225	-459.460183
D	-353.5198931	-353.419939
D'	-346.0892458	-345.986943
E	-429.8623223	-429.745274
E'	-422.4318672	-422.312918
F	-467.8585959	-467.728085
F'	-460.4278138	-460.295145
G	-544.2027785	-544.055115
G'	-536.7720475	-536.622511
Li	-7.430170106	-7.442918

Table S1. Calculated energies for all structures, with (X) and without Li cation (X').

Dissociation energies (ΔG)

Structures	Electronic Energy (kcal/mol)	Gibbs Free Energy (kcal/mol)
A	0.31	-6.41
B	0.39	-6.57
C	0.45	-6.33
D	0.30	-6.23
E	0.18	-6.63
F	0.38	-6.26
G	0.35	-6.47

Table S2. Dissociation energies of all structures.

IV. MALDI-TOF-MS, ESI-MS and UPLC Characterization of Peptides

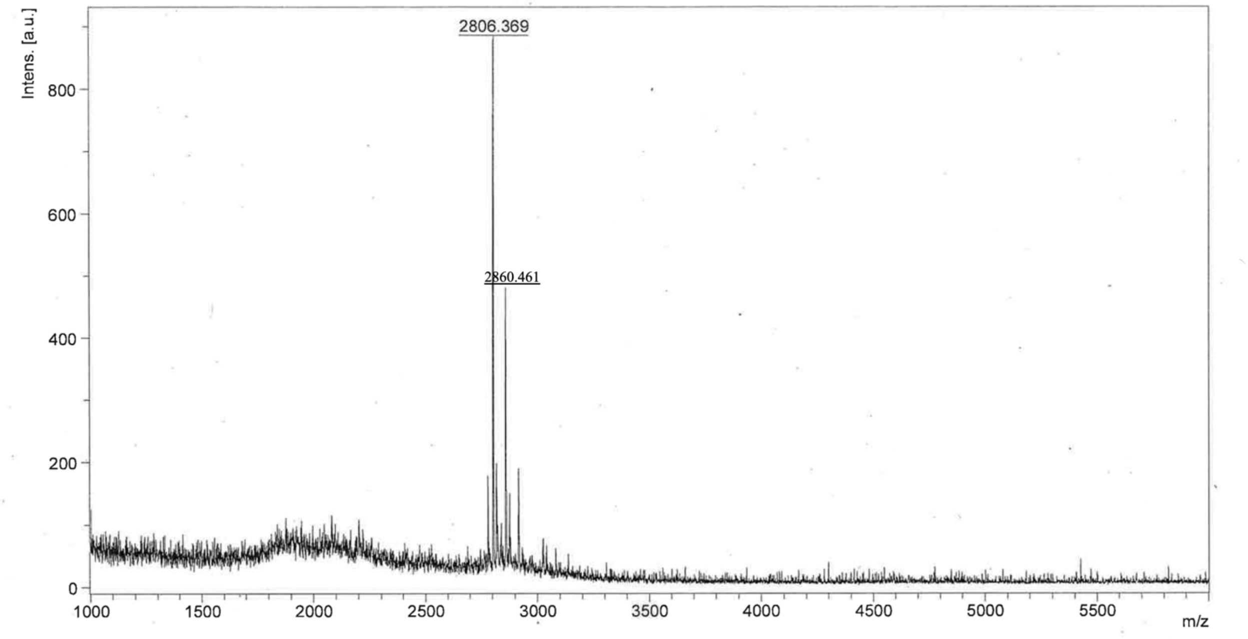

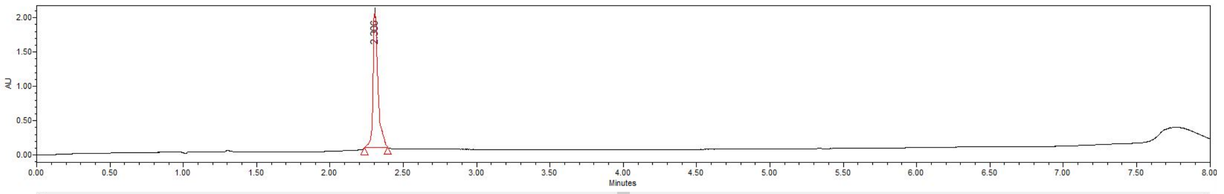

1 (Tyr): H_2N-GGGYEEEYGGGYGGGYEEEYGGGYGGGY-NH_2

MALDI-TOF-MS: calculated monoisotopic $[M+NH_4]^+$ = 2806.137 $[M+4NH_4]^+$ = 2860.137
observed monoisotopic $[M+NH_4]^+$ = 2806.369 $[M+4NH_4]^+$ = 2860.461

UPLC: MPA: H_2O + 0.1% TFA, MPB: MeCN + 0.1% TFA, 10-95% MeCN, 5 min, 0.3 mL/min on an ACQUITY Premier CSH C18 (130 Å 1.7 µm, 2.1 x 150 mm) column.

Purity > 99.0 %

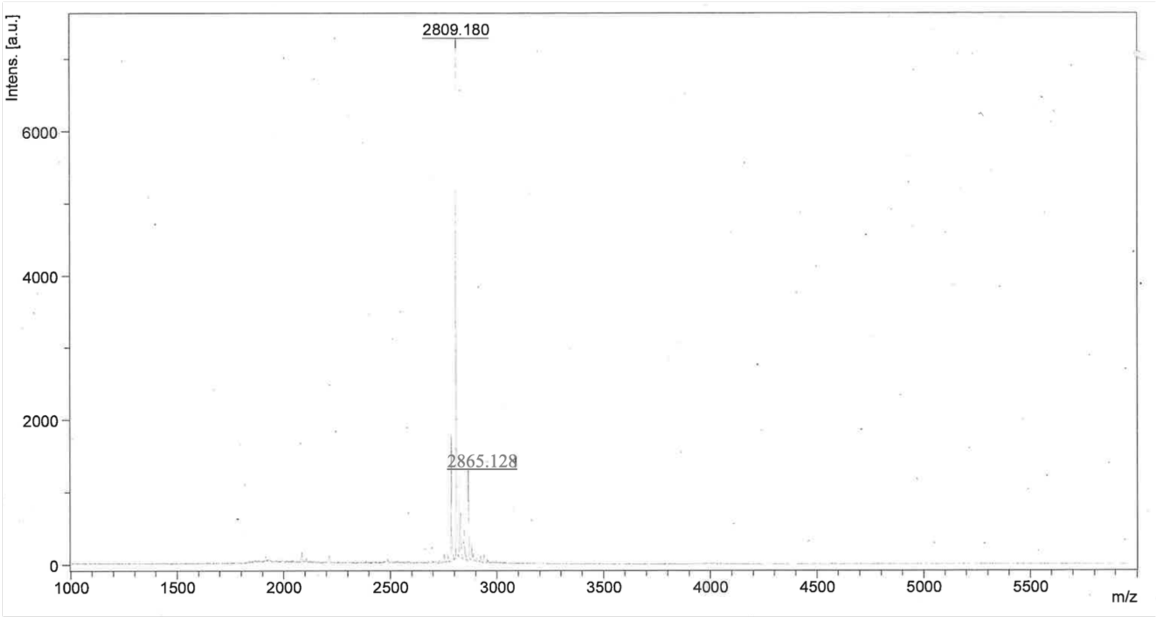

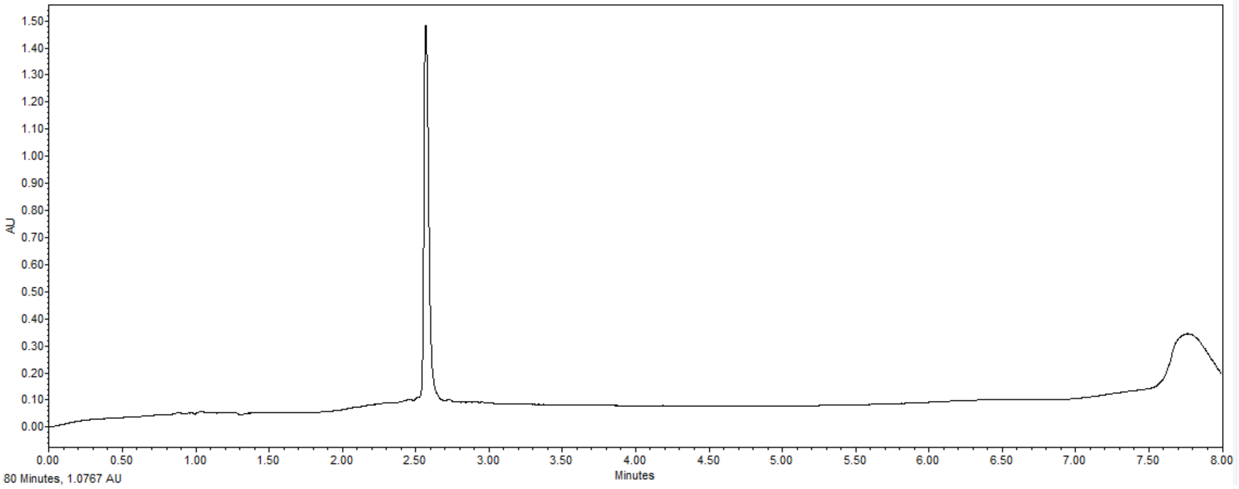

1a: **H_2N-GGGYKKKYGGGYGGGYEEEYGGGYGGGY-NH_2**

MALDI-TOF-MS: calculated monoisotopic $[M+Na]^+$ = 2809.886 $[M+DMSO+H]^+$ = 2865.886
observed monoisotopic $[M+Na]^+$ = 2809.180 $[M+DMSO+H]^+$ = 2865.128

UPLC: MPA: H_2O + 0.1% TFA, MPB: MeCN + 0.1% TFA, 10-95% MeCN, 5 min, 0.3 mL/min on an ACQUITY Premier CSH C18 (130 Å 1.7 µm, 2.1 x 150 mm) column.

Purity > 99.0 %

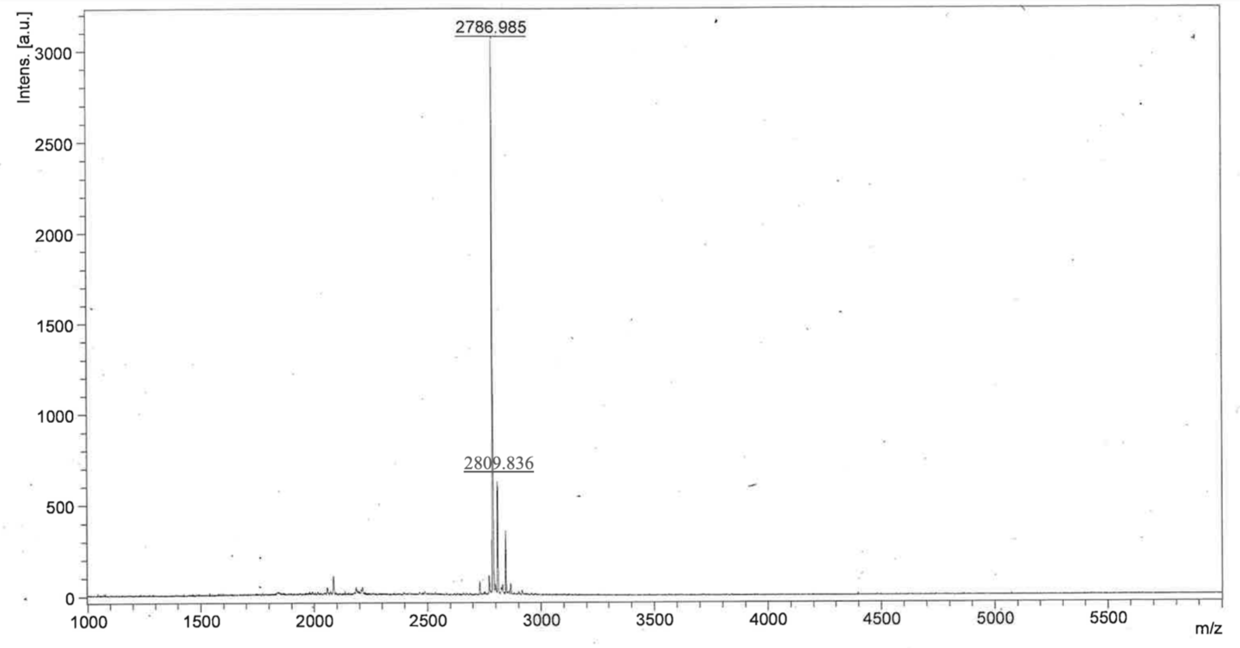

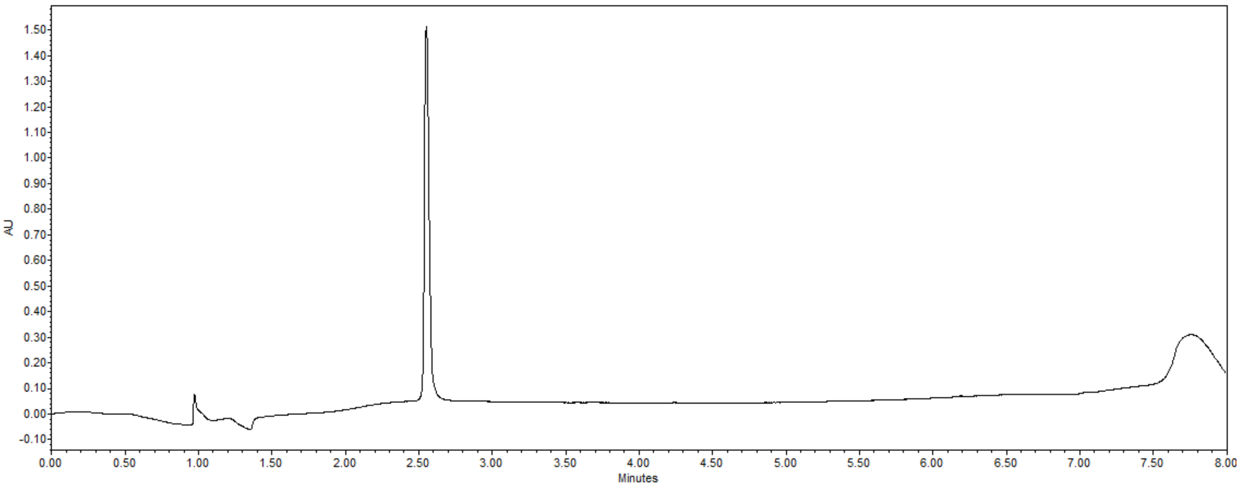

1b: **H_2N-GGGYEEEYGGGYGGGYKKKYGGGYGGGY-NH_2**

MALDI-TOF-MS: calculated monoisotopic $[M+H]^+ = 2786.886$ $[M+Na]^+ = 2809.886$
observed monoisotopic $[M+H]^+ = 2786.985$ $[M+Na]^+ = 2809.836$

UPLC: MPA: H_2O + 0.1% TFA, MPB: MeCN + 0.1% TFA, 10-95% MeCN, 5 min, 0.3 mL/min on an ACQUITY Premier CSH C18 (130 Å 1.7 µm, 2.1 x 150 mm) column.

Purity > 99.0 %

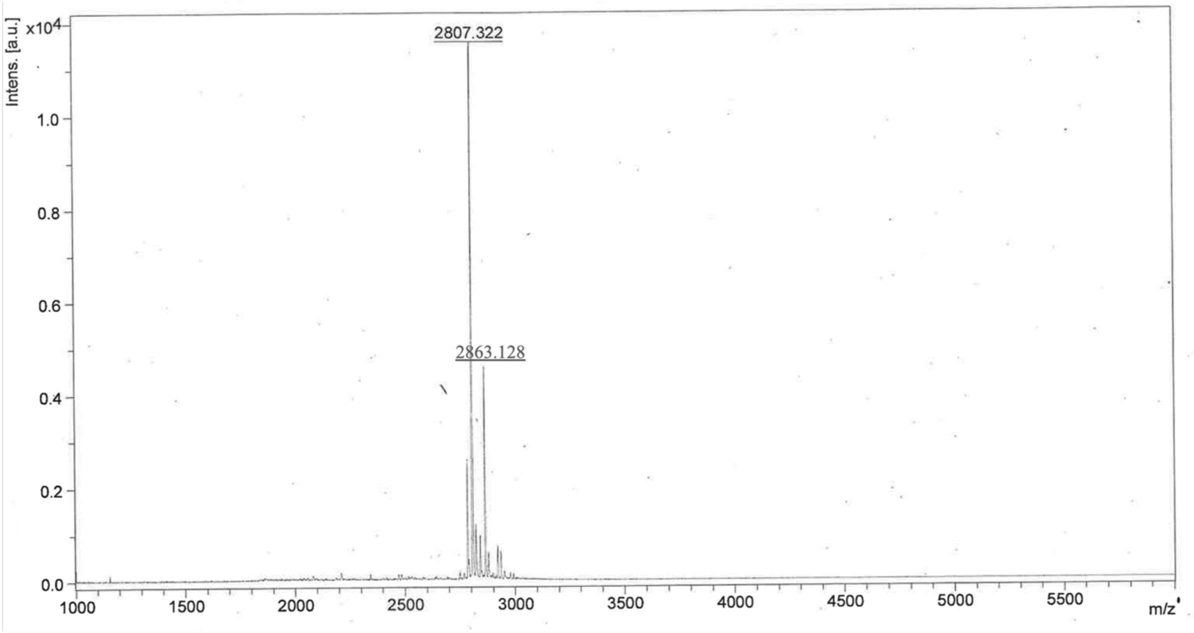

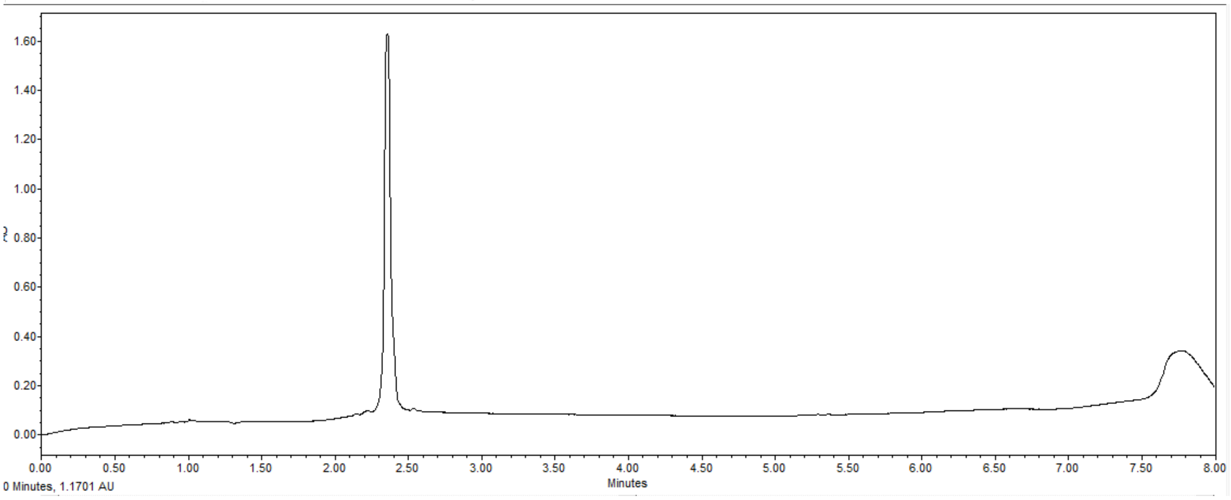

1c: **H_2N-GGGYKKKYGGGYGGGYKKKYGGGYGGGY-NH_2**

MALDI-TOF-MS: calculated monoisotopic $[M+Na]^+$ = 2807.062 $[M+DMSO+H]^+$ = 2863.062
observed monoisotopic $[M+Na]^+$ = 2807.322 $[M+DMSO+H]^+$ = 2863.128

UPLC: MPA: H_2O + 0.1% TFA, MPB: MeCN + 0.1% TFA, 10-95% MeCN, 5 min, 0.3 mL/min on an ACQUITY Premier CSH C18 (130 Å 1.7 µm, 2.1 x 150 mm) column.

Purity > 99.0 %

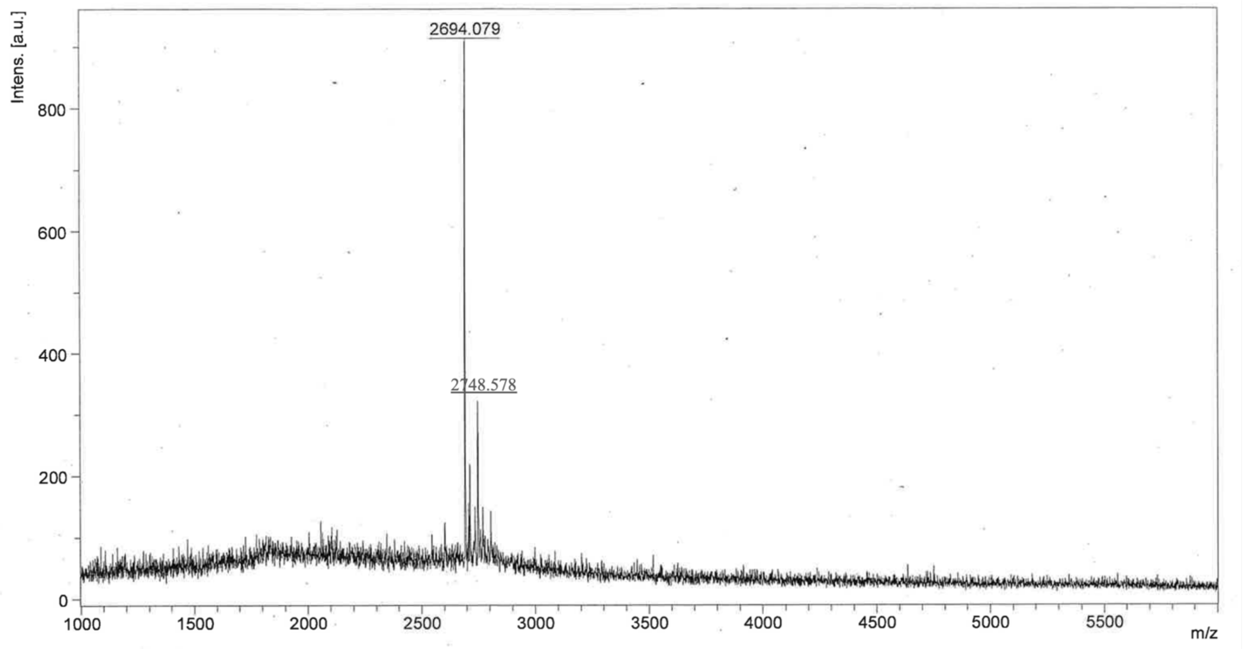

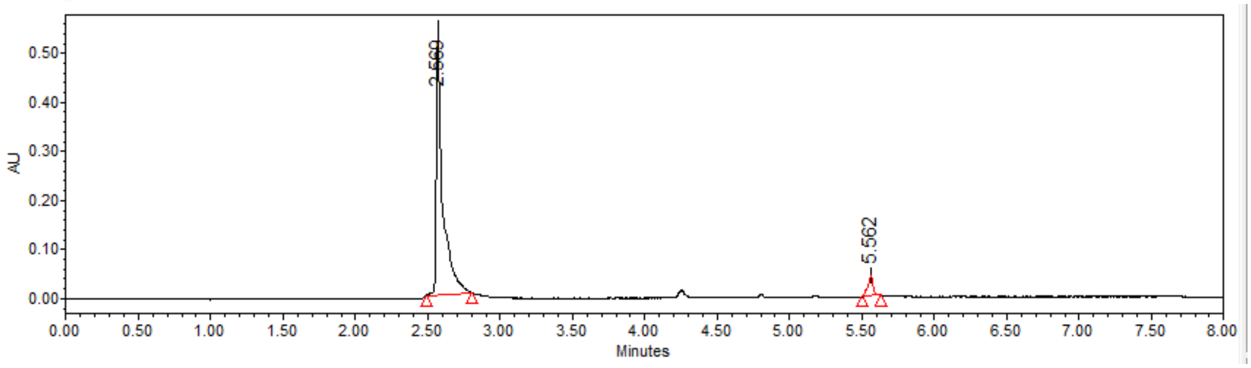

2(Phe): **H_2N-GGGFEEEFGGGFGGGFEEEFGGGFGGGF-NH_2**

MALDI-TOF-MS: calculated monoisotopic $[M+NH_4]^+$ = 2694.072 $[M+4NH_4]^+$ = 2748.072
observed monoisotopic $[M+NH_4]^+$ = 2694.079 $[M+4NH_4]^+$ = 2748.578

UPLC: MPA: H_2O + 0.1% TFA, MPB: MeCN + 0.1% TFA, 10-95% MeCN, 5 min, 0.3 mL/min on an ACQUITY Premier CSH C18 (130 Å 1.7 µm, 2.1 x 150 mm) column.

Purity = 94.1 %

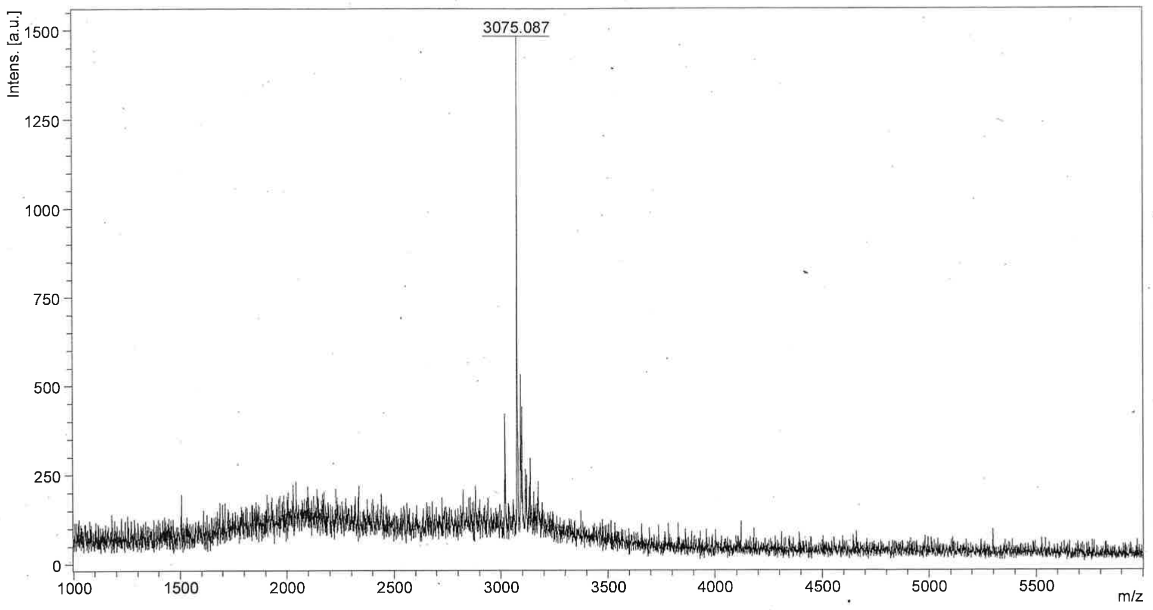

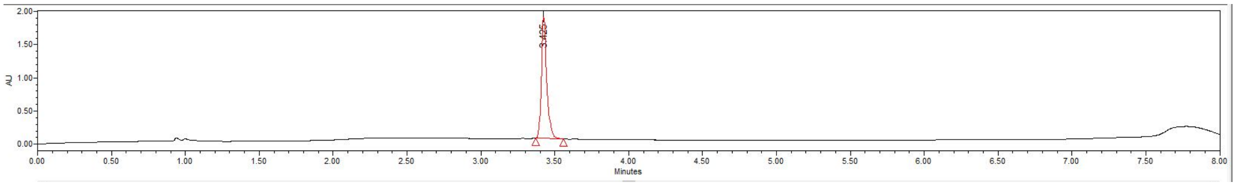

3(F_3Phe): H_2N-GGGXEEEXGGGXGGGXEEEXGGGXGGGX-NH_2 (X = F_3Phe)
<u>MALDI-TOF-MS</u>: calculated monoisotopic $[M+NH_4]^+$ = 3073.640
observed monoisotopic $[M+NH_4]^+$ = 3075.087

<u>UPLC</u>: MPA: H_2O + 0.1% TFA, MPB: MeCN + 0.1% TFA, 10-95% MeCN, 5 min, 0.3 mL/min on an ACQUITY Premier CSH C18 (130 Å 1.7 µm, 2.1 x 150 mm) column.
Purity = 95.09 %

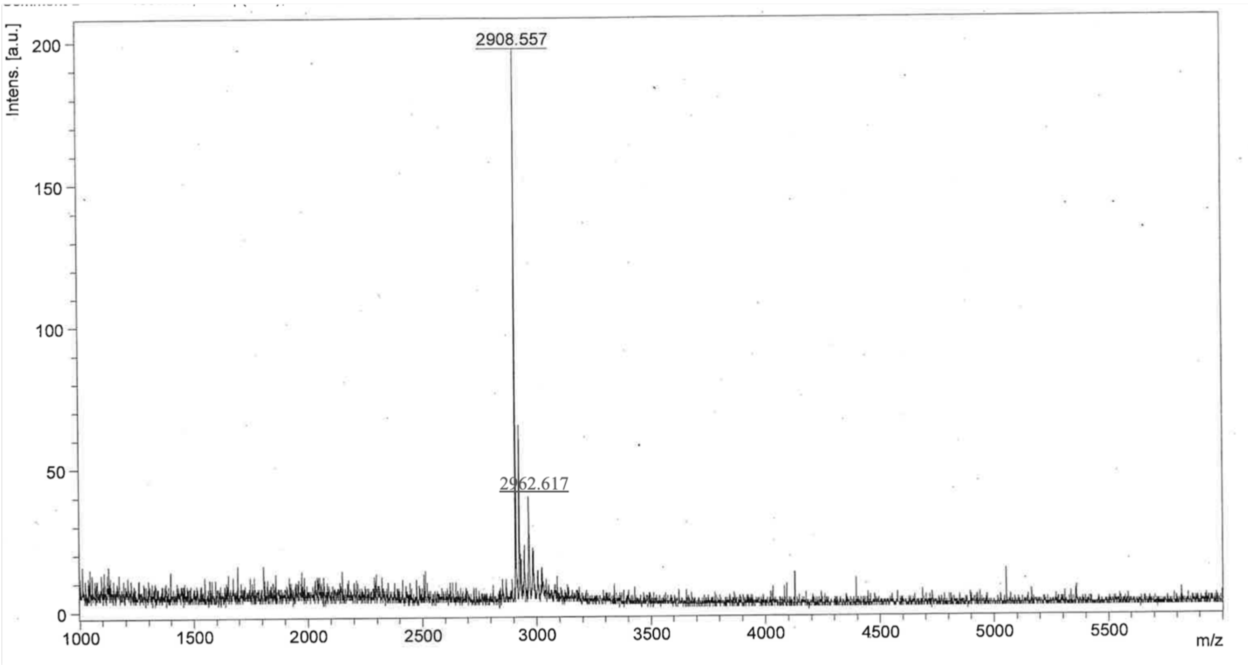

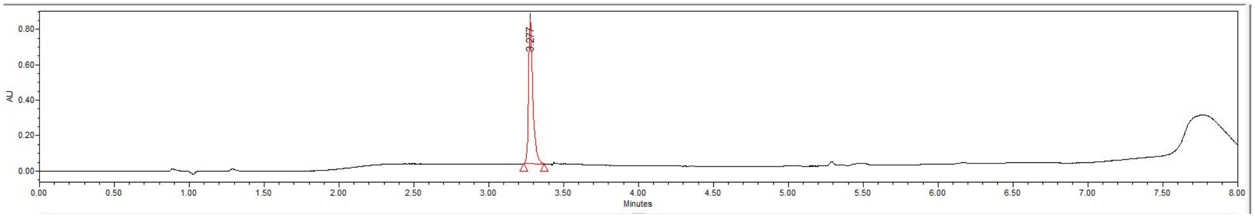

4(TyrOMe): **`H₂N-GGGXEEEXGGGXGGGXEEEXGGGXGGGX-NH₂   (X = TyrOMe)`**

<u>MALDI-TOF-MS</u>: calculated monoisotopic $[M+NH_4]^+$ = 2906.020 $[M+4NH_4]^+$ = 2962.020
observed monoisotopic $[M+NH_4]^+$ = 2908.557 $[M+4NH_4]^+$ = 2962.617

<u>UPLC</u>: MPA: H_2O + 0.1% TFA, MPB: MeCN + 0.1% TFA, 10-95% MeCN, 5 min, 0.3 mL/min on an ACQUITY Premier CSH C18 (130 Å 1.7 µm, 2.1 x 150 mm) column.
Purity > 99.0 %

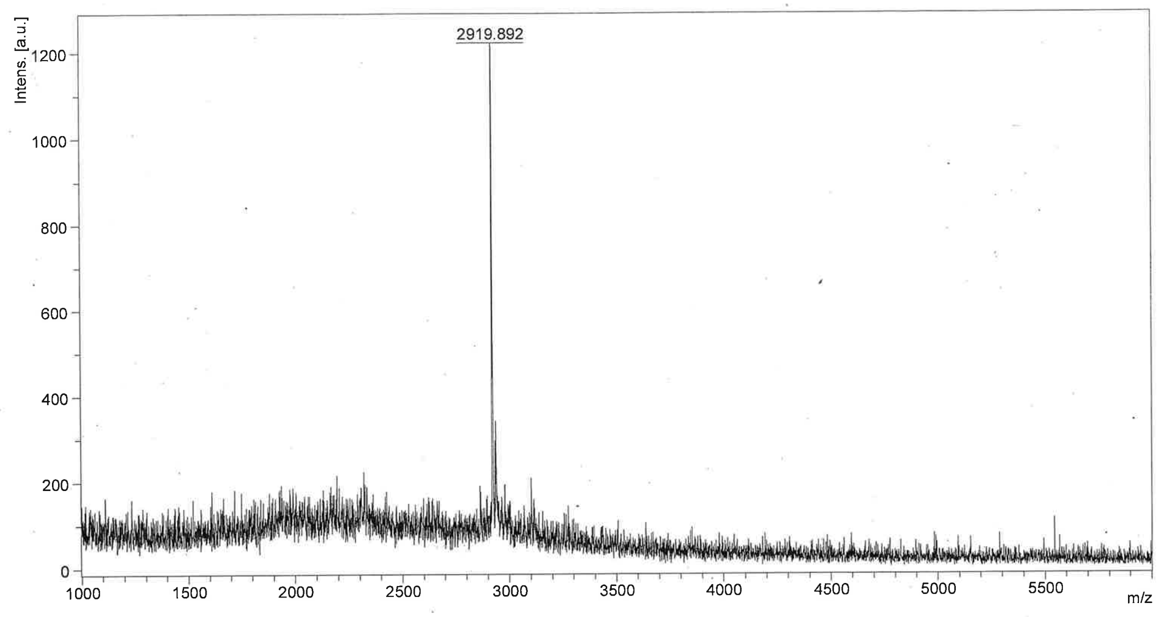

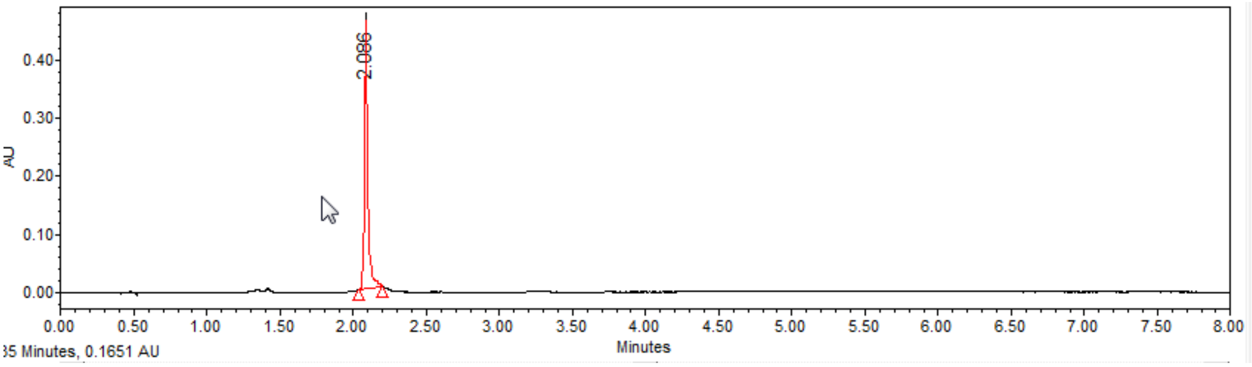

5(DOPA): H_2N-GGGXEEEXGGGXGGGXEEEXGGGXGGGX-NH_2 (X = DOPA)
MALDI-TOF-MS: calculated monoisotopic $[M+NH_4]^+$ = 2919.83
observed monoisotopic $[M+NH_4]^+$ = 2919.892

UPLC: MPA: H_2O + 0.1% TFA, MPB: MeCN + 0.1% TFA, 10-95% MeCN, 5 min, 0.3 mL/min on an ACQUITY Premier CSH C18 (130 Å 1.7 µm, 2.1 x 150 mm) column.
Purity > 99.0 %

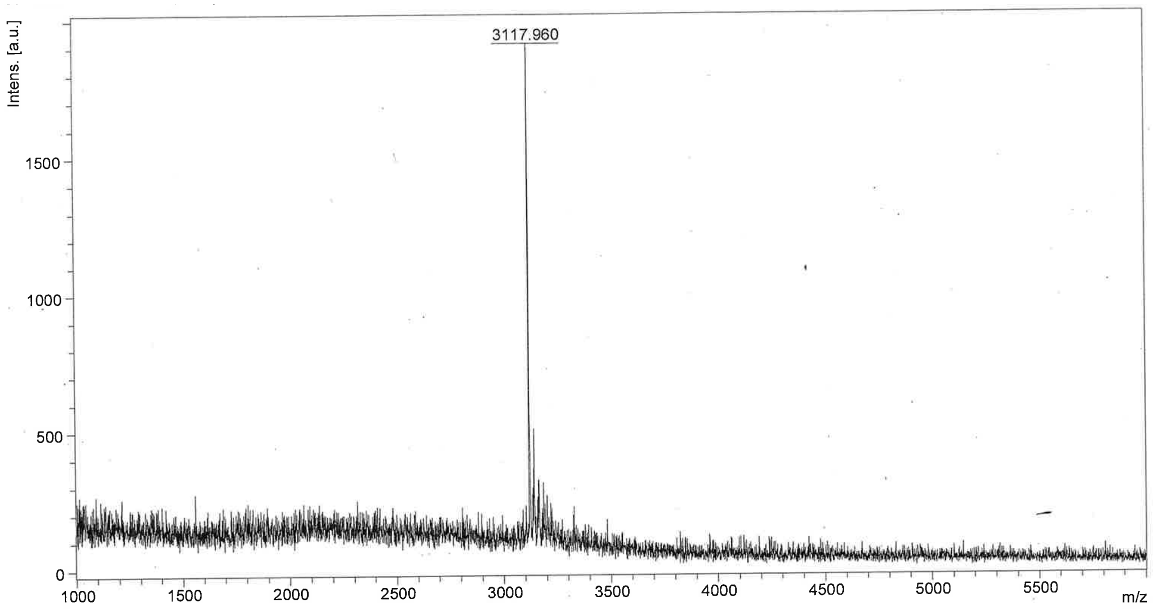

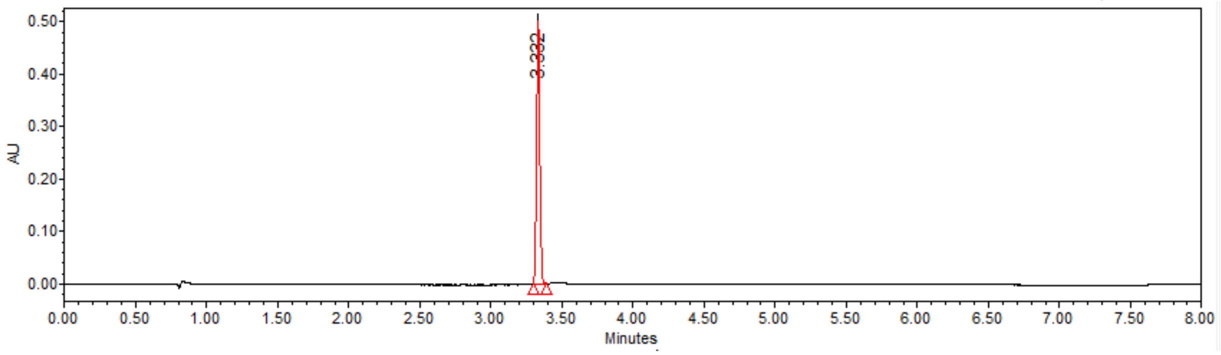

6(diOMe): H_2N-GGGXEEEXGGGXGGGXEEEXGGGXGGGX-NH_2 (X = diOMe)

MALDI-TOF-MS: calculated monoisotopic $[M+NH_4]^+$ = 3117.96
observed monoisotopic $[M+NH_4]^+$ = 3116.20

UPLC: MPA: H_2O + 0.1% TFA, MPB: MeCN + 0.1% TFA, 10-95% MeCN, 5 min, 0.3 mL/min on an ACQUITY Premier CSH C18 (130 Å 1.7 μm, 2.1 x 150 mm) column.

Purity > 99.0 %

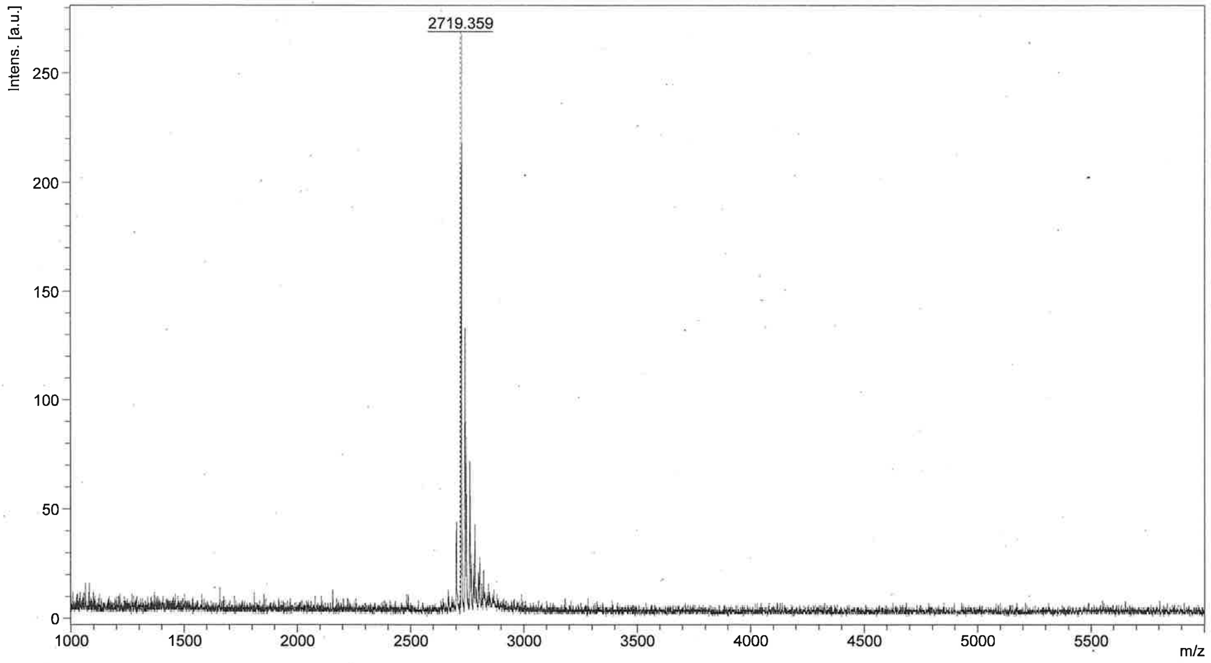

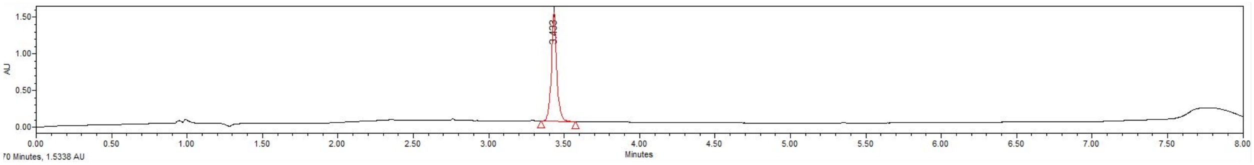

7(Cha): H_2N-GGGXEEEXGGGXGGGXEEEXGGGXGGGX-NH_2 (X = Cha)

MALDI-TOF-MS: calculated monoisotopic $[M+H]^+ = 2719.41$
observed monoisotopic $[M+H]^+ = 2719.359$

UPLC: MPA: H_2O + 0.1% TFA, MPB: MeCN + 0.1% TFA, 10-95% MeCN, 5 min, 0.3 mL/min on an ACQUITY Premier CSH C18 (130 Å 1.7 µm, 2.1 x 150 mm) column.
Purity > 99.0 %

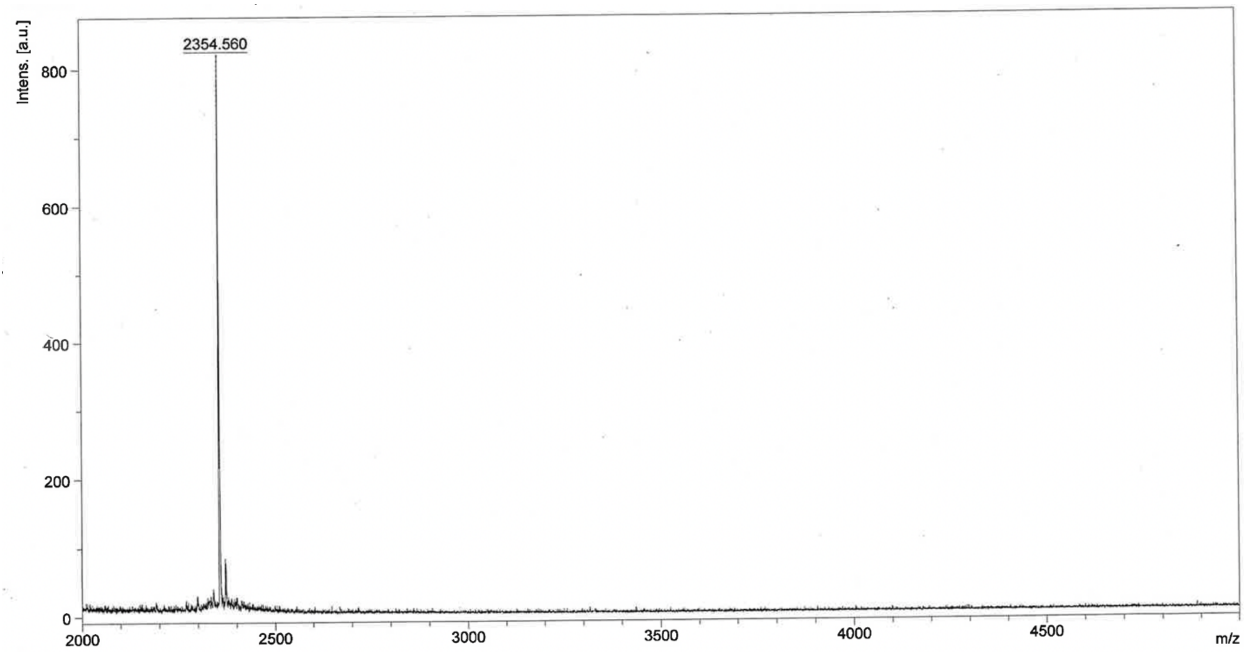

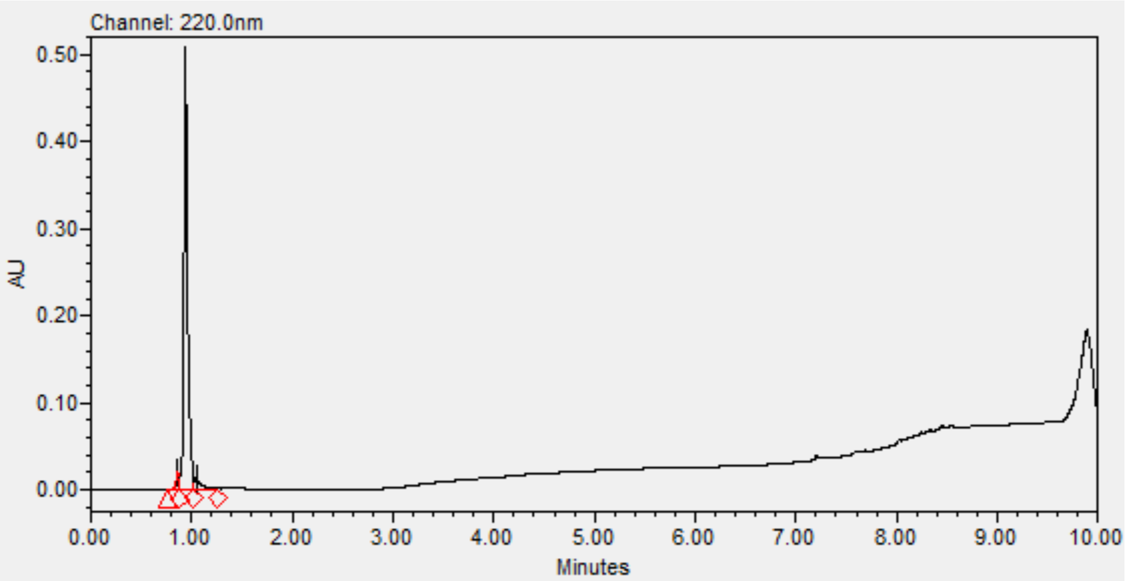

8(Ser): H_2N-GGGSEEESGGGSGGGSEEESGGGSGGGY-NH_2
MALDI-TOF-MS: calculated monoisotopic $[M+Na]^+$ = 2354.849
observed monoisotopic $[M+Na]^+$ = 2354.560

UPLC: MPA: H_2O + 10 mM ammonium formate, MPB: 9:1 (v/v %) MeCN:H_2O + 10 mM ammonium formate, 10-95% MPB, 5 min, 0.3 mL/min on an ACQUITY Premier BEH C18 (130 Å 1.7 μm, 2.1 x 150 mm) column.

Purity = 92.0 %

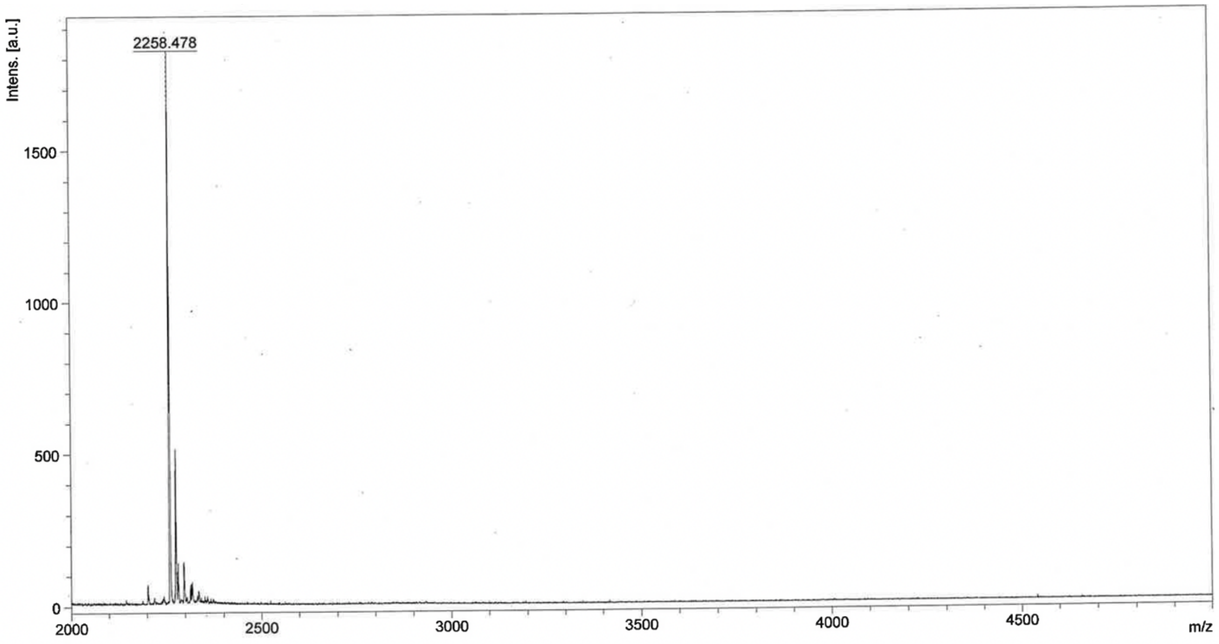

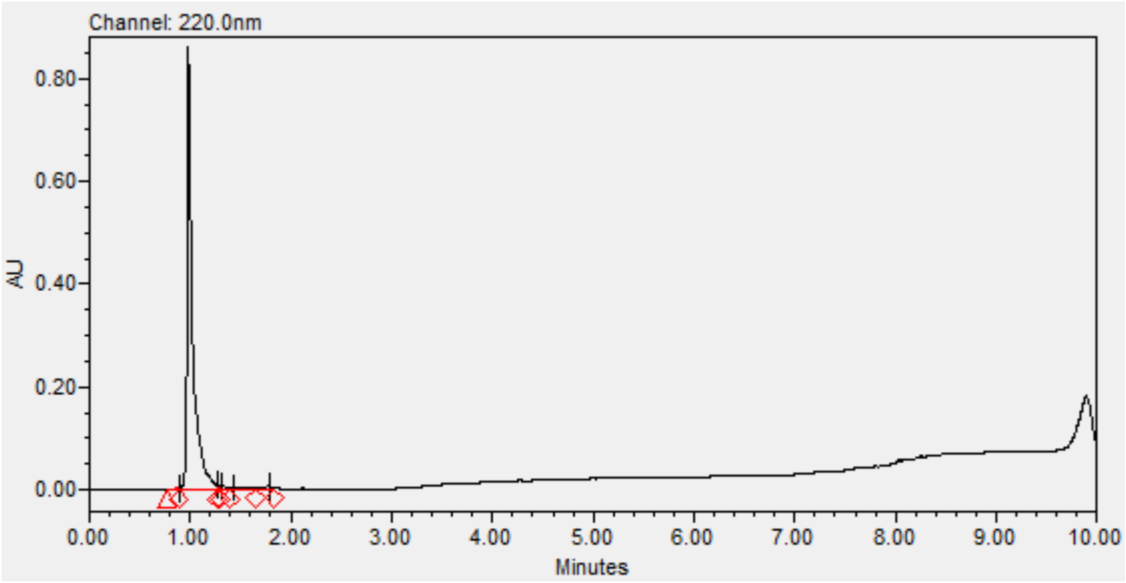

9(Ala): H_2N-GGGAEEEAGGGAGGGAEEEAGGGAGGGY-NH_2

MALDI-TOF-MS: calculated monoisotopic $[M+Na]^+ = 2358.879$
observed monoisotopic $[M+Na]^+ = 2358.478$

UPLC: MPA: H_2O + 10 mM ammonium formate, MPB: 9:1 (v/v %) MeCN:H_2O + 10 mM ammonium formate, 10-95% MPB, 5 min, 0.3 mL/min on an ACQUITY Premier BEH C18 (130 Å 1.7 µm, 2.1 x 150 mm) column.

Purity = 95.2 %

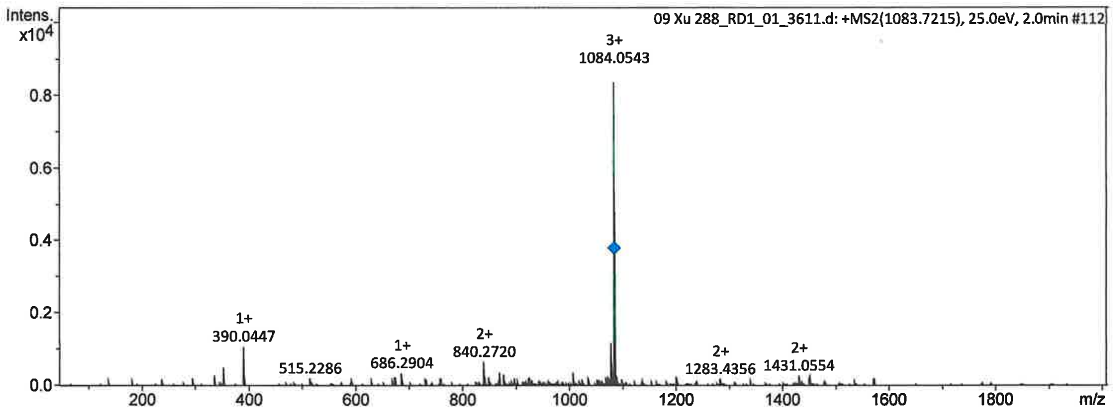

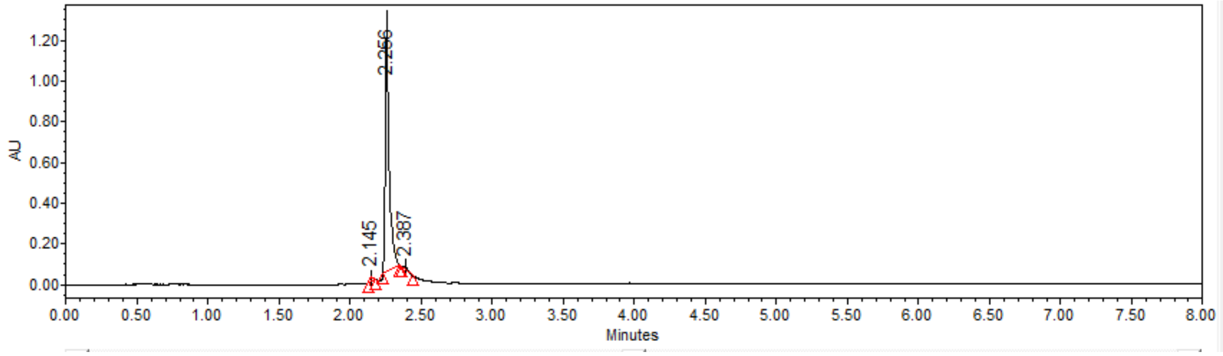

FITC-1(Tyr): FITC-(βAla)-GGGYEEEYGGGYGGGYEEEYGGGYGGGY-NH_2

ESI-MS: calculated monoisotopic $[M+3H]^{3+}$ = 1084.063
observed monoisotopic $[M+3H]^{3+}$ = 1084.054
(*ESI-MS was used for this peptide due to high laser fragmentation of the fluorescein ground under MALDI-TOF-MS condition)

UPLC: H_2O/MeCN + 0.1% TFA, 10-95% MeCN, 5 min, 0.3 mL/min on an ACQUITY Premier CSH C18 (130 Å 1.7 µm, 2.1 x 150 mm) column.
Purity = 97.38 %

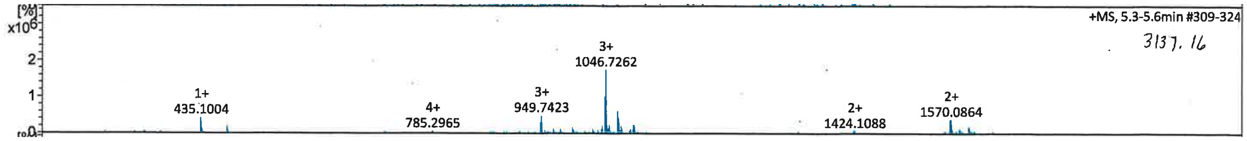

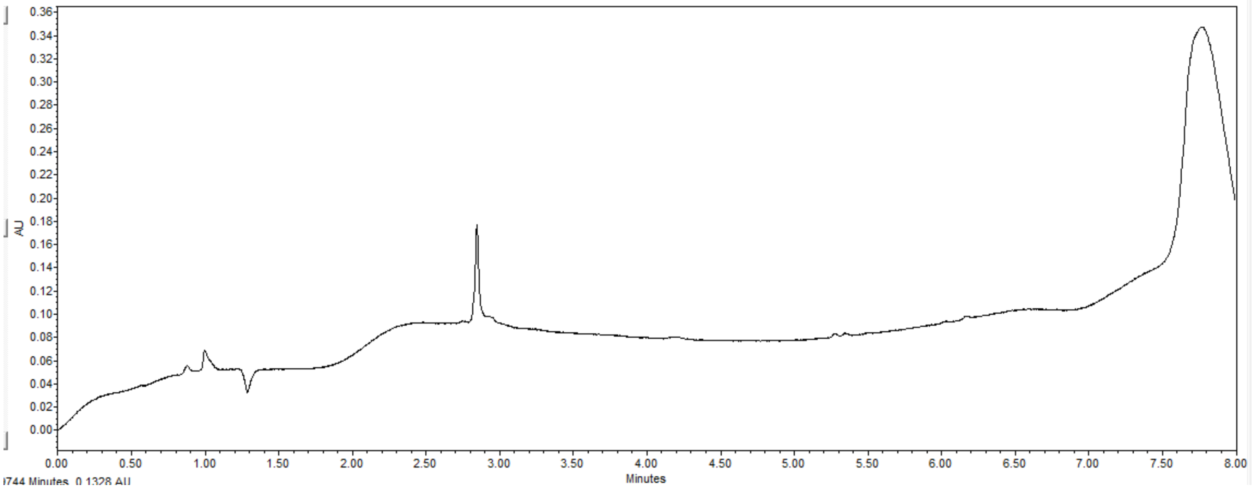

FITC-2(Phe): **`FITC-(βAla)-GGGFEEEFGGGFGGGFEEEFGGGFGGGF-NH₂`**

ESI-MS: calculated monoisotopic $[M+3H]^{3+}$ = 1046.0533
observed monoisotopic $[M+3H]^{3+}$ = 1046.7262
(*ESI-MS was used for this peptide due to high laser fragmentation of the fluorescein ground under MALDI-TOF-MS condition)

UPLC: H_2O/MeCN + 0.1% TFA, 10-95% MeCN, 5 min, 0.3 mL/min on an ACQUITY Premier CSH C18 (130 Å 1.7 µm, 2.1 x 150 mm) column.

Purity = 95.24 %

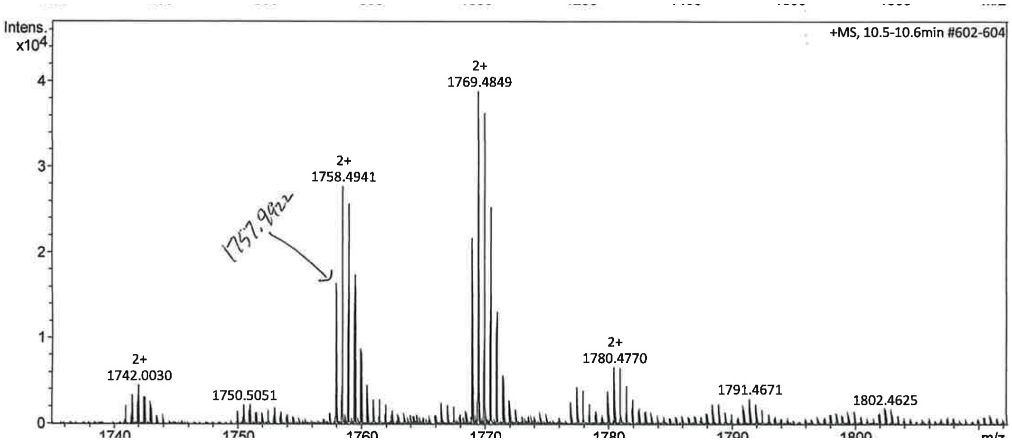

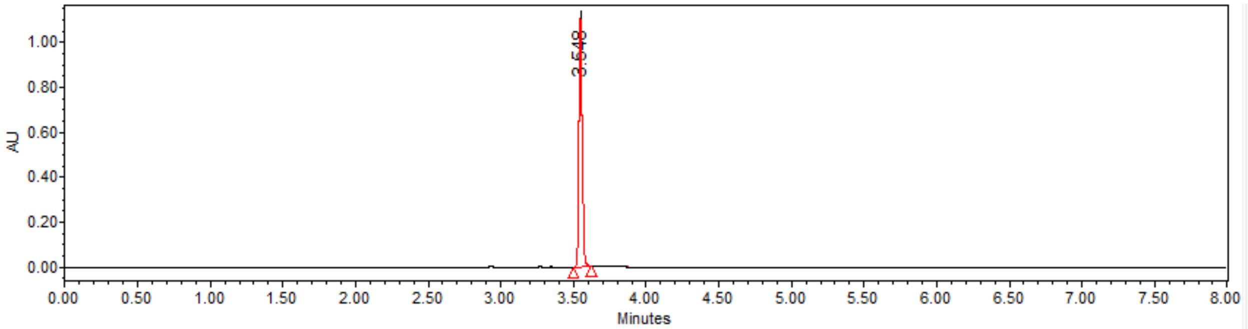

FITC-3(F_3Phe): `FITC-(βAla)-GGGXEEEXGGGXGGGXEEEXGGGXGGGX-NH₂` (X = F_3Phe)

ESI-MS: calculated monoisotopic $[M+2H]^{2+}$ = 1757.983 $[M+Na+2H]^{2+}$ = 1769.483
observed monoisotopic $[M+2H]^{2+}$ = 1757.992 $[M+Na+2H]^{2+}$ = 1769.485
(*ESI-MS was used for this peptide due to high laser fragmentation of the fluorescein ground under MALDI-TOF-MS condition)

UPLC: H_2O/MeCN + 0.1% TFA, 10-95% MeCN, 5 min, 0.3 mL/min on an ACQUITY Premier CSH C18 (130 Å, 1.7 µm, 2.1 x 150 mm) column.
Purity > 99.0 %

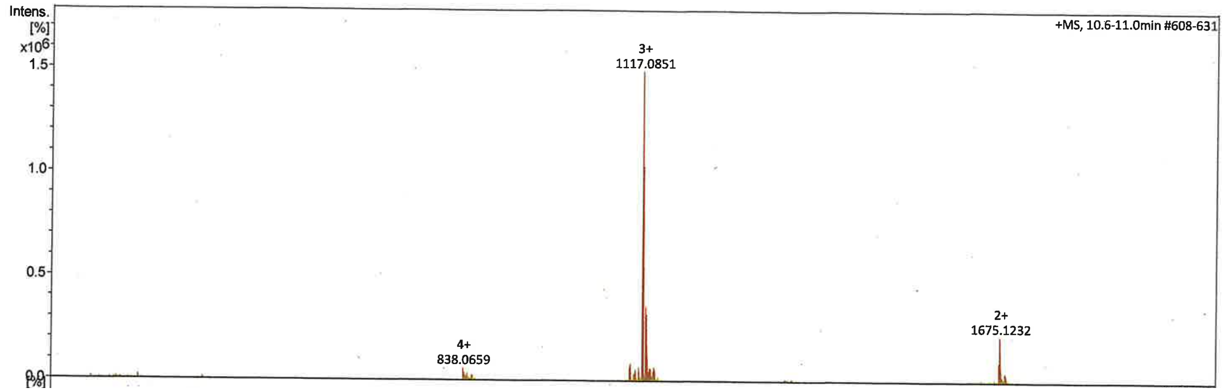

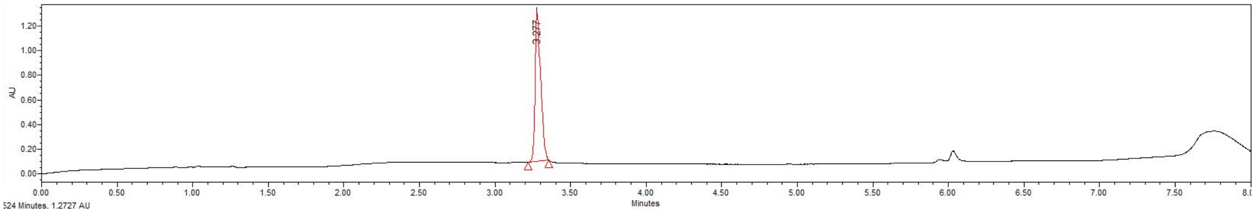

FITC-4(TyrOMe): `FITC-(βAla)-GGGXEEEXGGGXGGGXEEEXGGGXGGGX-NH₂ (X = TyrOMe)`

ESI-MS: calculated monoisotopic $[M+3H]^{3+}$ = 1116.412 $[M+2H]^{2+}$ = 1674.118
observed monoisotopic $[M+3H]^{3+}$ = 1117.085 $[M+2H]^{2+}$ = 1675.123
(*ESI-MS was used for this peptide due to high laser fragmentation of the fluorescein ground under MALDI-TOF-MS condition)

UPLC: H_2O/MeCN + 0.1% TFA, 10-95% MeCN, 5 min, 0.3 mL/min on an ACQUITY Premier CSH C18 (130 Å, 1.7 µm, 2.1 x 150,mm) column.
Purity > 99.0 %

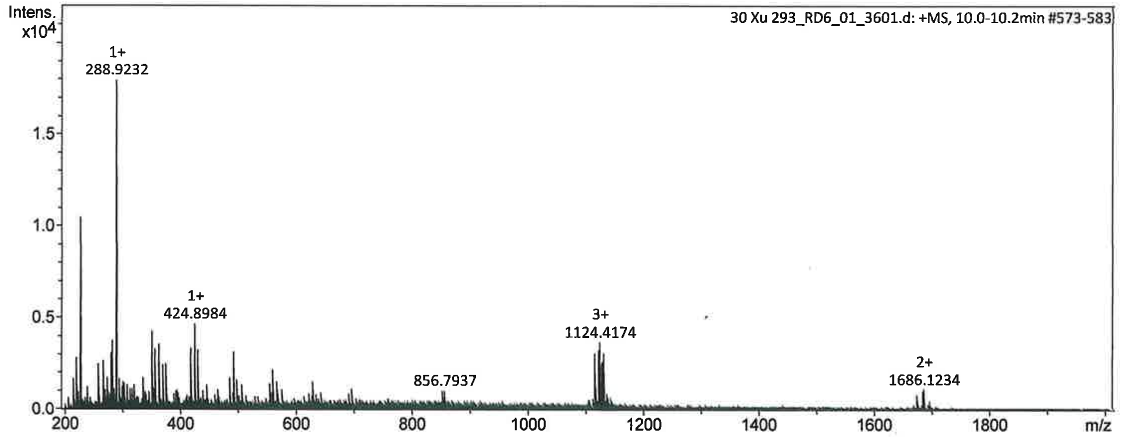

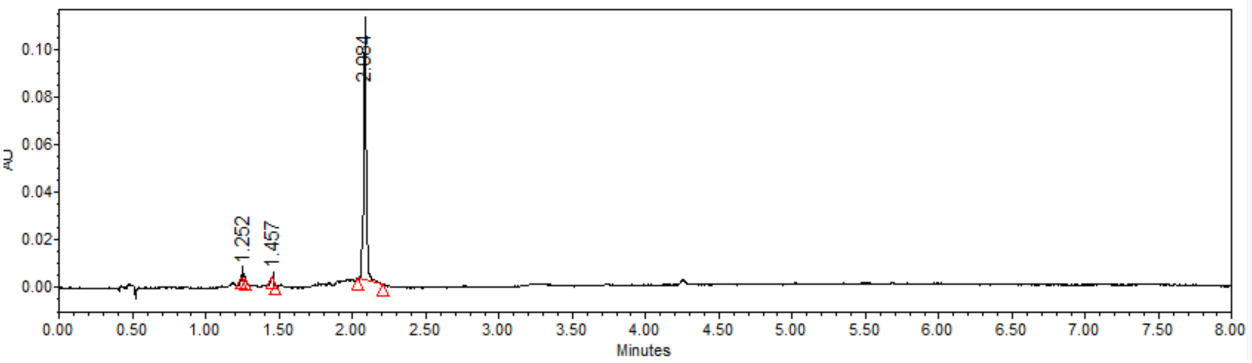

FITC-5(DOPA): FITC-(βAla)-GGGXEEEXGGGXGGGXEEEXGGGXGGGX-NH_2 (X = DOPA)

ESI-MS: calculated monoisotopic $[M+3H]^{3+}$ = 1123.748
observed monoisotopic $[M+3H]^{3+}$ = 1124.417
(*ESI-MS was used for this peptide due to high laser fragmentation of the fluorescein ground under MALDI-TOF-MS condition)

UPLC: H_2O/MeCN + 0.1% TFA, 10-95% MeCN, 5 min, 0.3 mL/min on an ACQUITY Premier CSH C18 (130 Å, 1.7 µm, 2.1 x 150mm) column.
Purity = 97.97 %

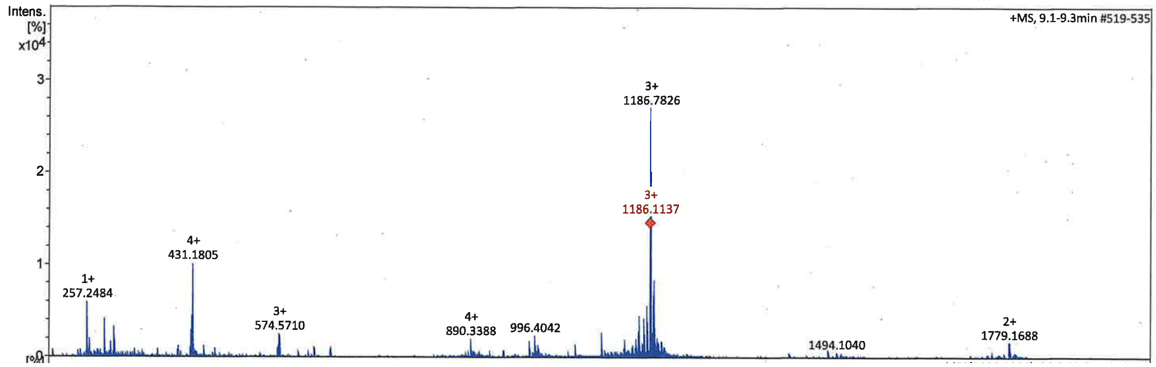

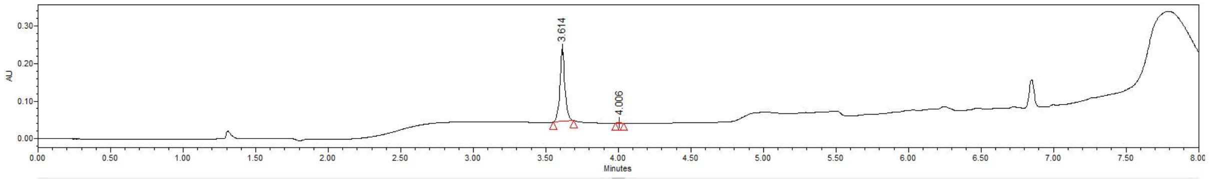

FITC-6(diOMe): `FITC-(βAla)-GGGXEEEXGGGXGGGXEEEXGGGXGGGX-NH₂ (X = diOMe)`

<u>ESI-MS</u>: calculated monoisotopic $[M+3H]^{3+}$ = 1186.100 $[M+4H]^{4+}$ = 889.825
observed monoisotopic $[M+3H]^{3+}$ = 1186.114 $[M+3H]^{3+}$ = 890.339
(*ESI-MS was used for this peptide due to high laser fragmentation of the fluorescein ground under MALDI-TOF-MS condition)

<u>UPLC</u>: H_2O/MeCN + 0.1% TFA, 10-95% MeCN, 5 min, 0.3 mL/min on an ACQUITY Premier CSH C18 (130 Å 1.7 µm, 2.1 x 150 mm) column.
Purity = 98.12 %

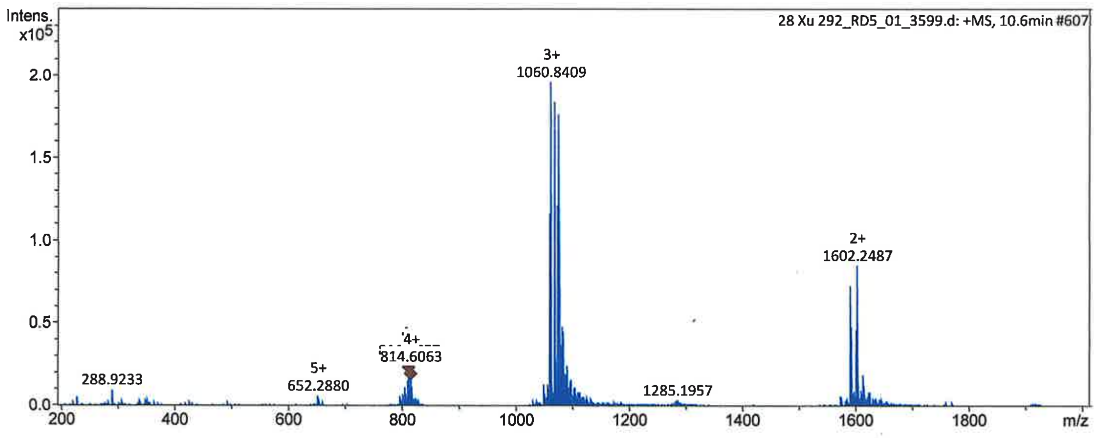

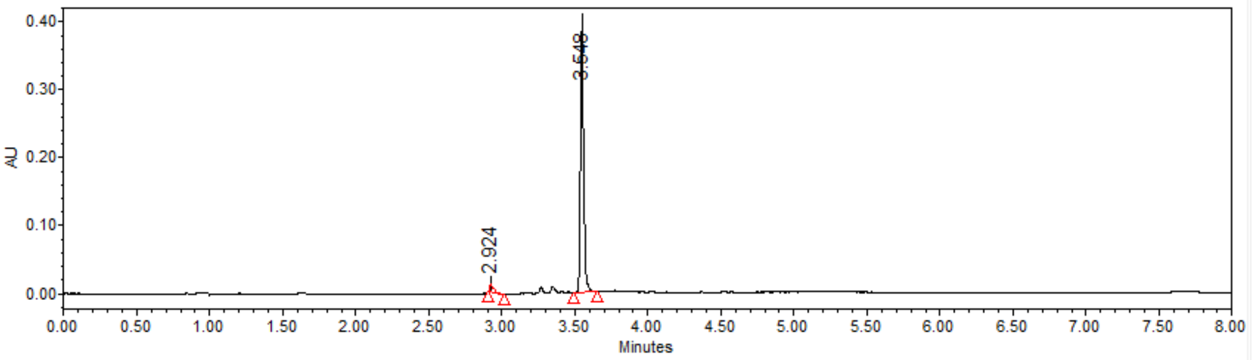

FITC-7(Cha): **`FITC-(βAla)-GGGXEEEXGGGXGGGXEEEXGGGXGGGX-NH₂   (X = Cha)`**

ESI-MS: calculated monoisotopic $[M+3H]^{3+}$ = 1060.497
observed monoisotopic $[M+3H]^{3+}$ = 1060.841
(*ESI-MS was used for this peptide due to high laser fragmentation of the fluorescein ground under MALDI-TOF-MS condition)

UPLC: H_2O/MeCN + 0.1% TFA, 10-95% MeCN, 5 min, 0.3 mL/min on an ACQUITY Premier CSH C18 (130 Å 1.7 µm, 2.1 x 150 mm) column.
Purity = 96.78 %

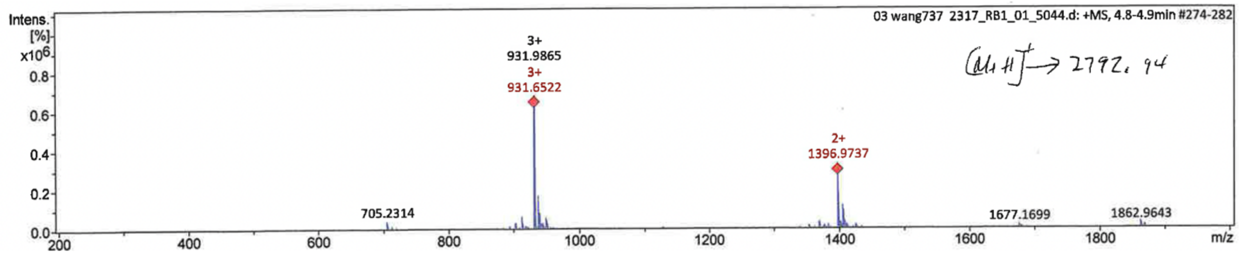

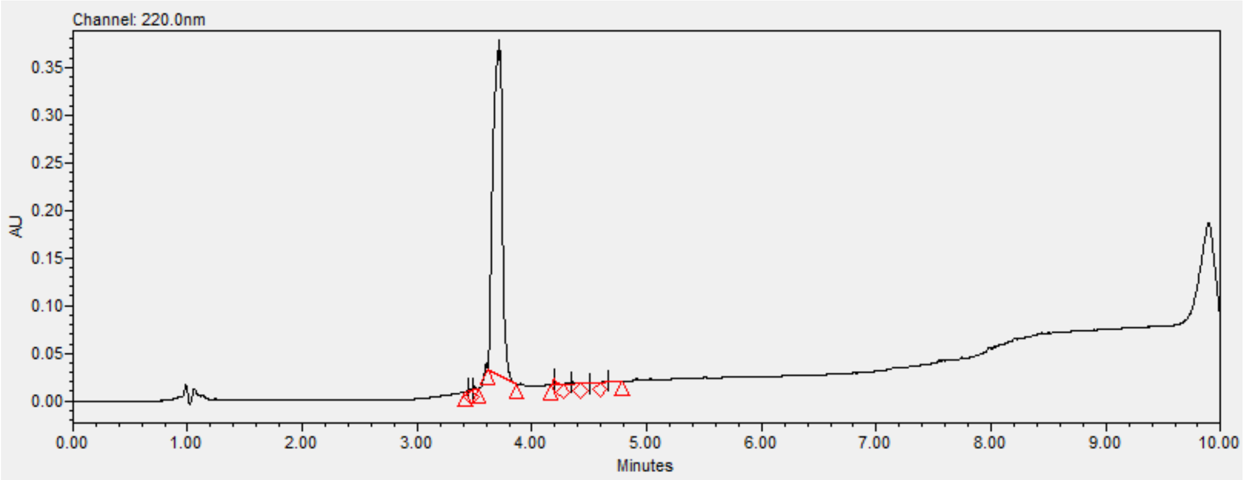

FITC-8(Ser): **`FITC-(βAla)-GGGSEEESGGGSGGGSEEESGGGSGGGY-NH₂`**

ESI-MS: calculated monoisotopic $[M+3H]^{3+}$ = 931.651 $[M+2H]^{2+}$ = 1396.974
observed monoisotopic $[M+3H]^{3+}$ = 931.652 $[M+2H]^{2+}$ = 1396.974
(*ESI-MS was used for this peptide due to high laser fragmentation of the fluorescein ground under MALDI-TOF-MS condition)

UPLC: MPA: H_2O + 10 mM ammonium formate, MPB: 9:1 (v/v %) MeCN:H_2O + 10 mM ammonium formate, 10-95% MPB, 5 min, 0.3 mL/min on an ACQUITY Premier BEH C18 (130 Å 1.7 µm, 2.1 x 150 mm) column.

Purity = 98.2 %

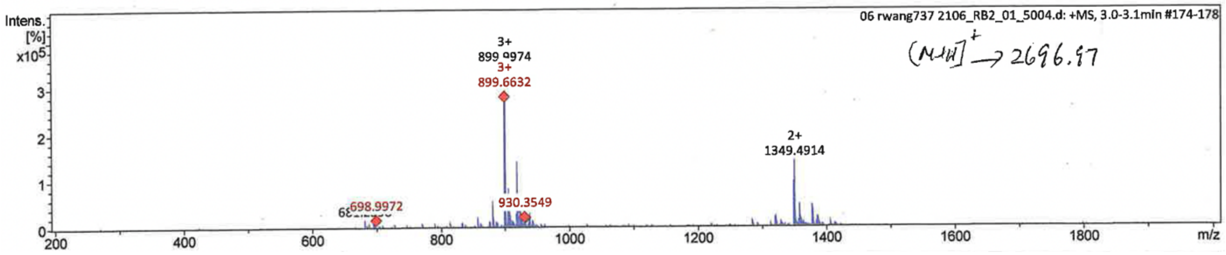

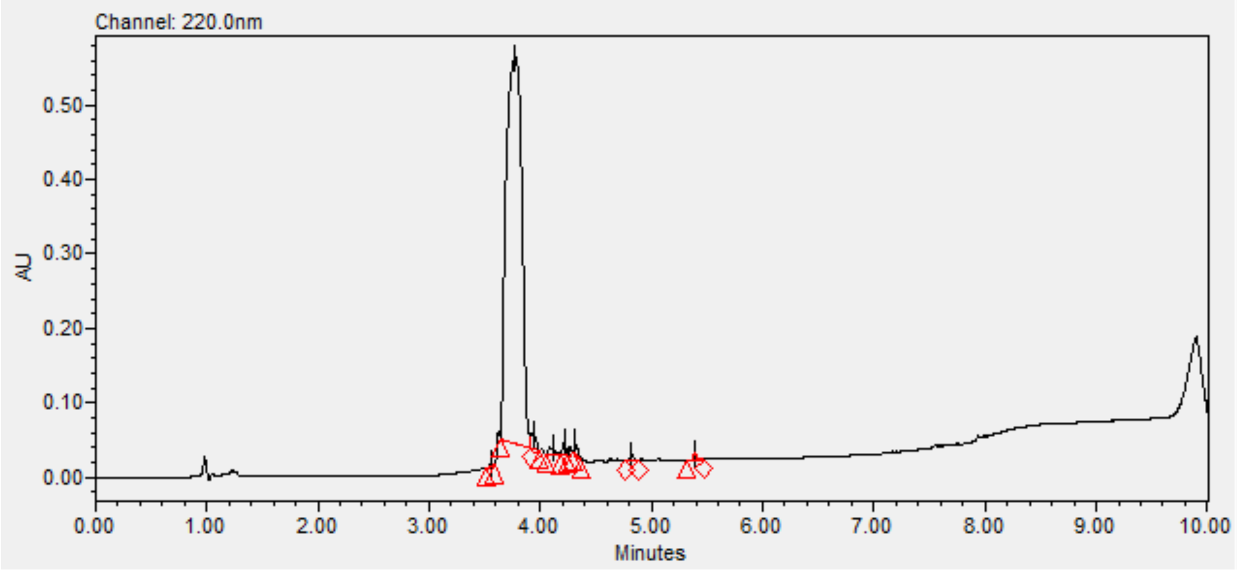

FITC-9(Ala): `FITC-(βAla)-GGGAEEEAGGGAGGGAEEEAGGGAGGGY-NH`$_2$

<u>ESI-MS</u>: calculated monoisotopic $[M+3H]^{3+}$ = 899.662
observed monoisotopic $[M+3H]^{3+}$ = 899.663
(*ESI-MS was used for this peptide due to high laser fragmentation of the fluorescein ground under MALDI-TOF-MS condition)

<u>UPLC</u>: MPA: H_2O + 10 mM ammonium formate, MPB: 9:1 (v/v %) MeCN:H_2O + 10 mM ammonium formate, 10-95% MPB, 5 min, 0.3 mL/min on an ACQUITY Premier BEH C18 (130 Å 1.7 µm, 2.1 x 150 mm) column.

Purity = 95.8 %

V. Computational Analysis

Please see additional supplementary information.

VI. Reference:

(1) Raran-Kurussi, S.; Cherry, S.; Zhang, D.; Waugh, D. S. Chapter 14 Removal of Affinity Tags with TEV Protease. *Methods Mol. Biol.* **2017**, 1586, 221-230.
(2) Schneider, C. A.; Rasband, W. S.; Eliceiri, K. W. NIH Image to ImageJ: 25 Years of Image Analysis. *Nature Methods*. **2012**, pp 671–675. https://doi.org/10.1038/nmeth.2089.
(3) Bolte, S.; Cordelières, F. P. A Guided Tour into Subcellular Colocalization Analysis in Light Microscopy. *Journal of Microscopy* **2006**, pp 213–232. https://doi.org/10.1111/j.1365-2818.2006.01706.x.
(4) Day, C. A., Kraft, L. J., Kang, M., & Kenworthy, A. K. Analysis of protein and lipid dynamics using confocal fluorescence recovery after photobleaching (FRAP). *Current protocols in cytometry*, **2012**, *Chapter 2*, Unit2.19. https://doi.org/10.1002/0471142956.cy0219s62.
(5) Taniguchi, M.; Lindsey, J. S. Database of Absorption and Fluorescence Spectra of >300 Common Compounds for use in PhotochemCAD. *Photochemistry and Photobiology* **2018**, 94 (2), 290-327. https://doi.org/10.1111/php.12860.
(6) Gaussian 16, Revision C.01, Frisch, M. J.; Trucks, G. W.; Schlegel, H. B.; Scuseria, G. E.; Robb, M. A.; Cheeseman, J. R.; Scalmani, G.; Barone, V.; Petersson, G. A.; Nakatsuji, H.; Li, X.; Caricato, M.; Marenich, A. V.; Bloino, J.; Janesko, B. G.; Gomperts, R.; Mennucci, B.; Hratchian, H. P.; Ortiz, J. V.; Izmaylov, A. F.; Sonnenberg, J. L.; Williams-Young, D.; Ding, F.; Lipparini, F.; Egidi, F.; Goings, J.; Peng, B.; Petrone, A.; Henderson, T.; Ranasinghe, D.; Zakrzewski, V. G.; Gao, J.; Rega, N.; Zheng, G.; Liang, W.; Hada, M.; Ehara, M.; Toyota, K.; Fukuda, R.; Hasegawa, J.; Ishida, M.; Nakajima, T.; Honda, Y.; Kitao, O.; Nakai, H.; Vreven, T.; Throssell, K.; Montgomery, J. A., Jr.; Peralta, J. E.; Ogliaro, F.; Bearpark, M. J.; Heyd, J. J.; Brothers, E. N.; Kudin, K. N.; Staroverov, V. N.; Keith, T. A.; Kobayashi, R.; Normand, J.; Raghavachari, K.; Rendell, A. P.; Burant, J. C.; Iyengar, S. S.; Tomasi, J.; Cossi, M.; Millam, J. M.; Klene, M.; Adamo, C.; Cammi, R.; Ochterski, J. W.; Martin, R. L.; Morokuma, K.; Farkas, O.; Foresman, J. B.; Fox, D. J. Gaussian, Inc., Wallingford CT **2016**.
(7) Polik, W. F.; Schmidt, J. R. WebMO: Web-based computational chemistry calculations in education and research. *WIREs Comput Mol Sci* **2021**, 12 (1), e1554. https://doi.org/10.1002/wcms.1554.
