# Supplementary figures and images for: Interplay of Noncovalent Interactions in Phase Separation Mediated by Tyrosine-Rich and Arginine-Rich Polypeptides
Source: J Am Chem Soc. 2026 Jun 17;148(25):26585–98. doi: 10.1021/jacs.6c06972 (PMC13339138; doi:10.1021/jacs.6c06972)

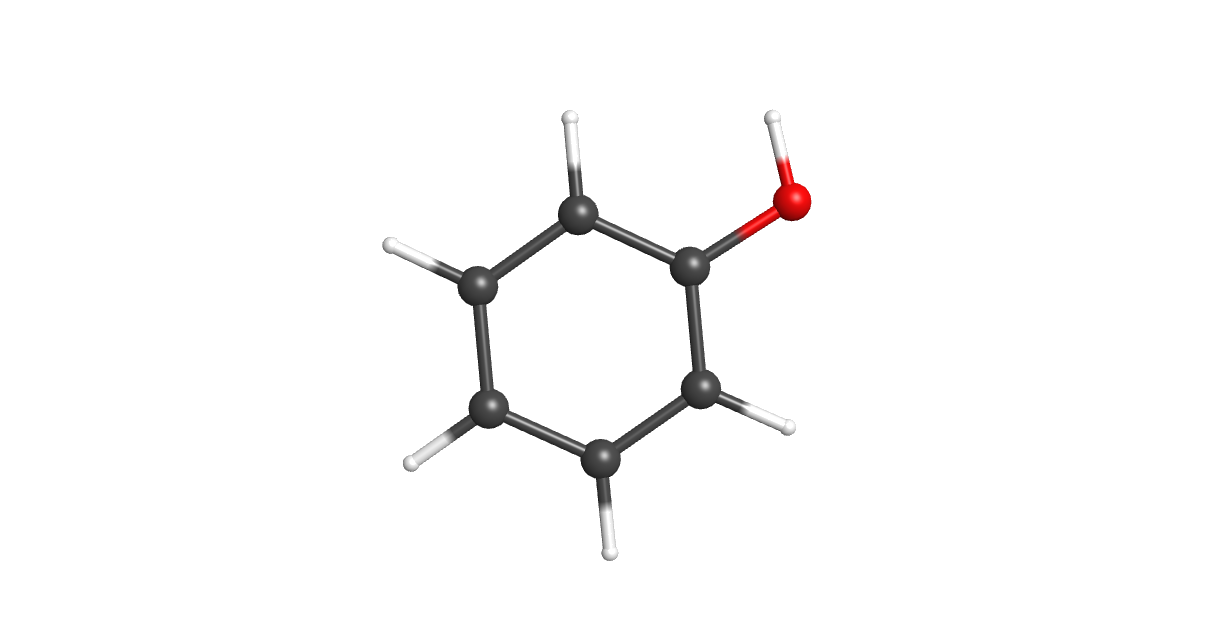

Supplement: Supplementary file 2 [file ja6c06972_si_002.zip › pics/A'.png]

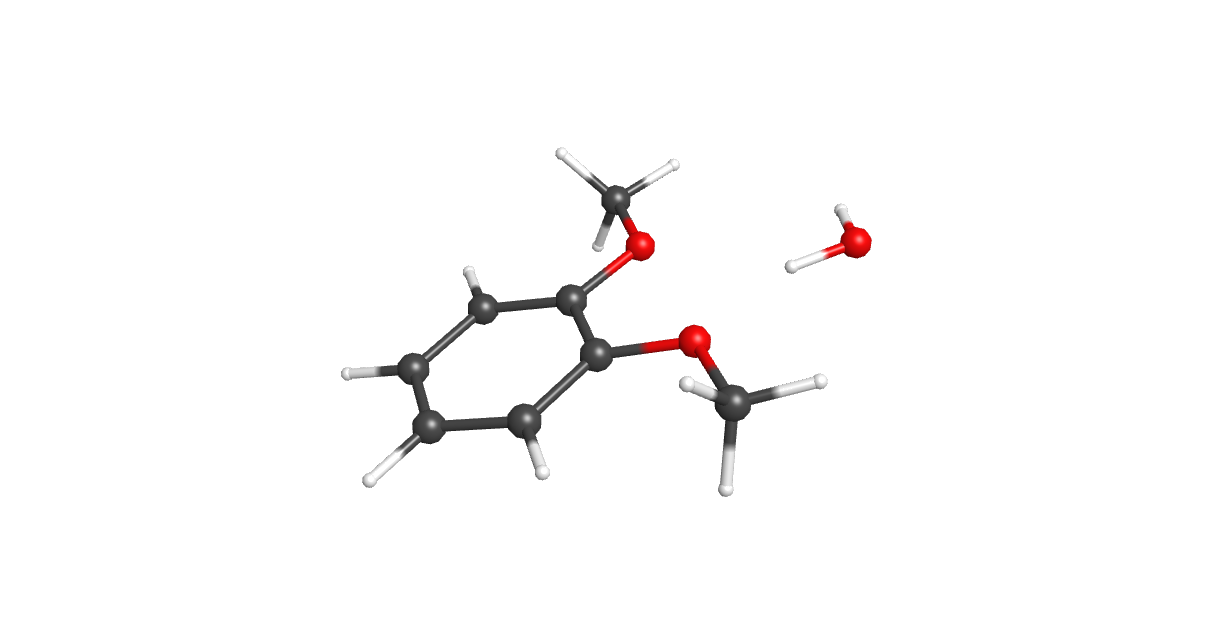

Supplement: Supplementary file 2 [file ja6c06972_si_002.zip › pics/G'.png]

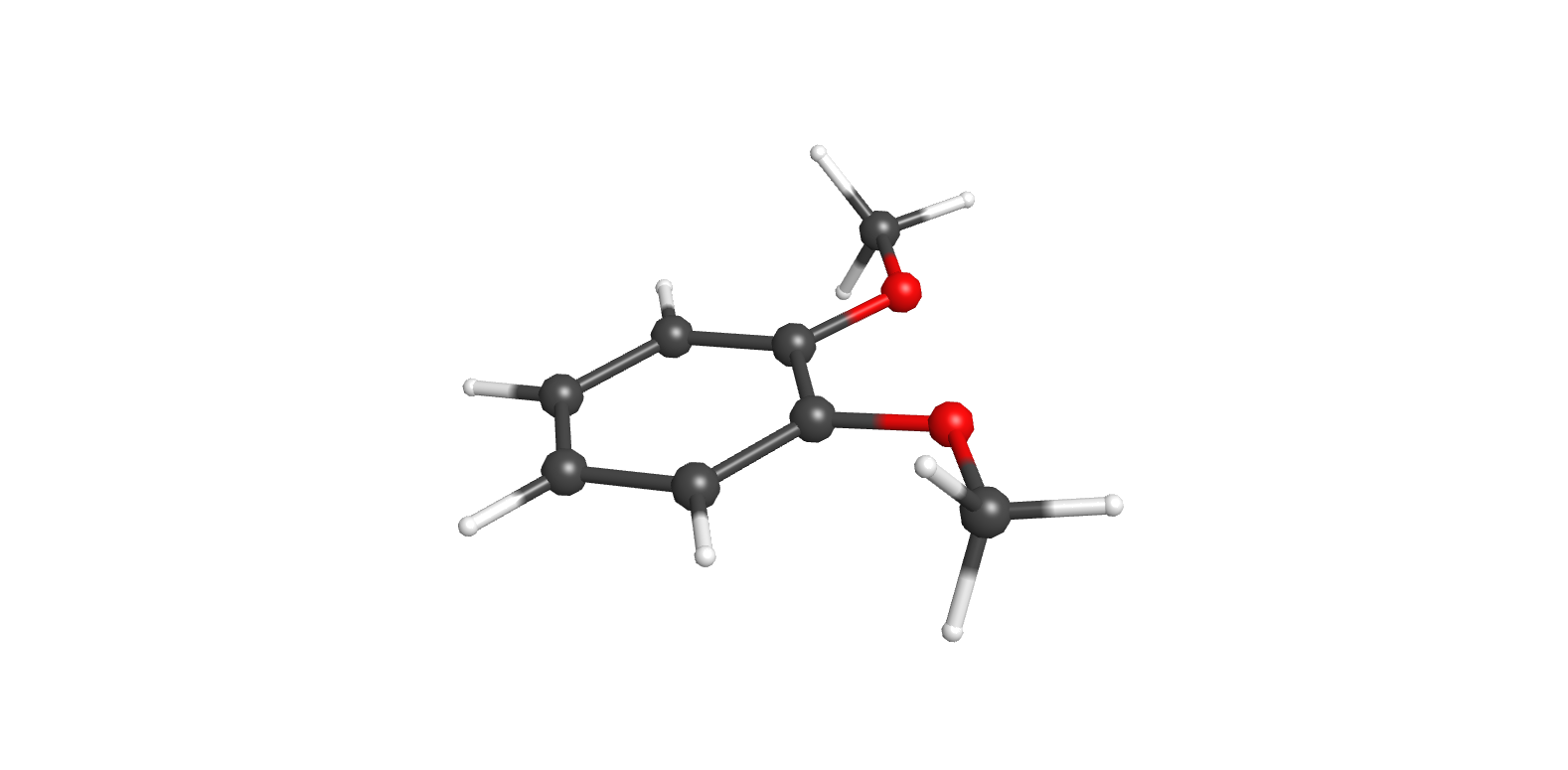

Supplement: Supplementary file 2 [file ja6c06972_si_002.zip › pics/F'.png]

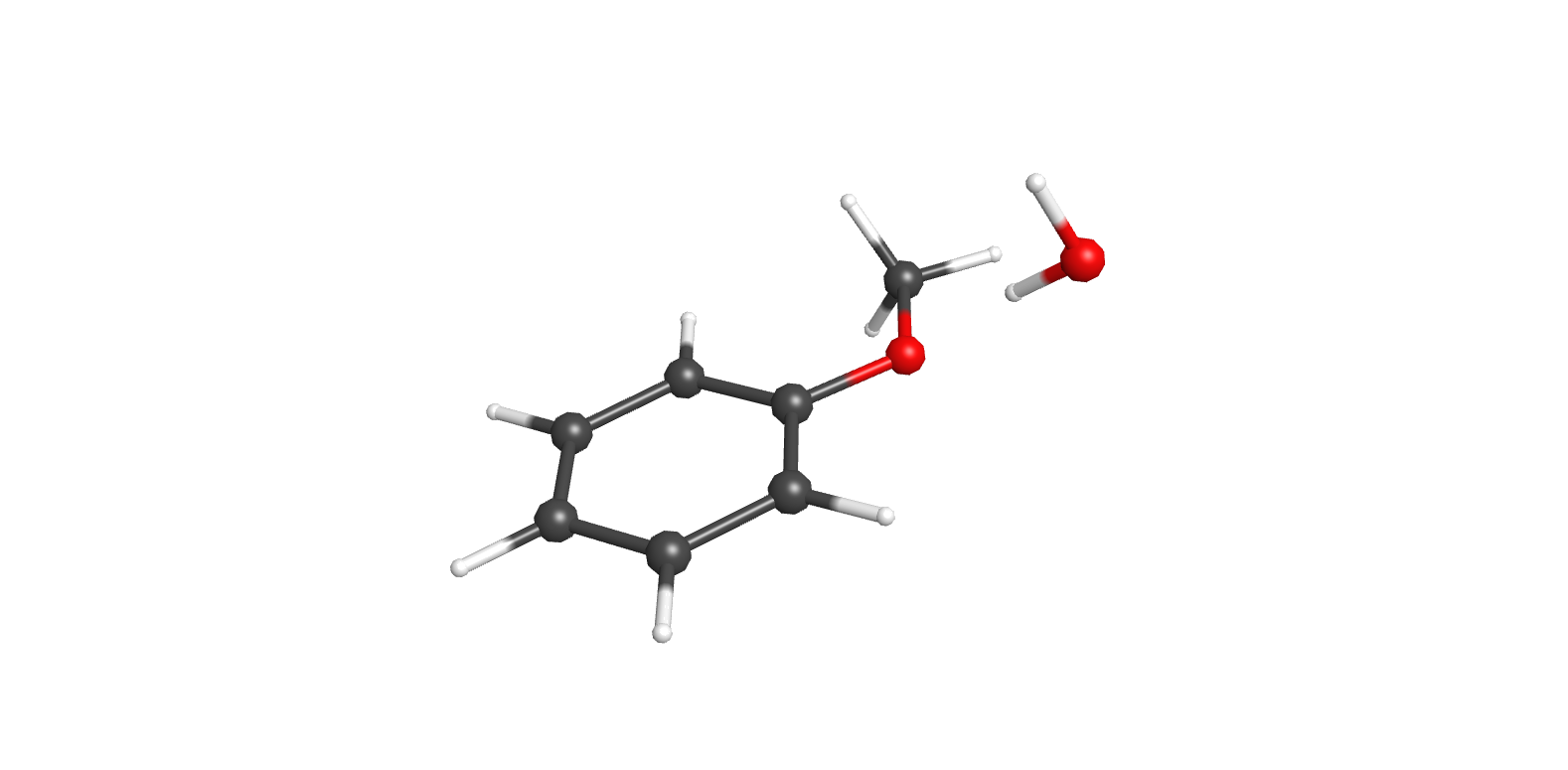

Supplement: Supplementary file 2 [file ja6c06972_si_002.zip › pics/E'.png]

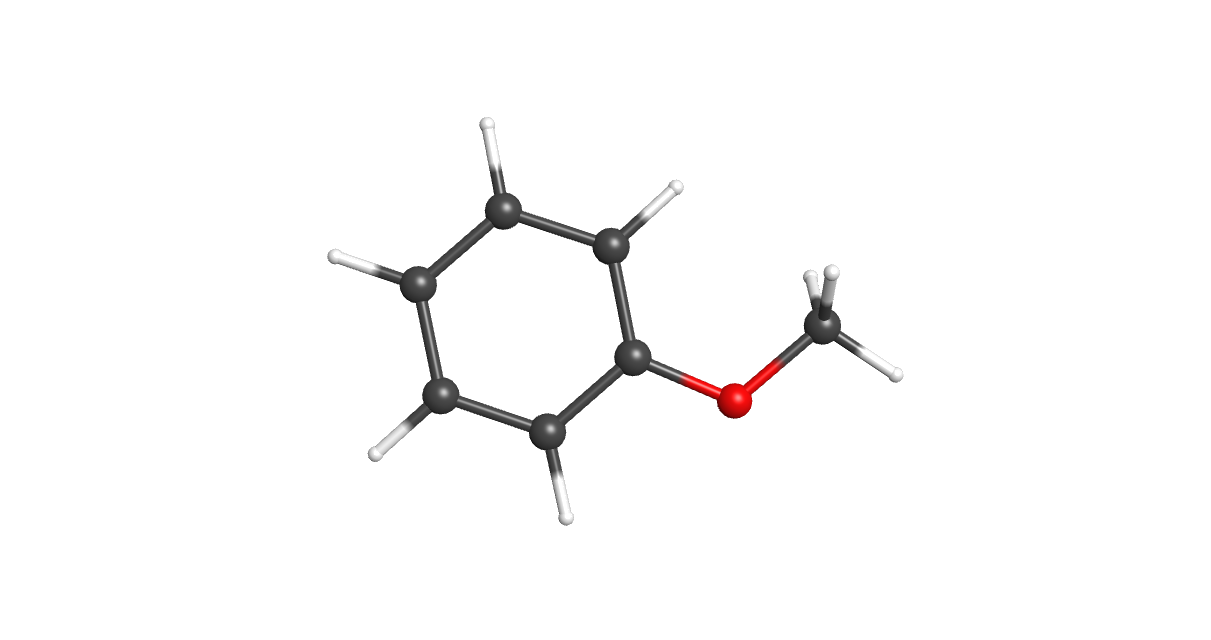

Supplement: Supplementary file 2 [file ja6c06972_si_002.zip › pics/D'.png]

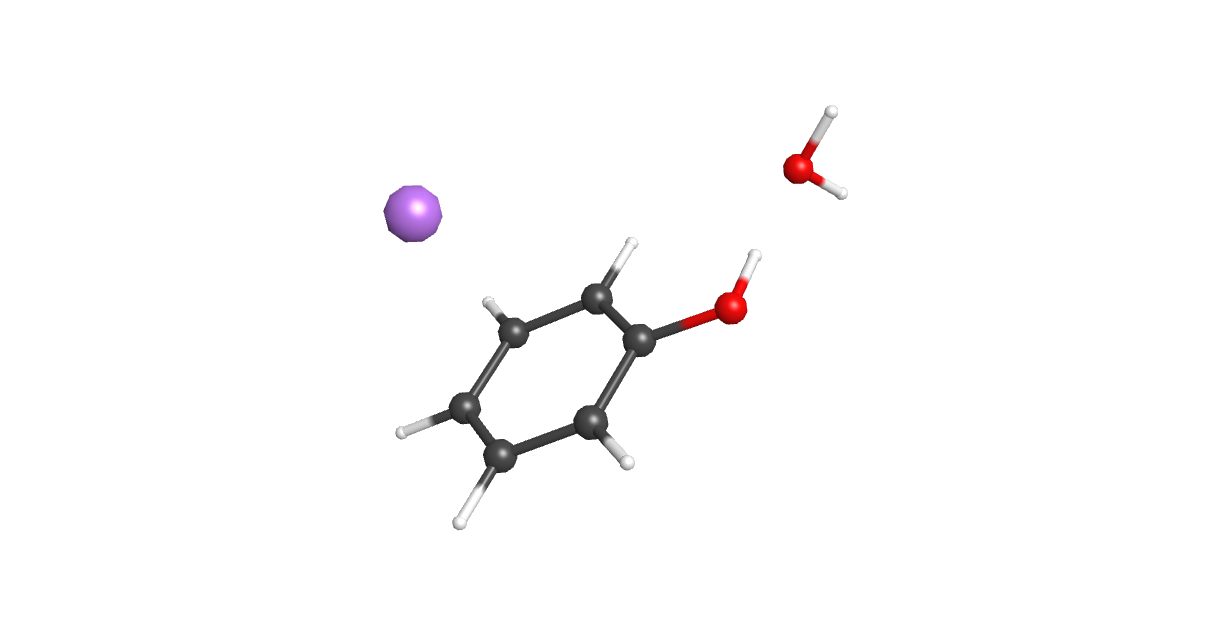

Supplement: Supplementary file 2 [file ja6c06972_si_002.zip › pics/B.png]

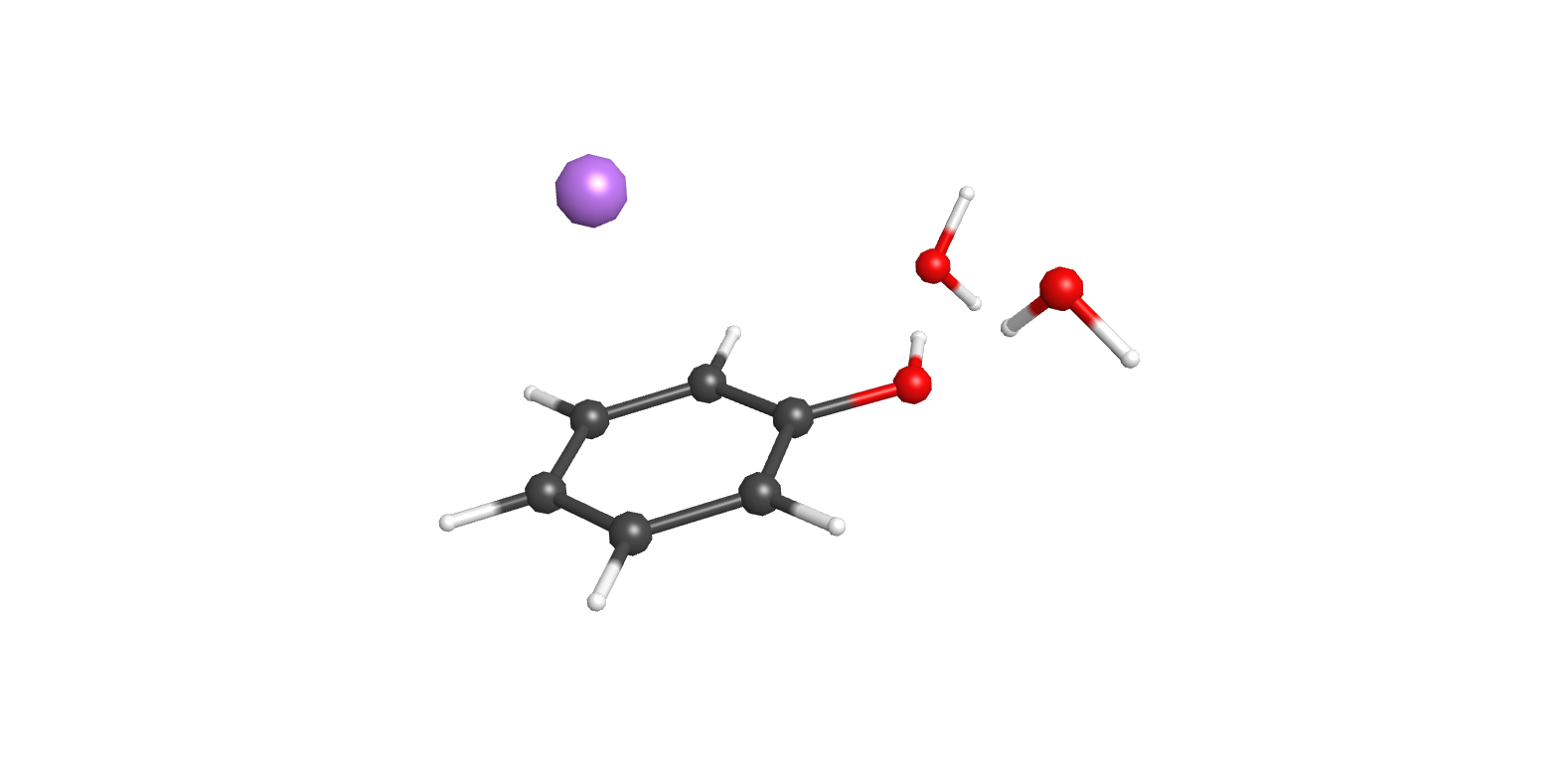

Supplement: Supplementary file 2 [file ja6c06972_si_002.zip › pics/C.png]

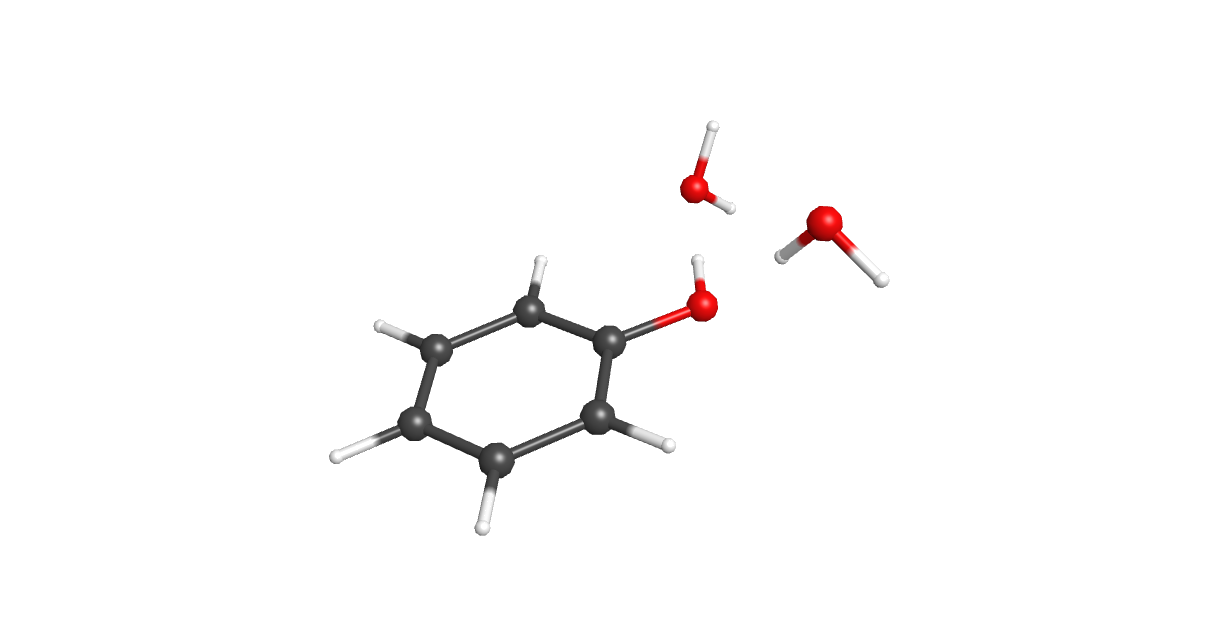

Supplement: Supplementary file 2 [file ja6c06972_si_002.zip › pics/C'.png]

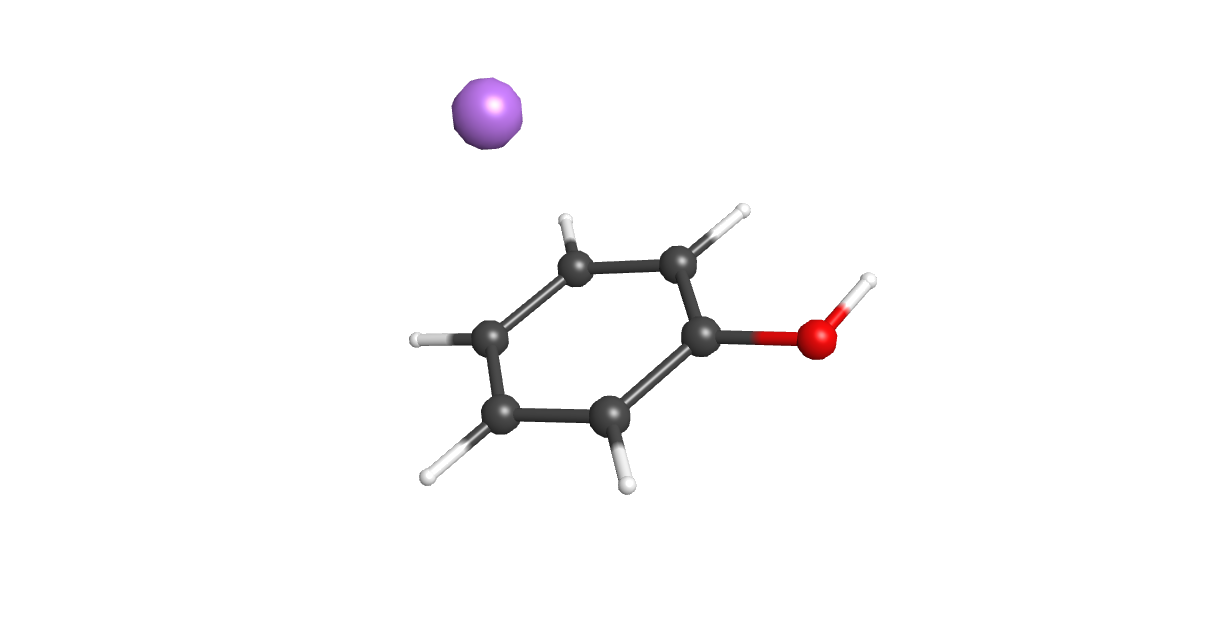

Supplement: Supplementary file 2 [file ja6c06972_si_002.zip › pics/A.png]

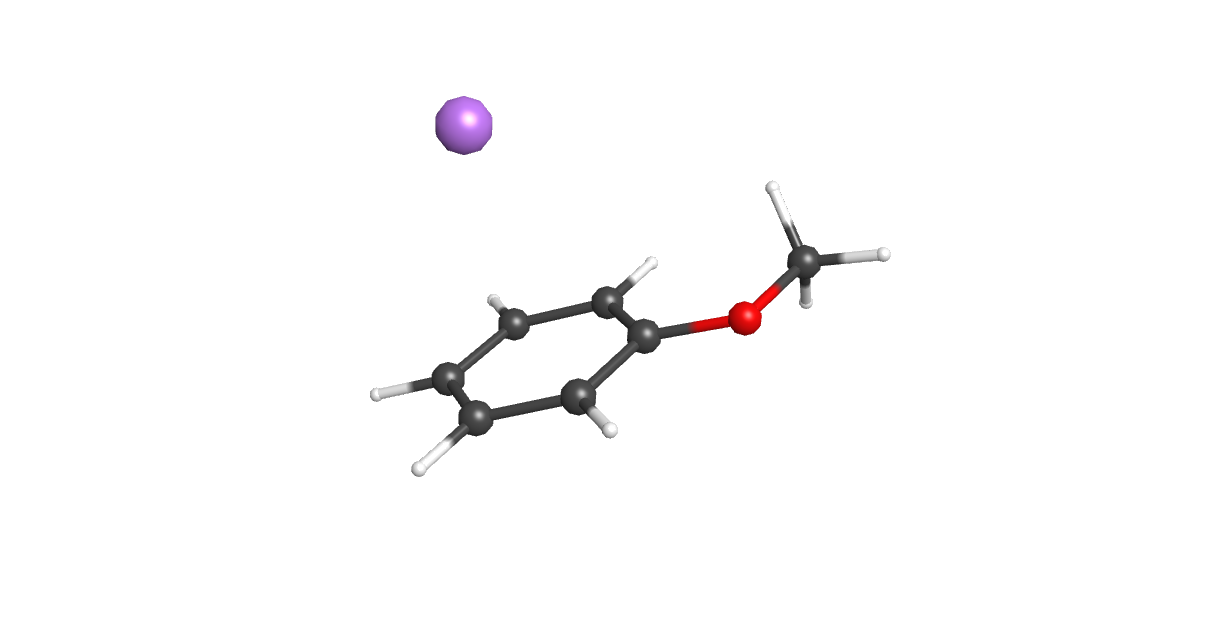

Supplement: Supplementary file 2 [file ja6c06972_si_002.zip › pics/D.png]

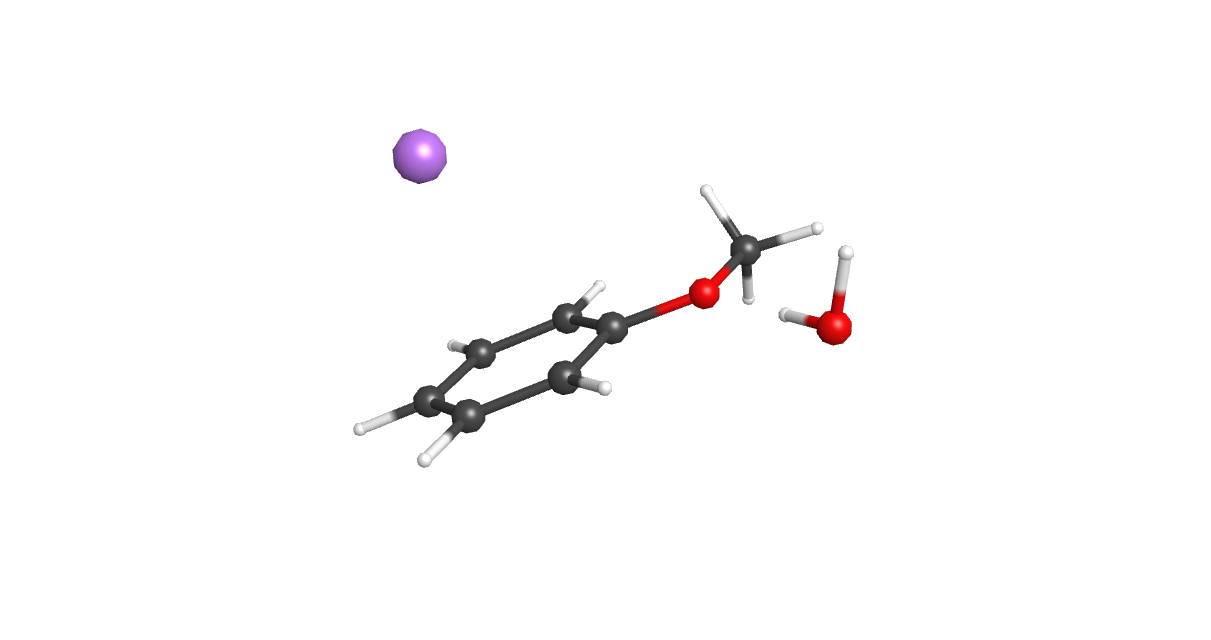

Supplement: Supplementary file 2 [file ja6c06972_si_002.zip › pics/E.png]

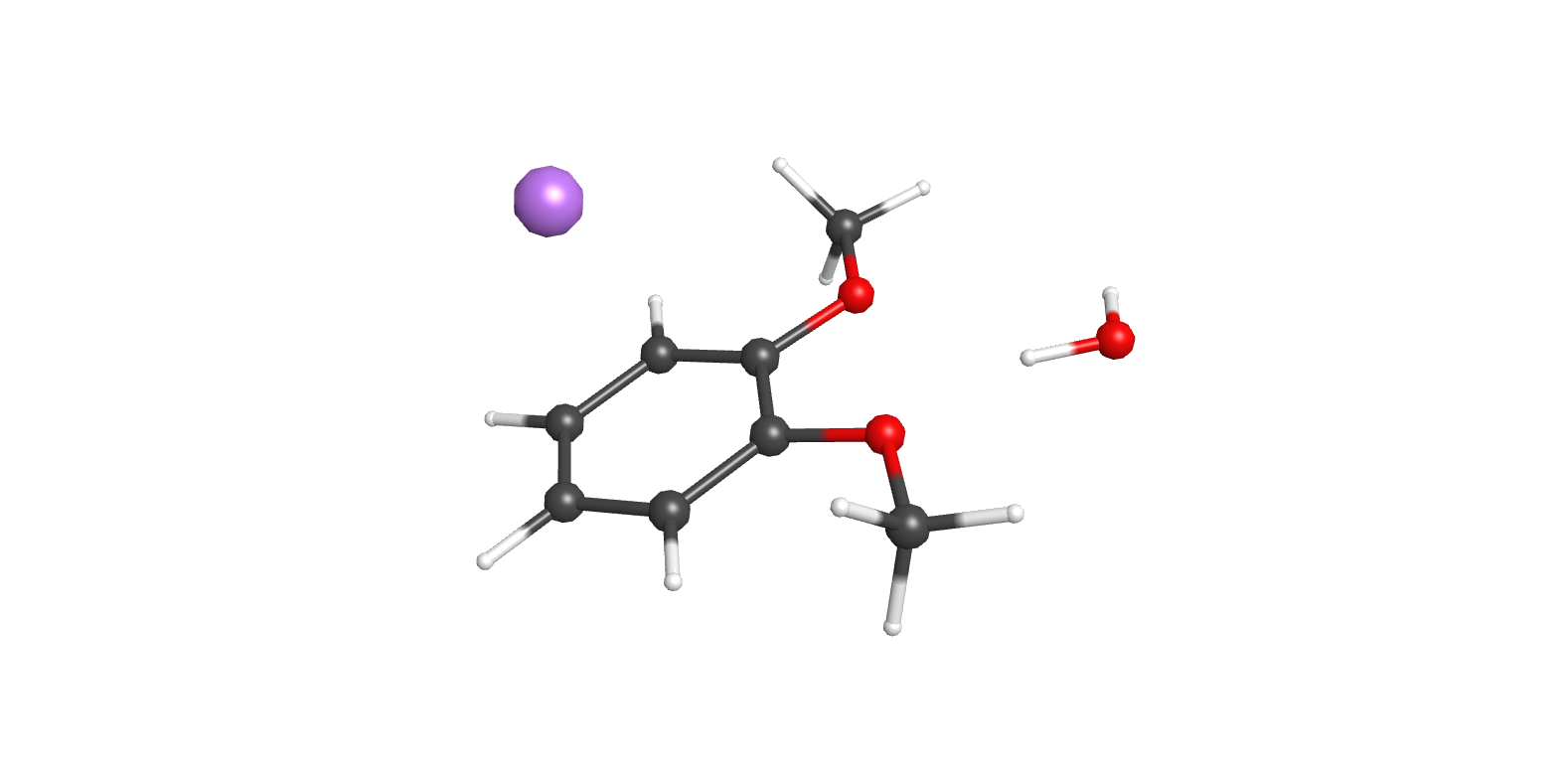

Supplement: Supplementary file 2 [file ja6c06972_si_002.zip › pics/G.png]

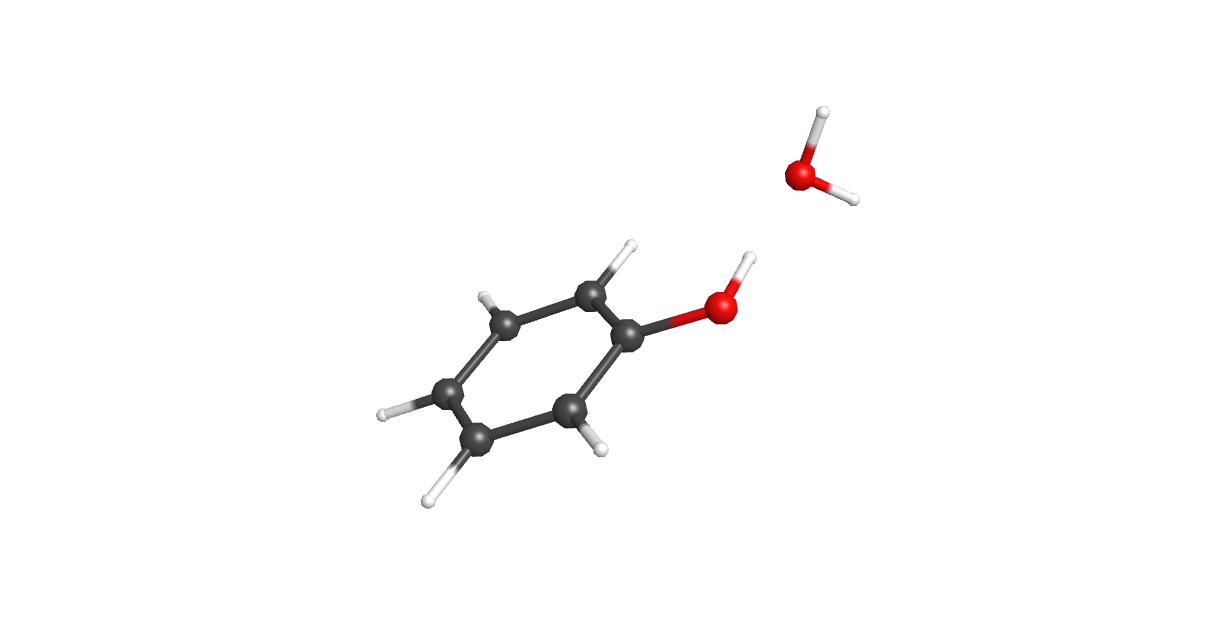

Supplement: Supplementary file 2 [file ja6c06972_si_002.zip › pics/B'.png]

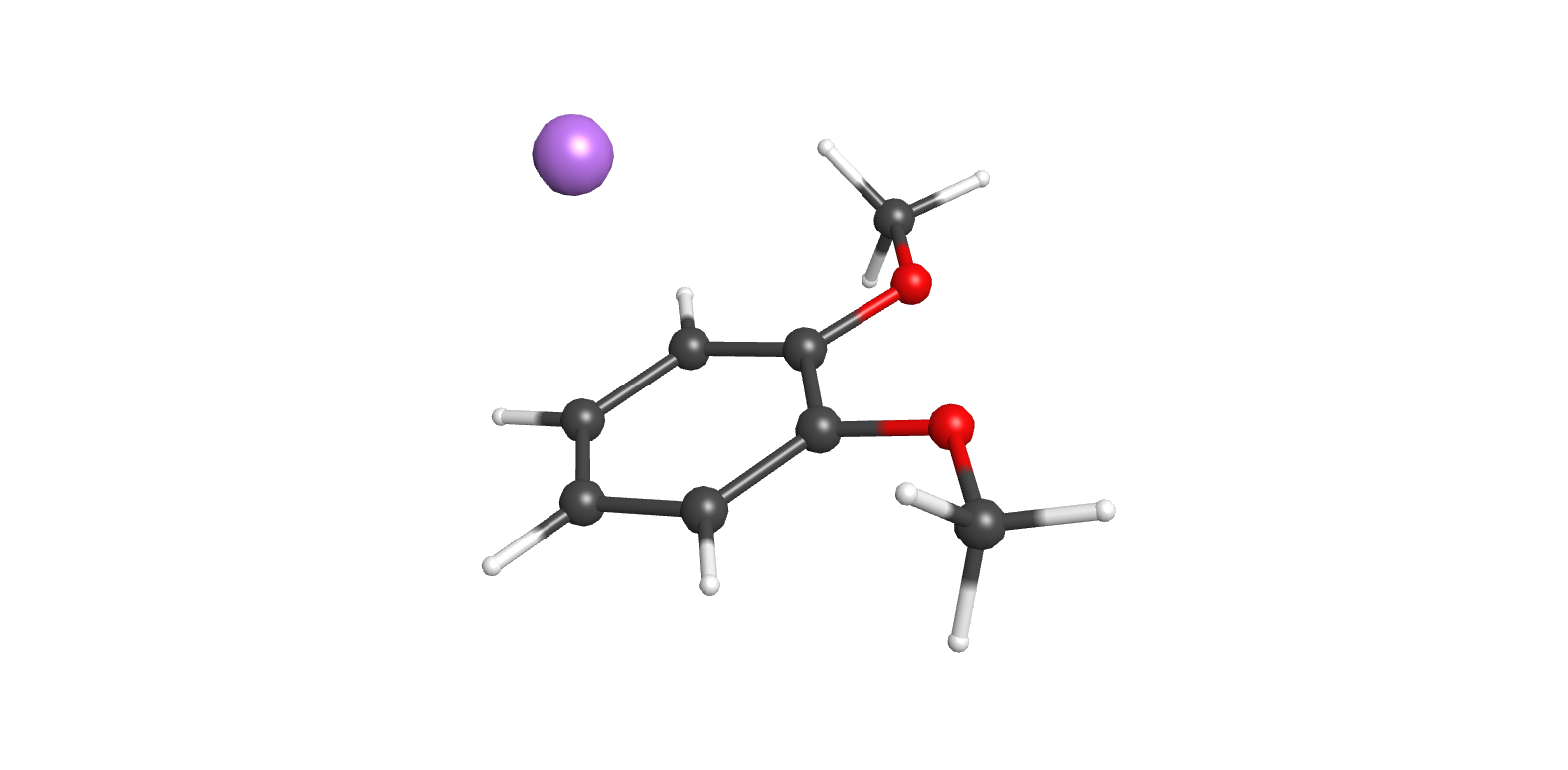

Supplement: Supplementary file 2 [file ja6c06972_si_002.zip › pics/F.png]
